# A systematic review with meta‐analysis of heritability estimates for temperament‐related traits in beef and dairy cattle populations

**DOI:** 10.1111/jbg.12874

**Published:** 2024-05-29

**Authors:** Luís Fernando Batista Pinto, Bruno Delphino Medrado, Victor Breno Pedrosa, Luiz F. Brito

**Affiliations:** ^1^ Department of Animal Sciences Federal University of Bahia Salvador Brazil; ^2^ Department of Animal Sciences Purdue University West Lafayette Indiana USA; ^3^ Federal Institute Baiano – Campus Santa Inês Santa Inês Brazil; ^4^ Neogen Corporation – Biotechnology Research Lincoln Nebraska USA

**Keywords:** behaviour, cattle, docility, genetic parameters, selection

## Abstract

Temperament (docility) is a key breeding goal in the cattle industry due to its direct relationship with animal welfare, cattle handler's safety and animal productivity. Over the past six decades, numerous studies have reported heritability estimates for temperament‐related traits in cattle populations ranging from low to high values. Therefore, the primary objective of this study was to perform a comprehensive systematic review with meta‐analysis to obtain weighted estimates of heritability for temperament‐related traits in worldwide cattle populations. After data editing and quality control, 106 studies were included in the systematic review, of which 29.2% and 70.8% reported estimates of heritability for temperament‐related traits in dairy and beef cattle populations, respectively. Meta‐analyses were performed for 95 heritability estimates using a random model approach. The weighted heritability estimates were as follow: (a) flight score at weaning = 0.23 (95% CI: 0.15–0.32); (b) flight speed at weaning = 0.30 (95% CI: 0.26–0.33); (c) joint analysis of flight speed and flight score at weaning = 0.27 (95% CI: 0.22–0.31); (d) flight speed at yearling = 0.26 (95% CI: 0.21–0.30); (e) joint analysis of flight speed at weaning and yearling = 0.27 (95% CI: 0.24–0.30); (f) movement score = 0.12 (95% CI: 0.08–0.15); (g) crush score at weaning = 0.21 (95% CI: 0.17–0.25); (h) pen score at weaning = 0.27 (95% CI: 0.19–0.34); (i) pen score at yearling = 0.20 (95% CI: 0.17–0.23); (j) joint analysis of pen score at weaning and yearling = 0.22 (95% CI: 0.18–0.26); (k) cow's aggressiveness at calving = 0.10 (95% CI: 0.01–0.19); (l) general temperament = 0.13 (95% CI: 0.06–0.19); (m) milking temperament = 0.16 (95% CI: 0.11–0.21); and (n) joint analysis of general and milking temperament = 0.14 (95% CI: 0.11–0.18). The heterogeneity index ranged from 0% to 77%, and the Q‐test was significant (*p* < 0.05) for four single‐trait meta‐analyses. In conclusion, temperament is moderately heritable in beef cattle populations, and flight speed at weaning had the highest weighted heritability estimate. Moreover, between‐study heterogeneity was low or moderate in beef cattle traits, suggesting reasonable standardization across studies. On the other hand, low‐weighted heritability and high between‐study heterogeneity were estimated for temperament‐related traits in dairy cattle, suggesting that more studies are needed to better understand the genetic inheritance of temperament in dairy cattle populations.

## 1 INTRODUCTION

Cattle temperament can be defined as the animal's response to handling. Some historical beef cattle studies proposed assessing the magnitude of animal response in situations involving human interaction (e.g., during weighting or handling in a corral). Tulloh (1961) proposed to assess the level of animal agitation during confinement in the bail, where lower and higher subjective scores indicate docile and aggressive animals, respectively. Today, this method is usually termed chute or crush score. In addition, Burrow et al. (1988) proposed an objective method called flight speed, which assesses the velocity at which an animal leaves the scale after weighting. This method assumes that docile animals move away more slowly than aggressive animals. Both methods evaluate the animal's response under restrained conditions. On the other hand, Boivin et al. (1992) proposed an unrestrained test that includes two steps. First, the animal is separated from their social group and kept in the corner of a pen for some time with a person near the animal. This method is often termed pen score or docility score.

According to Pott (1918), assessments of dairy temperament were already performed in the 19th century, with different definitions based on indicators such as eye full and expressive, clean face, large nostrils, long and light neck, sharp withers and prominent spinal column. Later, the farmer or milking staff assessed the response to the whole milking procedure, where lower and higher scores indicate poor and good milking temperament, respectively (Haskell et al., 2014). Thus, score traits have been used in the genetic evaluations of several dairy cattle breeds worldwide since the 1980s and 1990s (Chang et al., 2020). Due to the subjectivity of animal behaviour assessed by humans, there still needs to be a consensus on the best methods to assess temperament in dairy cattle. Therefore, new methods continue to be presented over time (e.g., Danchuk et al., 2020; Neave et al., 2022). Moreover, with more recent advances in Precision Livestock Farming (PLF) practices, novel traits can be derived from data generated by video recording, pedometers, activity collars and automatic milking system‐derived traits (Chang et al., 2020; Pedrosa et al., 2024). The oldest and the latest methods show a positive association between good temperament and greater production in dairy cattle (Neave et al., 2022).

Temperament‐related traits are essential breeding goals in cattle breeding programs as more reactive animals tend to have poorer welfare, cause more accidents when handled by humans, and have lower productivity than less reactive animals (Burrow, 2001; Haskell et al., 2014; King et al., 2006). Although temperament is relevant to cattle production systems, few worldwide breeding programs have included temperament in their selection schemes (Chang et al., 2020).

Measuring a large number of individuals and estimating variance components and genetic parameters are important steps for introducing temperament‐related traits in modern selection indexes. Over the last 60 years, numerous studies have reported heritability estimates for temperament‐related traits in beef and dairy cattle populations (e.g., Alvarenga et al., 2022, 2023; O'Bleness et al., 1960), and the estimates ranged from 0.02 (Gauly et al., 2002; Vallée et al., 2015) to 0.70 (Fordyce et al., 1996). The large variation in heritability estimates across studies can be due to genetic (i.e., population parameters) and non‐genetic factors (i.e., systematic effects). Moreover, the number of animals with data in each study varied across studies. A small sample size (*N* < 1000) was observed in most studies, which resulted in wider 95% CIs for the heritability estimates. In this context, a meta‐analysis can yield weighted estimates with smaller confidence intervals, i.e., a more reliable heritability estimate.

Meta‐analyses of different studies considering ‘study’ as a random effect are an alternative for obtaining weighted estimates (Borenstein et al., 2010). Meta‐analysis produces a weighted and more accurate estimate by combining the estimates reported across studies while considering the heterogeneity among the studies (Dawson et al., 2016). Furthermore, meta‐analysis allows the study of the between‐study heterogeneity, which cannot be evaluated in single studies. The weighted heritability estimates obtained from meta‐analysis can be used as a guide for the design of breeding programs, including the target traits as selection criteria. Thus, meta‐analysis related to estimating genetic parameters for various traits has been published in beef and dairy cattle (e.g., Gathura et al., 2020; Maskal et al., 2024; Oliveira et al., 2018). However, these studies did not include temperament‐related traits. Therefore, the primary objective of this study was to obtain weighted heritability estimates for temperament‐related traits in beef and dairy cattle based on a random effect model meta‐analysis.

## 2 MATERIALS AND METHODS

### 2.1 Data collection

Various databases were used to perform a systematic review to identify bibliographic references reporting heritability estimates for temperament‐related traits in cattle, including PubMed, Web of Science, ScienceDirect and Scopus. The search was carried out on September 20th, 2023, using the following search parameters: (1) *Web of Science*: Heritability OR Genetic Parameters (Topic) AND Cattle OR Bovine OR Heifer OR Calves OR *Bos taurus* OR *Bos indicus* (Topic) AND Temperament OR Docility OR Crush OR Chute OR Aggressiveness OR Disposition OR Flight OR Behaviour OR Pen (Topic) NOT Pig OR Horse OR Sheep OR Goat OR Boar (Title) NOT feeding behaviour (Topic); (2) *Scopus*: TITLE‐ABS‐KEY (heritability OR genetic AND parameters) AND TITLE‐ABS‐KEY (cattle OR bovine OR heifer OR calves OR taurus OR indicus) AND TITLE‐ABS‐KEY (temperament OR docility OR crush OR chute OR aggressiveness OR disposition OR flight OR behaviour OR pen) AND NOT TITLE (pig OR horse OR sheep OR goat OR boar) AND NOT TITLE‐ABS‐KEY (feeding AND behaviour)); (3) *PubMed*: ((((heritability[Title/Abstract] OR genetic parameters[Title/Abstract]) AND (cattle[Title/Abstract] OR bovine[Title/Abstract] OR heifer[Title/Abstract] OR calves[Title/Abstract] OR *Bos taurus*[Title/Abstract] OR *Bos indicus*[Title/Abstract])) AND (temperament[Title/Abstract] OR docility[Title/Abstract] OR crush[Title/Abstract] OR chute[Title/Abstract] OR aggressiveness[Title/Abstract] OR Disposition[Title/Abstract] OR Flight[Title/Abstract] OR Behaviour[Title/Abstract] OR Pen[Title/Abstract])) NOT (Pig[Title] OR Horse[Title] OR Sheep[Title] OR Goat[Title] OR Boar[Title])) NOT (feeding behaviour[Title/Abstract]); and (4) *ScienceDirect*: Find articles with these terms: cattle OR bovine OR heifer OR calves OR *Bos taurus* OR *Bos indicus*; Title, abstract or author‐specified keywords: (heritability OR genetic parameters) AND (temperament OR docility OR crush OR chute OR aggressiveness). Previous literature reviews about cattle temperament (e.g., Chang et al., 2020; Haskell et al., 2014) were used to define the keywords used in our search strategy.

A total of 886 studies (scientific papers published in scientific journals or manuscripts published in conference proceedings) were retrieved from these databases, saved, and imported to the Rayyan tool (Ouzzani et al., 2016), which enabled the identification of duplicated references. Subsequently, 175 duplicated studies were excluded from subsequent analyses. Subsequently, manual screening of the title and abstract of the 711 remaining references was performed. A total of 638 references were excluded based on the following criteria: (1) the study did not report cattle (*Bos taurus ssp*.) heritability estimates (i.e., a study from another species); or (2) the study did not report heritability estimates for temperament‐related traits. The reference section of each remaining study (*n* = 73) was checked, and 33 additional studies were identified, many of which were published many years ago and not always available in indexed databases. Therefore, detailed data (full‐text screening) was collected from 106 studies.

**FIGURE 1:**
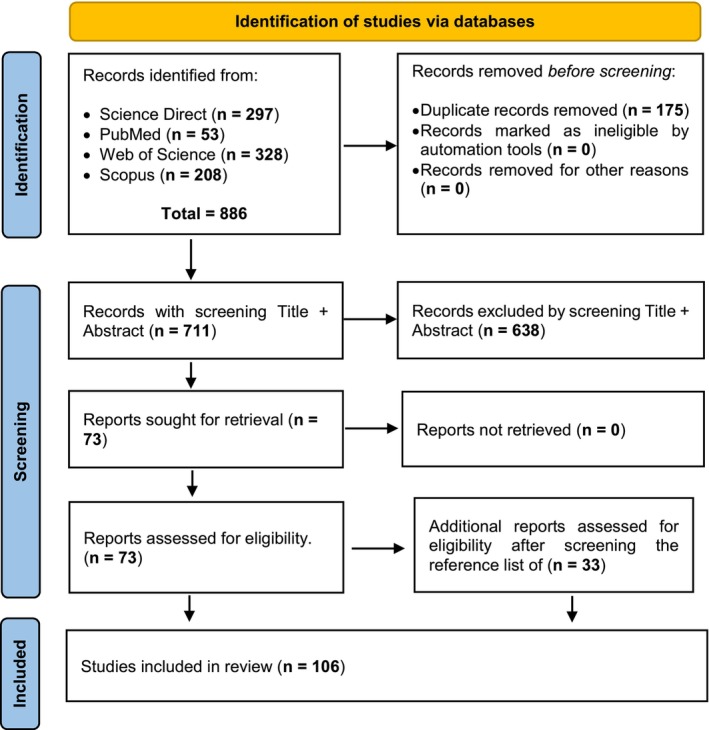
PRISMA flow diagram adapted from Page et al. (2021). [Colour figure can be viewed at wileyonlinelibrary.com]

Only studies with both heritability estimates and their standard errors and a complete trait description were kept in the dataset for subsequent analyses. We extracted the following variables from all studies (both selected and excluded): first author's last name, year of publication, breed type (dairy or beef), and the country of origin of the studied population. From the selected studies, the additional variables collected included heritability estimates and their standard errors, sample size, breed(s), name and description of the temperament‐related traits, sex and age of the animals at recording, and the statistical method used to estimate the variance components. Figure 1 presents a PRISMA flow diagram of the systematic review performed in this study following the figure structure as reported by Page et al. (2021).

### 2.2 Meta‐analysis

The meta‐analysis was performed using the meta‐R package (https://cran.r‐project.org/web/packages/meta/meta.pdf), which fitted the following random effect model:

$${\hat{\theta }}_{k}=\mu +\zeta_{k}+\epsilon_{k},$$

where ${\hat{\theta }}_{k}$ is the estimate of the heritability published in the $k^{\mathrm{th}}$ study, μ is the weighted heritability in the population, $\zeta_{k}$ is the random effect of study with $\zeta_{k}\sim N\left(0,\tau^{2}\right)$ where *τ* ^2^ is the variance due to between‐study heterogeneity (BSH), and $\epsilon_{k}$ is the random residual component with $\epsilon_{k}\sim N\left(0,\sigma_{e}^{2}\right),$ where $\sigma_{e}^{2}$ is the residual variance. The weighted heritability $\left(\mu \right)$ was calculated as:

$$\mu =\hat{\theta }=\frac{\left(\sum_{k=1}^{k}{\hat{\theta }}_{k}w_{k}^{*}\right)}{\sum_{k=1}^{k}w_{k}^{*}},$$

where $w_{k}^{*}=\frac{1}{\left(\frac{\sigma_{e}^{2}}{n}+\tau^{2}\right)}$ represents the weight attributed to the heritability estimate from the $k^{\mathrm{th}}$ study, and *n* is the total number of heritability estimates for the trait across all studies. This method ensures that higher weight is given to more accurate estimates, i.e., those with lower standard errors. The Restricted Maximum Likelihood (REML) method was used to estimate the random effects in the model, which is a suitable option for continuously distributed data (Harrer et al., 2022). The Knapp–Hartung (KH) adjustment (Knapp & Hartung, 2003) was used to calculate the standard error of $\hat{\theta }$. The KH method usually yields slightly larger confidence intervals than other methods and is especially recommended when few studies are included in the meta‐analysis for each trait, or when there is a large BSH (Harrer et al., 2022).

Four subgroup meta‐analyses were conducted as follows: (1) flight speed and flight score at weaning. In this analysis, the null hypothesis tested was that the method (speed or score) did not affect the weighted estimates of heritability for flight at weaning; (2) flight speed at weaning and flight speed at yearling. In this case, the null hypothesis tested was that the periods (weaning or yearling) did not affect the weighted estimates of heritability for flight speed; (3) pen score at weaning and pen score at yearling. The null hypothesis tested was similar to the previous one; (4) general and milking temperament. The null hypothesis tested was that the method (milking or general) did not affect the weighted estimates of heritability for temperament score in dairy cattle. In brief, all these subgroup meta‐analyses include a fixed effect in the model to obtain weighted heritability for each level of the fixed effect included. Thus, subgroup meta‐analysis enables testing specific null hypotheses and investigating potential reasons for some studies producing lower or higher results than others (Harrer et al., 2022). A model for subgroup analyses can be described as ${\hat{\theta }}_{k}=\mu +\beta x_{k}+\zeta_{k}+\epsilon_{k}$, where (${\mathrm{\beta x}}_{k}$) is the new term included in the model. The present study performed all subgroup meta‐analyses with a single categorical covariate, called meta‐regression with a categorical predictor (Harrer et al., 2022). So, *β* is the regression coefficient for the predictor X, which can have values 0 or 1 (**dummy‐coding)** in the *k* ^th^ study included in the meta‐regression.

### 2.3 Post‐meta‐analysis evaluation

The Cochran's Q test (Cochran, 1954) was used to distinguish the studies' sampling error from the BSH. This test can be calculated as $Q=\sum_{k=1}^{K}w_{k}{\left({\hat{\theta }}_{k}-\hat{\theta }\right)}^{2}$. The Q statistics follow a $\chi^{2}$ distribution with $\left(k-1\right)$ degrees of freedom, where k is the number of studies in the meta‐analysis. If Q=k−1, the differences among heritabilities from different studies are only caused by sampling error. On the contrary, if Q is significantly different from $\left(k-1\right)$, there is evidence of both BSH and sampling error affecting the differences among the reported heritabilities. In a common meta‐analysis, only one Q‐test was carried out, which tested the null hypothesis that there was no BSH. In the subgroup meta‐analysis, two Q‐tests were carried out. The first Q‐test is the same as the previous one, while the second Q‐test evaluated the null hypothesis that the weighted estimates did not differ across the levels of the fixed effect (Harrer et al., 2022). In all hypothesis tests, a significance level of 5% was used to identify significant effects.

Another metric used in meta‐analysis is the heterogeneity index $\left(I^{2}\right)$, which describes the percentage of total variation across studies that is due to heterogeneity rather than sampling error. The $I^{2}$ uses Cochran's Q value and degree freedom $\left(k-1\right)$ and can be calculated as (Higgins & Thompson, 2002): $I^{2}=\left(\frac{Q-\left(K-1\right)}{Q}\right)\times 100$. Negative values of $I^{2}$ are set to zero so that $I^{2}$ lies between 0% and 100% (Harris et al., 2008). $I^{2}$ values around 25% can be classified as low, around 50% as moderate, and around 75% as high BSH (Higgins & Thompson, 2002).

The BSH can be caused by one or more studies with extreme effect sizes (outliers), which may distort the weighted effect estimate. Moreover, it is also essential to know if the weighted effect is robust, i.e., it does not depend heavily on one influent study (Harrer et al., 2022). Therefore, the functions *find.outliers* and *InfluenceAnalysis*, both from the “dmetar” R package (Harrer et al., 2022), were used to check for outlier and influent heritability estimates, respectively. Studies are defined as outliers when their 95% CI lies outside the 95% CI of the weighted estimate. This function uses various approaches to find influent observations based on the Leave‐one‐out method (Harrer et al., 2022). In the present study, this method recalculated the results of a meta‐analysis k times (k is the number of heritability estimates), each time leaving out one heritability estimate. The results of this function were plotted in four plots as follows: (1) the Baujat plot diagnostic (Baujat et al., 2002), which shows the contribution of each study to the overall heterogeneity (as measured by the Cochran's Q test) on the horizontal axis, and its influence on the $\hat{\theta }$ value on the vertical axis; (2) the Influence Diagnostics plot, which displays eight different metrics for each study to enable the identification of the studies that fit well into the meta‐analysis model, and which ones do not; and, (3) two additional diagnostic plots showing the modification of $I^{2}$ and $\hat{\theta }$ when a study is left out.

The results from *find.outliers* and *InfluenceAnalysis* functions were used to exclude some heritability estimates from the meta‐analysis. Subsequently, the statistical Shapiro–Wilk hypothesis test was used to check if the set of heritability estimates used in each trait meta‐analysis was normally distributed, an essential assumption of a meta‐analysis (Harrer et al., 2022). If the *p*‐value was higher than 0.05, the null hypothesis was accepted, and the set of heritability estimates was considered normally distributed. Furthermore, the Funnel plot was used to evaluate publication bias (Harrer et al., 2022). In this study, a funnel plot shows the heritability estimates on the *x*‐axis in each study included in the analyses, while the *y*‐axis presents their standard errors. The funnel plots are inverted, in which higher values on the *y*‐axis represent lower standard errors. The funnel plot is a qualitative method that can be applied to any meta‐analysis and is especially useful for meta‐analysis with few estimates (Harrer et al., 2022). An asymmetry in the funnel plot is an indication of publication bias.

The results obtained in this study are presented in two parts: (1) a summary (systematic review) of all studies reporting heritability estimates for temperament in cattle, and (2) heritability estimates included in the meta‐analysis (meta‐analyses). The main meta‐analysis results, including $I^{2}$, Q‐test and its *p*‐value, weighted heritability estimate and its 95% CI, and the estimate of BSH ($\tau^{2}$) and its 95% CI were included in the forest plots with the {forest} function of the meta‐R package (https://cran.r‐project.org/web/packages/meta/meta.pdf).

## 3 RESULTS

### 3.1 Systematic review

**FIGURE 2:**
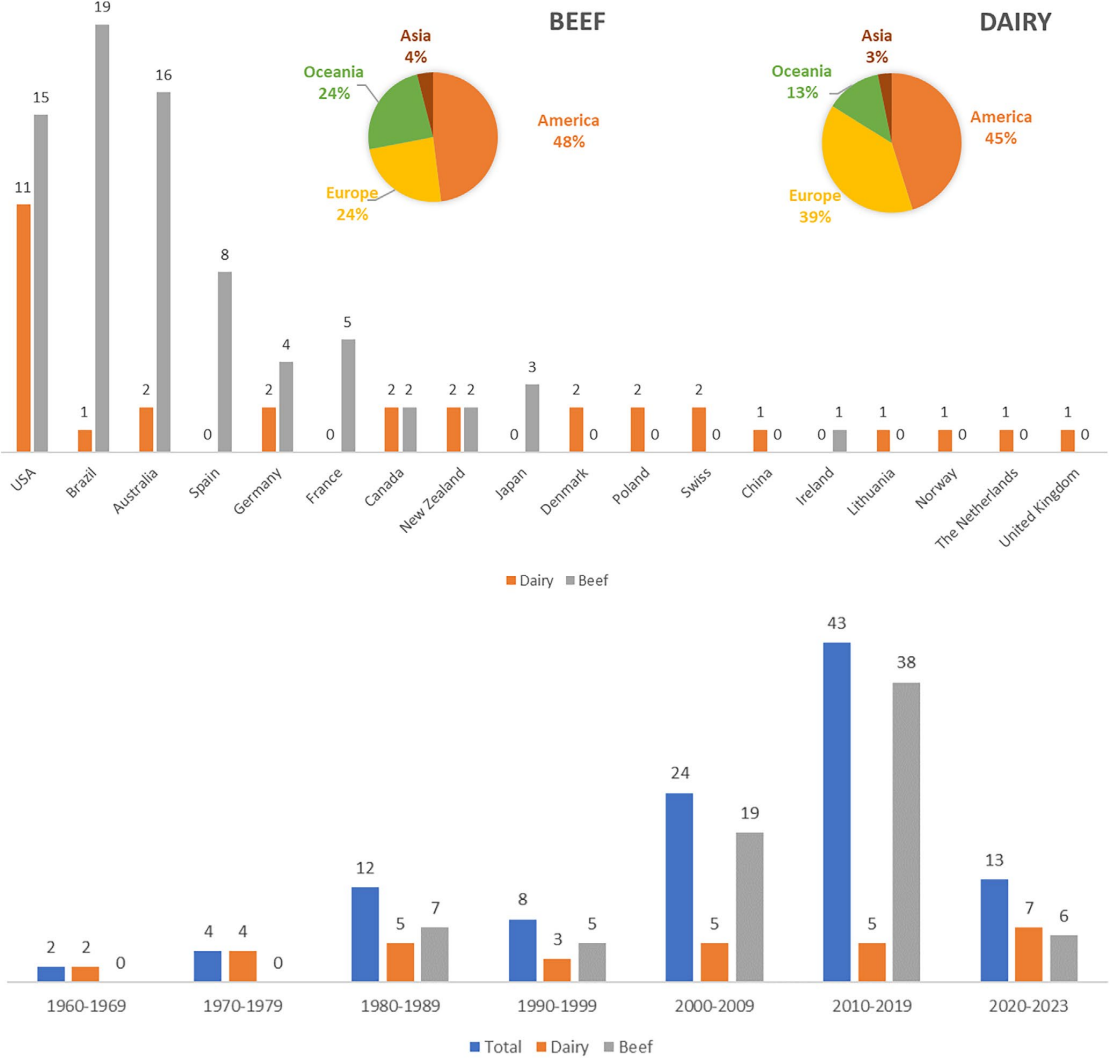
Number of studies reporting heritability estimates for temperament‐related traits in dairy and beef cattle and their countries of origin (top plot) and the distribution of the studies across the years (bottom plot). [Colour figure can be viewed at wileyonlinelibrary.com]

Based on a systematic review, we found 106 studies reporting heritability estimates for various temperament‐related traits in cattle, of which 29.2% and 70.8% were from dairy and beef cattle populations, respectively. Eleven studies were published in conference proceedings, while 95 were published in scientific journals. The studies were performed in 18 countries (Figure 2), with 24.5% from the USA, 18.9% from Brazil, 17% from Australia and 28.3% from European countries. The USA and Brazil had the highest percentage of studies with dairy (35.5%) and beef (25.3%) cattle, respectively.

For dairy cattle, 31 studies published between 1960 (O'Bleness et al., 1960) and 2023 (Taborda et al., 2023) were identified based on the systematic review, while for beef cattle, we found 75 studies published from 1981 (Sato, 1981) to 2023 (Alvarenga et al., 2023; Freitas et al., 2023; Lopez‐Carbonell et al., 2023). The distribution of the number of studies over the last six decades is shown in Figure 2. From 1960 to 1999, 53.8% of the studies reported heritability estimates for temperament‐related traits in dairy cattle, while 78.8% of the studies published from 2000 to 2023 used data from beef cattle populations.

The standard errors of the heritability estimates are valuable for meta‐analysis studies. However, 21.7% of the documents in this systematic review did not report standard errors in the estimates. This issue was higher in dairy cattle (38.7%) than in beef cattle (14.7%) studies. The 24 studies without standard error were excluded from posterior meta‐analysis studies. Moreover, 10 studies were excluded because they either did not report a detailed description of the temperament‐related traits or used the same population (or a subset of the data) reported in a previous study. A total of 72 studies (16 in dairy and 56 in beef cattle) were selected to extract all variables of interest for the meta‐analysis. Five and 67 studies were published in conference proceedings and scientific journals, respectively.

The 16 dairy cattle studies used data from cows 2 years or older from the Ayrshire, Brown Swiss, Holstein, Holstein × Gyr cross, Jersey and Norwegian Red breeds. The sample size in these studies ranged from 1212 cows (Taborda et al., 2023) to 1,940,092 cows (Sewalem et al., 2011). The heritability estimates across different traits ranged from 0.04 (Smith et al., 1985) to 0.38 (Kramer et al., 2013), while the standard error values ranged from 0.001 (Pryce et al., 2000) to 0.08 (Kramer et al., 2013). On the contrary, many studies in beef cattle analysed data from males and females of various ages, especially at weaning and yearling ages. Different pure breeds and crossbred beef cattle populations were studied. In the studies used in the meta‐analyses, the sample size ranged from 206 (Gauly et al., 2001) to 189,347 animals (Lucena et al., 2015). The heritability estimates ranged from 0.03 (Hearnshaw & Morris, 1984) to 0.61 (Gauly et al., 2001), while the standard error of the estimates ranged from 0.01 (Buddenberg et al., 1986) to 0.37 (Hearnshaw & Morris, 1984).

### 3.2 Meta‐analysis

Before the meta‐analyses, 95 heritability estimates with reported standard errors were distributed among the traits based on each trait description (Table 1). Flight score at weaning, flight speed at weaning and yearling, movement score, crush score at weaning, pen score at weaning and yearling and cow's aggressiveness at calving were evaluated mainly in beef or duo purpose breeds, and 74 heritability estimates were found for these traits. In comparison, 21 heritability estimates were found for general or milking temperament scores, which were estimated in dairy cattle populations. Statistical descriptive results for each set of heritability and the *p*‐value of the Shapiro–Wilk test are presented in Table 2.

**TABLE 1:** Summary of each temperament‐related trait in dairy (two traits) and beef (six traits) cattle.

Dairy cattle traits^a^	
Milking temperament	Cow's temperament is assessed during milking. The scoring grid ranges between studies. As an example, Sewalem et al. (2011) reported a score system defined as: (1) very nervous, (2) nervous, (3) average temperament, (4) calm, (5) very calm.
General temperament	Cow's temperament is assessed as an overall score within the herd environment, not only during milking. For instance, Kramer et al. (2013) reported a scoring system ranging from (1) very nervous to (5) very calm.
Beef cattle traits^a^	
Flight score	Animal's temperament is assessed as it leaves the scale/chute immediately after handling. For example, Hoppe et al. (2010) reported a scoring system defined as (1) walking, (2) trotting, (3) running, and (4) jumping out of the chute.
Flight speed	Speed or time taken for an animal to cover a distance after leaving a scale or chute (Burrow et al., 1988). Generally, the distance ranges from 1.5 to 3.0 m, depending on the farm facilities. An electronic device records the time to move out of a constraint environment, which is later converted to speed (m/s).
Crush score	This visual score is assigned while the animal is restrained in a chute with headgates. As an example, Hearnshaw and Morris (1984) reported a scoring grid with the following categories: (0) stands very quietly, offers no resistance, only casual tail swishing; (1) generally quiet, offers token resistance, steady movement in bail; (2) slightly excited movement, straining and paddling, may kick; (3) excited, vigorous abrupt movement, straining and paddling, may kick; (4) very disturbed, frightened, wild movements, many jumps and falls down in crush; and, (5) unmanageable and dangerous.
Movement score	This score is assigned while the animal is restrained in a chute without the head restrained by headgates. Sant'Anna et al. (2015) reported a grid example as: (1) no movement, (2) little movement during less than half of the observation time, (3) frequent movements (during half of the observation time or more), but not vigorous, (4) constant and vigorous movements, (5) constant and vigorous movements, the animal jumps and raises its forelimbs off the ground.
Pen score	The animal is removed from a large pen where their herdmates are to a smaller pen, and the animal's reactivity to this handling is evaluated. Moreover, its reactivity to human presence is usually evaluated while in the small pen, too. For example, Gauly et al. (2001) reported a scoring system as follows: (1) calm, (2) slightly nervous, (3) nervous, (4) excited, (5) very excited.
Cow's aggressiveness at calving	This score evaluates the cow's aggressiveness towards the handler when it handles her newborn calf, generally in the first 24 h after calving. For example, Morris et al. (1994) reported a scoring system as (0) cow stands quietly and may occasionally lick the calf, (1) generally quiet, offers token resistance to the calf being handled, may observe from a distance (>6 m), but returns after the operations are completed, (2) slightly excited, occasionally pawing the ground, (3) excited, vigorous, tail‐swishing, bellows loudly when the calf is handled, (4) very disturbed, the cows attempts to interfere with the tagging operation, and the operator only feels safe if the cow is watched at all times, and he/she may need protection from a second handler, (5) the cow is dangerous and unmanageable, continually pushing the handler away from her calf.

^a^ The grid score values range across studies, and the scoring systems presented in this table are only for example purposes.

**TABLE 2:** Descriptive analysis of the set of heritability (h^2^) estimates for each trait and the *p*‐value of the Shapiro–Wilk test.

Trait	Number of studies	Number of animals^a^	Min. Sample size	Max. Sample size	Min.	Max.	Average h^2^ ± standard error	*p*‐Value
Flight score at weaning	6	5228	424	2178	0.11	0.36	0.23 ± 0.03	0.9785
Flight speed at weaning	9	17,690	494	4645	0.12	0.54	0.29 ± 0.04	0.1128
Flight speed at yearling	10	47,500	302	16,801	0.17	0.49	0.28 ± 0.03	0.1342
Movement score	11	41,501	250	16,874	0.08	0.29	0.17 ± 0.02	0.0607
Crush score at weaning	15	163,100	350	50,935	0.03	0.46	0.24 ± 0.03	0.4005
Pen score at weaning	9	13,694	206	6132	0.14	0.61	0.37 ± 0.06	0.5407
Pen score at yearling	9	293,926	250	189,347	0.11	0.29	0.19 ± 0.02	0.7162
Cow's aggressiveness at calving	5	11,991	578	5881	0.06	0.42	0.16 ± 0.06	0.1889
Milking temperament	11	3,752,645	1212	1,940,092	0.04	0.33	0.15 ± 0.03	0.3173
General temperament	10	222,484	2312	126,614	0.04	0.38	0.14 ± 0.03	0.0585

^a^ It is the sum of animals from all studies.

Firstly, meta‐analyses were carried out using all identified studies that reported SE and a detailed definition of the traits of interest. The results from the initial meta‐analyses were used to check for outliers, defined as heritability estimates with 95% CI outside of the range (95% CI) of the weighted estimate. For instance, Figure S1 shows the weighted heritability of 0.26 (95% CI: 0.20–0.33) estimated by the initial meta‐analysis performed for the pen score trait; while the heritability estimates reported by Riley et al. (2014) and Schmidt et al. (2014) were 0.47 (95% CI: 0.34–0.60) and 0.49 (95% CI: 0.37–0.61), respectively. As the 95% CI of these two heritability estimates did not overlap the 95% CI of the weighted estimate, both were identified as outliers. The initial meta‐analyses performed for crush score (Figure S2), cow's aggressiveness at calving (Figure S3), and the general and milking temperament scores (Figure S4) also estimated weighted heritability with 95% CI that allowed identifying as outliers the following estimates: 0.38 ± 0.03 (Beckman et al., 2007) and 0.46 ± 0.03 (Walkom et al., 2018) for crush score at weaning; 0.42 ± 0.05 (Hoppe et al., 2008) reported for cow's aggressiveness; 0.33 ± 0.06 (Cue et al., 1996), 0.09 ± 0.006 (Wethal et al., 2020), 0.07 ± 0.01 (Oliveira‐Junior et al., 2021), and 0.09 ± 0.0012 (Szymik et al., 2021) for general temperament; and 0.04 ± 0.02 (Smith et al., 1985), 0.07 ± 0.001 (Pryce et al., 2000), and 0.38 ± 0.07 (Kramer et al., 2013) for milking temperament.

The results from the first meta‐analysis were also used in the analyses of influential factors, which were performed to identify the effects of each heritability estimate on the weighted heritability estimate and BSH. The Baujat plot (Figures S5–S11) indicates the influence of each study on the weighted heritability estimate (vertical axis) and the BSH (horizontal axis). Thus, studies located in the bottom left quadrant usually do not have a significant influence on both weighted heritability and BSH. On the other hand, studies at the top right quadrant may have a high impact on both the weighted estimate and BSH. Studies at the bottom‐right and top‐left quadrants may strongly influence BSH and weighted heritability, respectively. The Baujat plot analysis is a subjective approach, as the influence of the studies depends on how high the values on the vertical and horizontal axes are. In Figures S5–S7, there were some isolated studies in quadrants that indicate influence, but the values on the *x*‐ and *y*‐axes are small, suggesting that there is no evidence of a large impact of any study on the weighted heritability estimates or a high contribution to BSH. On the other hand, some estimates were found with a high contribution for BSH, such as 0.46 ± 0.03 (Walkom et al., 2018) for crush score at weaning (Figure S8), 0.47 ± 0.07 (Riley et al., 2014), and 0.49 ± 0.06 (Schmidt et al., 2014) for pen score (Figure S9), which did not have a large influence on the weighted heritability estimate. An interesting example can be observed for cow's aggressiveness, where the estimates 0.42 ± 0.05 (Hoppe et al., 2008) and 0.06 ± 0.01 (Buddenberg et al., 1986) had a high and low contribution to the BHS, respectively. These same estimates showed low and high influence on the weighted heritability estimate, respectively (Figure S10). The estimates 0.07 ± 0.01 (Pryce et al., 2000) and 0.09 ± 0.01 (Szymik et al., 2021) had high contributions to the BSH and weighted heritability estimates found in the subgroup meta‐analysis of general and milking temperament traits (Figure S11).

A second subjective influence analysis was performed using diagnostic plots (Figures S12–S18). The studies appear on the horizontal axis, and different metrics are shown on the vertical axis. No red peaks were observed in (Figures S12 and S13), suggesting that there were no studies influencing multiple metrics in the diagnostic plot in these meta‐analyses. On the other hand, red peaks identified the following estimates as influents: 0.08 ± 0.03 (Freitas et al., 2023), 0.10 ± 0.03 (Sant'Anna et al., 2015), and 0.11 ± 0.03 (Valente et al., 2015) (Figure S14), 0.46 ± 0.03 (Walkom et al., 2018) (Figure S15), 0.49 ± 0.06 (Schmidt et al., 2014) (Figure S16), and 0.42 ± 0.05 (Hoppe et al., 2008) (Figure S17). Although red points were not observed in the subgroup meta‐analysis of general and milking temperament traits (Figure S18), there are peaks in some metrics for the estimates of Cue et al. (1996), Kramer et al. (2013), Pryce et al. (2000), and Szymik et al. (2021).

A leaving‐one‐out analysis was also used to identify influential studies (Figures S19–S25). These figures show the modification in weighted heritability and $I^{2}$ values when one specific study is removed from the meta‐analysis. Figures S19 and S20 show that removing the estimate 0.21 ± 0.02 (Kadel et al., 2006) and 0.21 ± 0.02 (Valente et al., 2016) could reduce $I^{2}$, but the BSH would remain moderate. Figure S21 also showed some reduction in the $I^{2}$, but with small practical effects on the BSH, which was small in the meta‐analysis. Expressive reduction in the $I^{2}$ values were observed when the following estimates (also outliers) were removed from the meta‐analyses: 0.46 ± 0.03 (Walkom et al., 2018) (Figure S22), 0.49 ± 0.06 (Schmidt et al., 2014) and 0.47 ± 0.07 (Riley et al., 2014) (Figure S23). Leaving‐one‐out analysis also showed a large reduction in both $I^{2}$ and weighted heritability for cow's aggressiveness at calving by removing the 0.42 ± 0.05 estimate (Hoppe et al., 2008) (Figure S24). No large modification was observed for milking and general temperament score traits (Figure S25).

**FIGURE 3:**
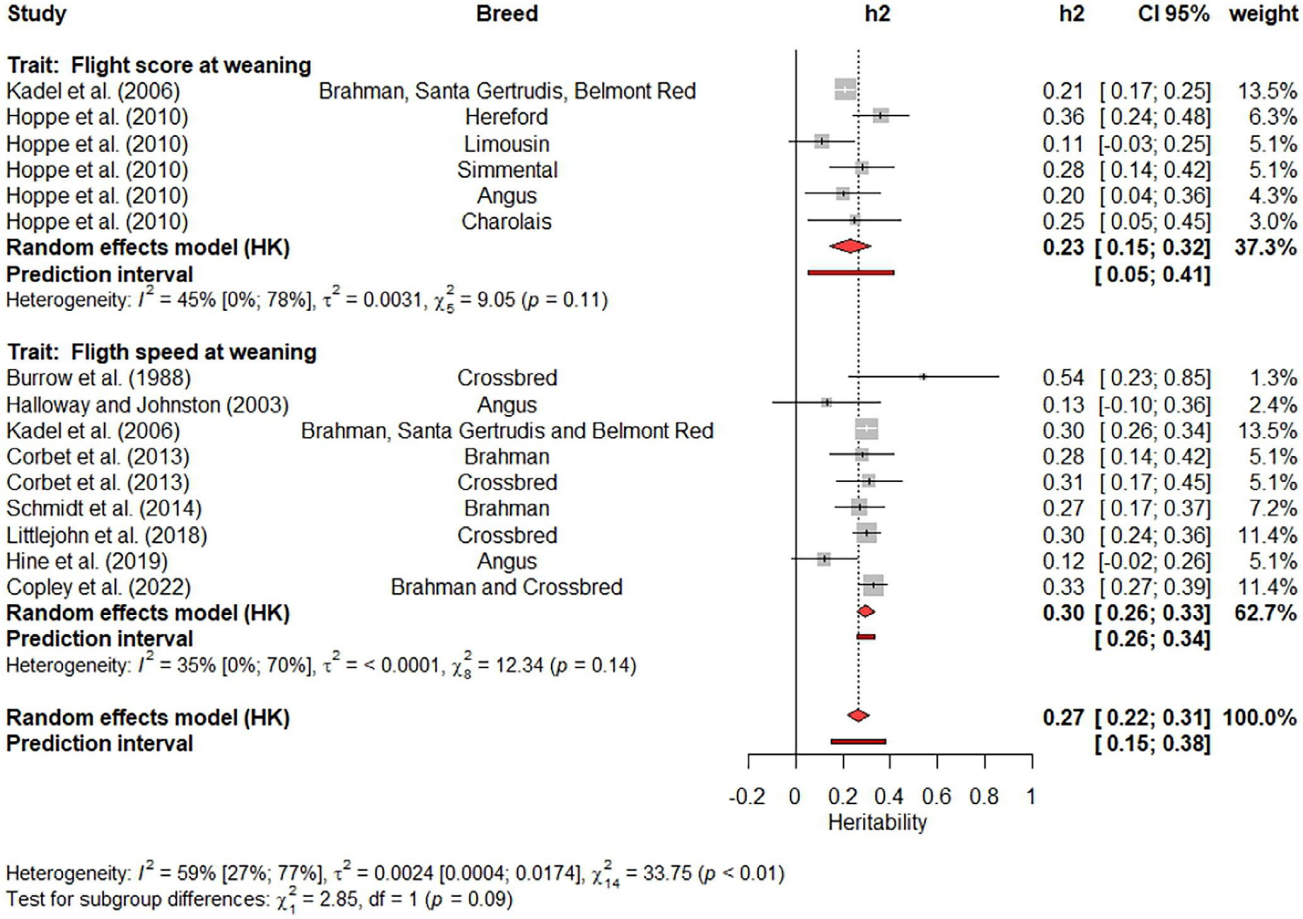
Subgroup meta‐analysis of heritability estimates for flight speed and flight score at weaning in beef cattle populations. [Colour figure can be viewed at wileyonlinelibrary.com]

**FIGURE 4:**
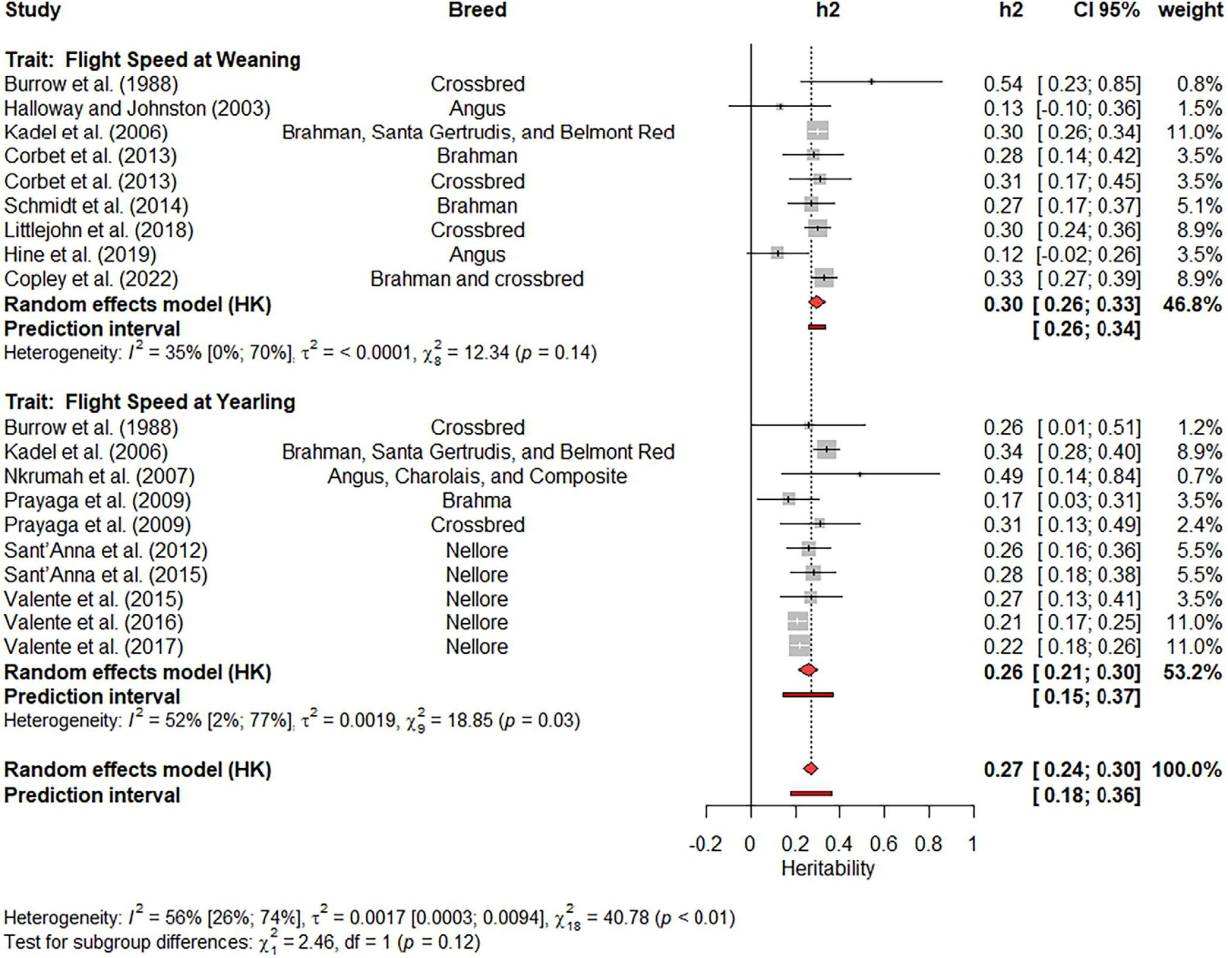
Subgroup meta‐analysis of heritability estimates for flight speed at weaning and at yearling in beef cattle populations. [Colour figure can be viewed at wileyonlinelibrary.com]

**FIGURE 5:**
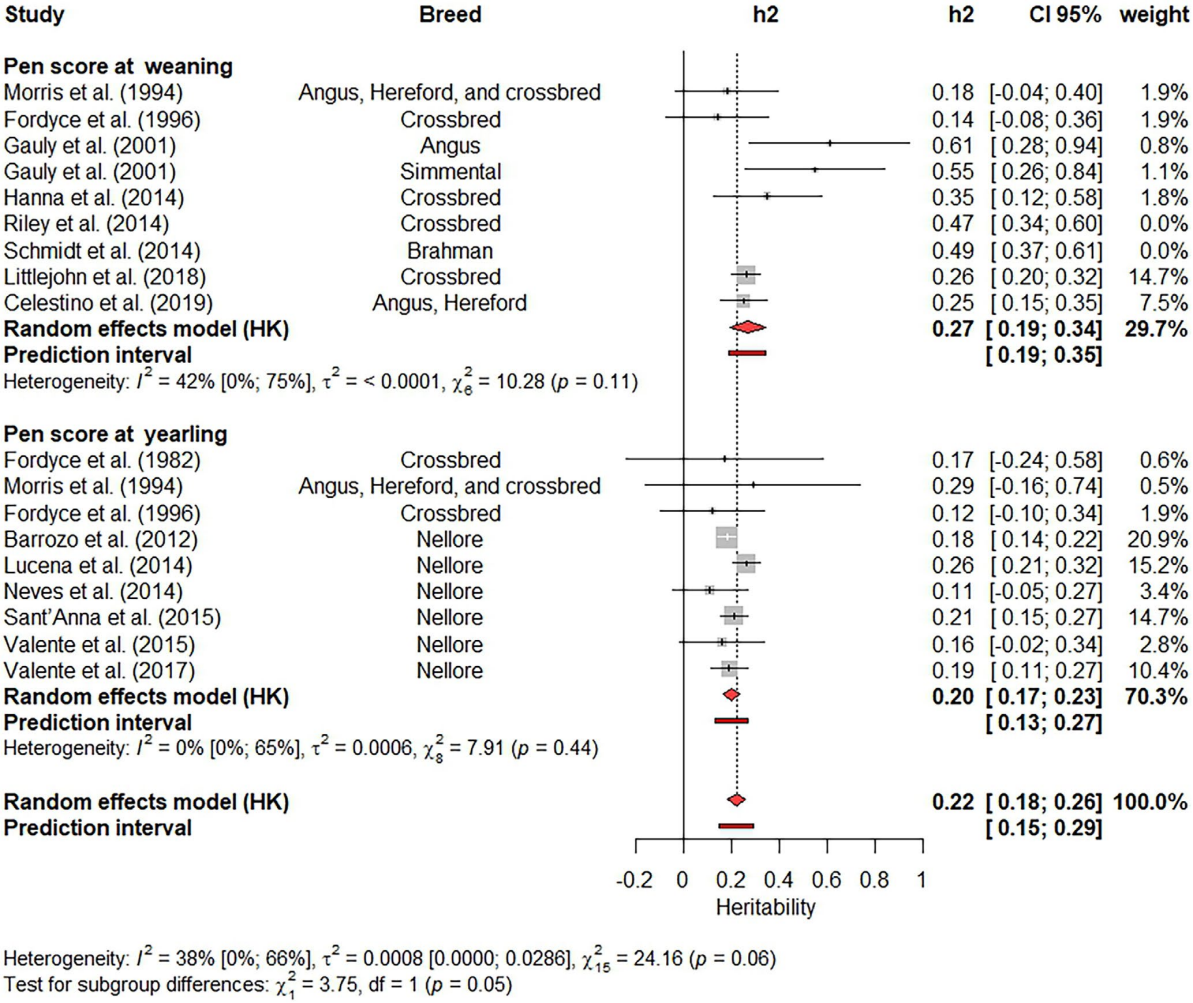
Meta‐analysis of heritability estimates for pen score (measured at weaning and yearling) in beef cattle populations. [Colour figure can be viewed at wileyonlinelibrary.com]

**FIGURE 6:**
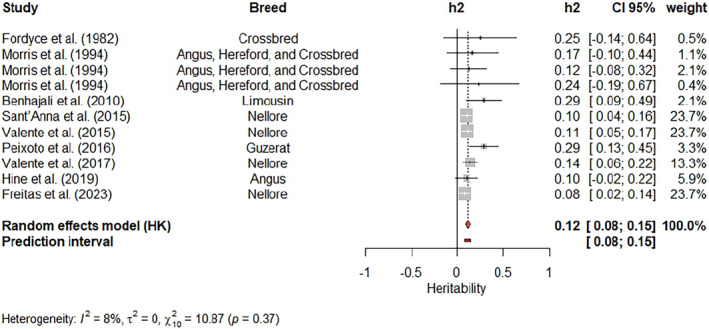
Meta‐analysis of heritability estimates for movement score in beef cattle populations. [Colour figure can be viewed at wileyonlinelibrary.com]

**FIGURE 7:**
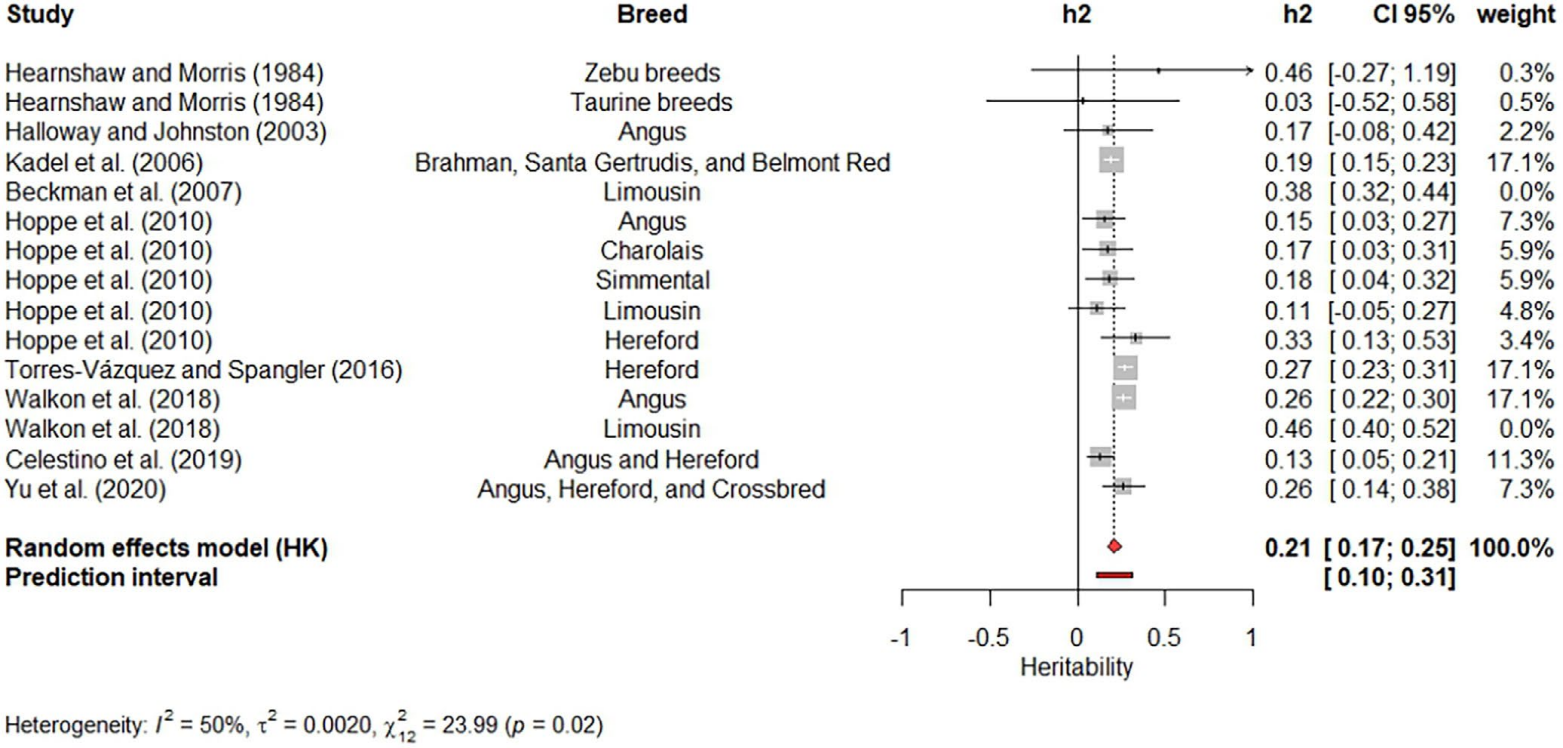
Meta‐analysis of heritability estimates for crush score at weaning in beef cattle populations. [Colour figure can be viewed at wileyonlinelibrary.com]

**FIGURE 8:**
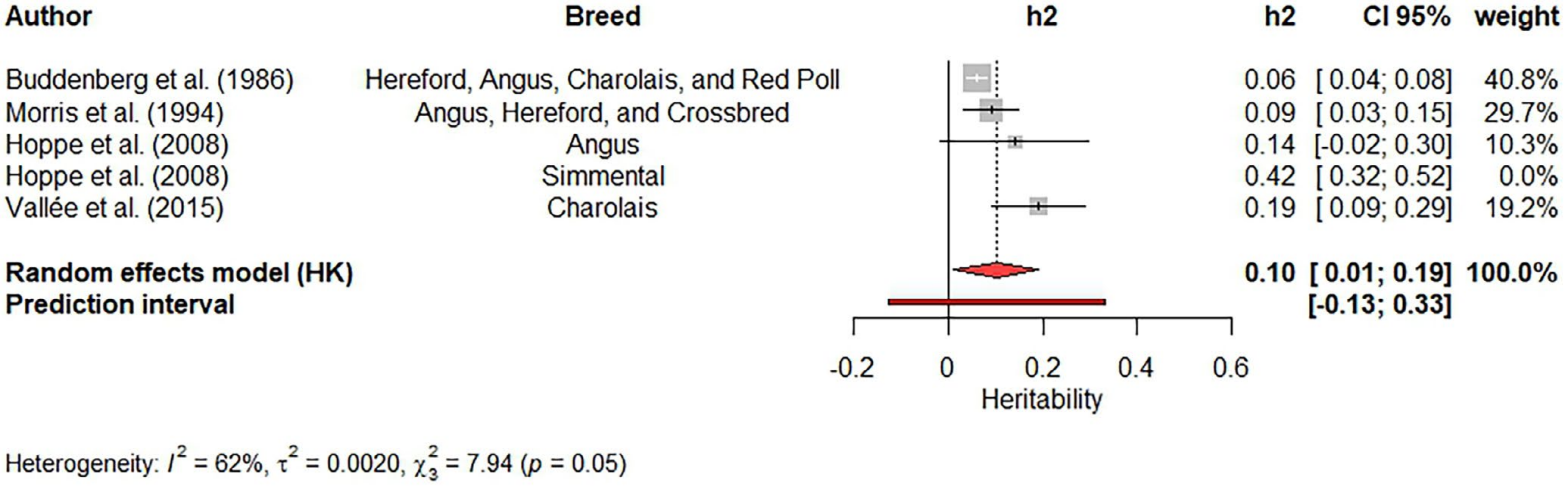
Meta‐analysis of heritability estimates for cow's aggressiveness at calving in beef cattle populations. [Colour figure can be viewed at wileyonlinelibrary.com]

**FIGURE 9:**
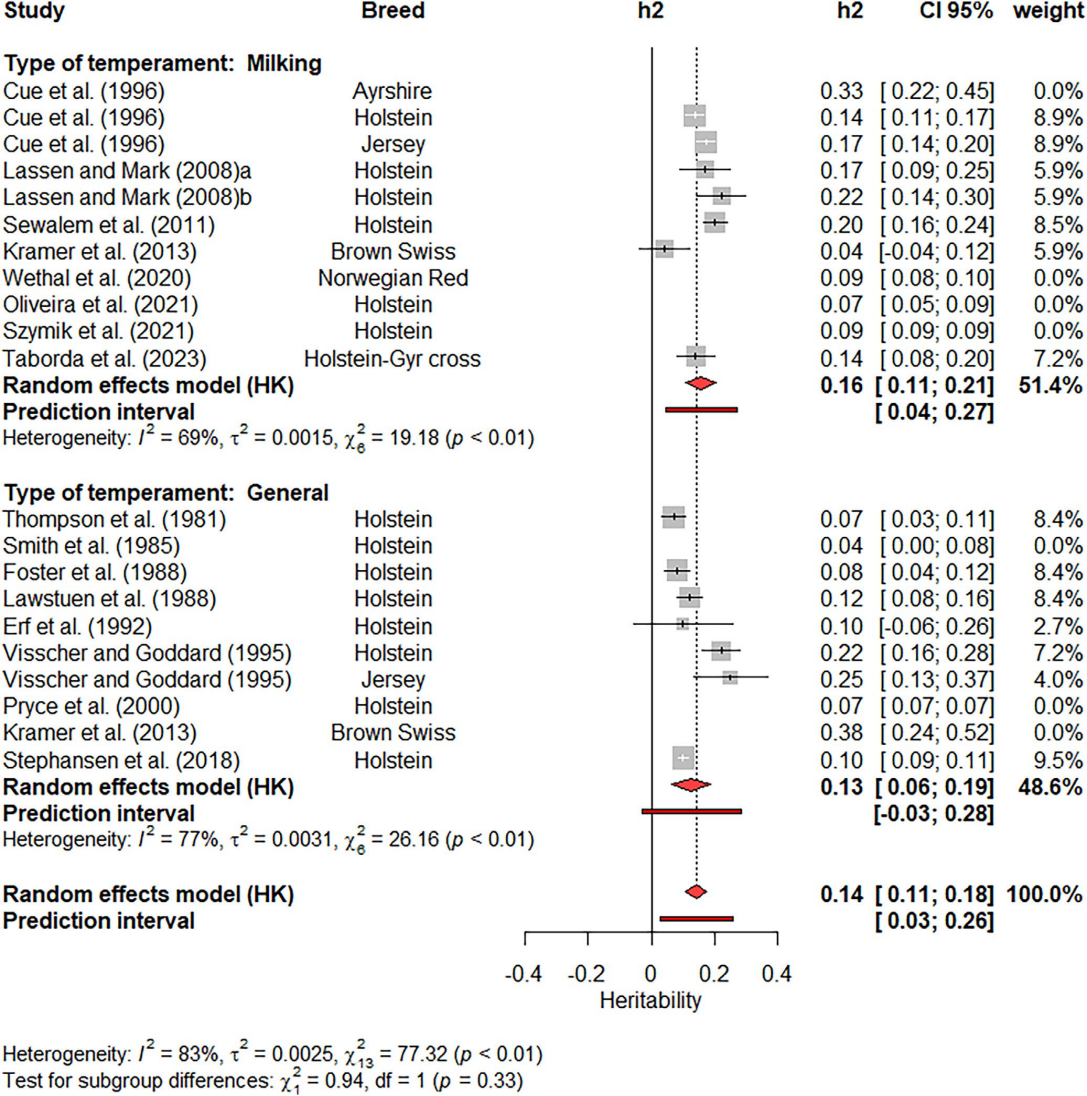
Subgroup meta‐analysis of heritability estimates for general and milking temperament in dairy cattle populations. [Colour figure can be viewed at wileyonlinelibrary.com]

It is difficult to exclude estimates from meta‐analysis based on subjective analyses. In the present study, only the estimates identified as outliers were removed from subsequent analyses, many of which had high contributions to the BSH and a high influence on the weighted estimate. Outliers were excluded by updating the results from the previous meta‐analysis using a function called ‘update’ from the Meta‐R package. This function performs a new meta‐analysis after attributing a weight of zero to all studies indicated by the users. After this update of the results, Forest plots (Figures 3, 4, 5, 6, 7, 8, 9) were prepared to show the final results of the meta‐analyses. Forest plots are useful for showing the results of meta‐analyses, as they also present the heritability estimates reported in previous studies, enabling a clear visualization of the differences across studies. Each Forest plot shows the main author of each study, the breeds included in each study, the heritability illustration (a box with a line crossing it, where the box size is inversely proportional to the SE value and the line length indicates the 95% CI), the heritability estimates and their 95% CI, and the weight attributed for each estimate when calculating the weighted heritability estimate. Furthermore, a graphical representation of the weighted estimate is symbolized by a red diamond, where the horizontal extremities of the diamond delimit its 95% CI. Numeric values of the weighted estimates and their 95% CI are presented in bold. Finally, $I^{2}$ with its 95% CI, the variance caused by BSH $\left(\tau^{2}\right)$, and the *p*‐values for the Q‐tests are also shown in each Forest plot. It is worth highlighting that the removed outlier studies remain in the Forest plot but with no central box, and a weight of 0% is assigned to them in the column “weight.”

The heritability estimates for flight speed and flight score at weaning were included in a subgroup meta‐analysis, where the measurement method (speed or score) was used as a fixed effect. No differences (*p*‐value = 0.09) between the methods were identified based on the Q‐test for subgroup differences (Figure 3). The weighted estimates ranged from 0.23 (flight score at weaning) to 0.30 (for flight speed at weaning), and for both traits, the heritability reported by Kadel et al. (2006) had the highest weights (13.5%). Other estimates with relatively large contributions (11.4%) were reported by Littlejohn et al. (2018) and Copley et al. (2022). The $I^{2}$ values ranged from 35% to 59%, and their 95% CI overlapped, while the Q‐test for BSH was significant for the joint analysis (*p* < 0.01) but not significant (*p* > 0.05) for each single trait meta‐analysis.

Two subgroup meta‐analyses were applied to test the effect of the class of age (weaning or yearling) on flight speed and pen score, where the *p*‐values were 0.12 (Figure 4) and 0.05, respectively (Figure 5), indicating a non‐significant difference in flight speed. The weighted heritability estimates were moderate for both traits, with values ranging from 0.26 (flight score at weaning) to 0.30 (for flight speed at weaning) and from 0.20 (pen score at yearling) to 0.27 (pen score at weaning). The estimates reported by Kadel et al. (2006), Valente et al. (2016), and Valente et al. (2017) showed the highest weight (11%) for flight speed analysis, while Barrozo et al. (2012), Lucena et al. (2015), Sant'Anna et al. (2015) and Littlejohn et al. (2018) reported the estimates with higher contribution to the pen score weighted estimate. The $I^{2}$ ranged from 35% to 56% for flight speed and from 0% to 42% for pen score. The Q‐test for BSH was significant for both flight speed at yearling (*p* = 0.03) and joint analysis of flight speed at weaning and yearling (*p* < 0.01). This test was not significant (*p* > 0.05) for the pen score meta‐analyses.

Single meta‐analyses were carried out for movement score (Figure 6), crush score at weaning (Figure 7) and cow's aggressiveness at calving (Figure 8). The weighted heritability estimates were 0.12 (movement score), 0.10 (cow's aggressiveness) and 0.20 (crush score). The estimates reported by Sant'Anna et al. (2015), Valente et al. (2015) and Freitas et al. (2023) had the highest weights (23.7%) for the weighted heritability estimated for movement score. Kadel et al. (2006), Torres‐Vázquez & Spangler (2016) and Walkom et al. (2018) reported heritability estimates with the highest contribution (17.1%) to the weighted heritability for crush score, while Buddenberg et al. (1986)'s estimate had the highest weight (40.8%) in the meta‐analysis for cow's aggressiveness at calving. A low value of $I^{2}$, with no significant Q‐test (*p* > 0.05), was found for movement score. On the other hand, moderate $I^{2}$ values and significant Q‐tests (*p* < 0.05) were found for both crush score and cow's aggressiveness at calving.

A subgroup meta‐analysis was applied for the two dairy cattle traits (Figure 9), where the measurement approach (general or milking) was included in the model as a fixed effect. The Q‐test for subgroup differences was not significant (*p*‐value = 0.33). The weighted heritability estimates ranged from 0.13 (general temperament) to 0.16 (milking temperament). The weights of the studies ranged from 2.7% (Erf et al., 1992) to 9.5% (Stephansen et al., 2018). High $I^{2}$ values and highly significant Q‐tests (*p* < 0.01) for BSH were found for these traits in both single and join meta‐analyses.

The possibility of publication bias was investigated using the funnel plots (Figures S26–S32). These plots show the distribution of studies depending on the heritability estimate value (*x*‐axis) and standard error (*y*‐axis). It should be noted that the *y*‐axis is inverted; that is, the lowest SE values are at the top of the *y*‐axis. Funnel plot analysis assumes that if two studies have heritability with high‐standard errors, the one with higher heritability will have a greater chance of being accepted for publication. Thus, no publication bias is supported by a symmetrical distribution of estimates across the central line of the funnel. For instance, Figure S26 shows the estimate of 0.54 ± 0.16 (Burrow et al., 1988) on the right‐bottom side of the funnel. However, no small heritability was reported with a large SE, i.e., on the left‐bottom side, suggesting a potential publication bias for the subgroup meta‐analyses (flight speed × flight score). Potential publication bias was also observed for the subgroup meta‐analysis of flight speed at weaning and yearling (Figure S27) and crush score at weaning (Figure S29). Publication bias was not found by graphical funnel plot analyses of other meta‐analysis (Figures S28, S30–S32).

## 4 DISCUSSION

### 4.1 Systematic review

Previous reviews showed the contribution of the additive genetic variance for temperament‐related trait variation in cattle and found a high amplitude of heritability estimates for many traits (Chang et al., 2020; Haskell et al., 2014), but these reviews did not include a meta‐analysis of the heritability estimates. A previous meta‐analysis reported a moderate weighted heritability (0.24 ± 0.15) for flight speed in beef cattle (Gathura et al., 2020) but did not analyse other temperament‐related traits. Therefore, the present study was the first to perform a specific systematic review to estimate weighted heritability for various temperament‐related traits in cattle. The systematic review showed that at least 106 studies have been published in the last 63 years, which reported many heritability estimates for different temperament‐related traits in cattle. The studies were carried out in at least 18 countries. However, the USA, Brazil and Australia were the countries with the largest number of studies (Figure 2). In 2022, these three countries had 350.9 million cattle (www.fao.org/faostat/en/#data/QCL) and various breeding programs, which have generated databases for several traits, including indicators of temperament. The small number of countries represented and reduced the distribution of studies in continents such as Africa and Asia indicate that heritability estimates in cattle populations raised across different environmental and management conditions remain unknown.

Another important issue reported in this systematic review is the concentration of studies on some cattle breeds. For instance, 68% of the studies performed on dairy cattle used data from Holstein (*Bos taurus taurus*) populations. Although there is greater diversification in beef cattle, many studies were observed for a few breeds. For instance, 70% of the studies performed in Brazil used data from the Nellore breed (*Bos taurus indicus*), but there are many other Taurine and Zebuine beef cattle and composite breeds in Brazil. Therefore, heritability for temperament‐related traits remains unknown (or poorly studied) in many cattle breeds, especially in Zebu cattle breeds, which are mainly reported in Brazilian studies. This makes the weighted estimates reported in the present meta‐analysis even more important, as they can serve as initial (temporary) estimates for breeding programs performing genetic evaluations for temperament traits.

Although some breeds have yet to be studied, our systematic review shows that genetics of cattle temperament has become an area of great interest in recent years. In the 1960s and 1970s, we found only dairy cattle studies, especially those done with Holstein breed datasets (Aitchison et al., 1972; Dickson et al., 1970; Norman & Van‐Vleck, 1971; O'Bleness et al., 1960; Van‐Vleck, 1964; Wickham, 1979). Between the 1980s and 1990s, there was a more balanced number of studies on dairy (Agyemang et al., 1982; Cue et al., 1996; Erf et al., 1992; Foster et al., 1988; Lawstuen et al., 1988; Smith et al., 1985; Thompson et al., 1981; Visscher & Goddard, 1995) and beef (Buddenberg et al., 1986; Burrow et al., 1988; Fordyce et al., 1982, 1996; Fordyce & Goddard, 1984; Hearnshaw & Morris, 1984; Le Neindre et al., 1995; Morris et al., 1994; Mourão et al., 1998; Oikawa et al., 1988; Sato, 1981) populations. However, in the last 20 years, most studies have used beef cattle datasets (Figure 2). The larger number of beef cattle breeds raised worldwide than dairy cattle may partially explain this difference. Handling frequency is another factor that has led to more studies on beef cattle. Milking is a daily activity in dairy herds, which contributes to improving farm‐staff × animal interaction. On the contrary, handling activities in beef cattle are less frequent, which makes the farm‐staff × animal interaction more challenging when they happen.

Based on the systematic review, we identified various heritability estimates reported for many temperament‐related traits, using many breeds from different countries and production systems. Unfortunately, some specific temperament‐related traits found in our systematic review were not included in the meta‐analysis because insufficient heritability estimates were reported to calculate weighted heritability estimates for these traits. For instance, few heritability estimates have been reported for milking failures and refusals in automatic milking systems (Pedrosa et al., 2023). When more studies with this trait are carried out, it will be possible to perform a meta‐analysis to obtain a weighted estimate. Furthermore, different studies reported heritability estimates using data from the same population (e.g., Kramer et al., 2013, 2014), and the meta‐analysis assumes that the estimates are independent. In this case, only one of the studies was used in the meta‐analysis, and to define duplicate studies, factors such as the sample size and the origin of the data were analysed.

Most studies with temperament traits used small samples, especially in beef cattle populations. Even in the dairy cattle studies, where there is a large difference between the biggest and smallest studies, we did not observe a clear relationship between the year of the study and the sample size. For instance, the largest study on milking temperament used Holstein breed in Canada, where almost 2.0 million cows were evaluated (Sewalem et al., 2011), but 2 years later, a much smaller study (*N* = 2259 cows) was carried out with Holstein in Swiss (Kramer et al., 2013). The same scenario has been repeated more recently, as Szymik et al. (2021) and Taborda et al. (2023) published studies with one million and 1212 cows, respectively. Even if there was a tendency to increase sample size across time, as observed with some classical traits in the animal breeding area, this would not be an issue because estimates with larger standard errors (small sample) have minor weights in the meta‐analyses, preventing these estimates from having any major impact on the weighted heritability.

Another important point to be highlighted is the type of temperament traits measured in beef and dairy cattle population for breeding purposes. In dairy cattle, the classical measurements are milking and general temperament scores, while flight speed, crush score, movement score and pen score are the main scores assessed in beef cattle. The traits traditionally used to evaluate dairy cattle do not apply to beef cattle, as they depend on cows in a dairy farming context (Chang et al., 2020; Haskell et al., 2014). On the other hand, beef cattle traits could be used in dairy cattle evaluation, but there are no heritability estimates for these traits in dairy cattle, which prevents a meta‐analysis. Although heritability was not estimated, Gibbons et al. (2011) showed that flight speed provides a consistent temperament assessment in dairy cows. Moreover, as our meta‐analysis will show below, flight speed is a key selection criterion in beef cattle, while the traditional traits used in dairy cattle have high BSH. However, future studies are needed to evaluate if temperament‐related traits commonly used in beef cattle can also be an alternative for assessing the genetic control of temperament in dairy cattle.

### 4.2 Flight speed and flight score in beef cattle

Flight speed and flight score are two traits often used in beef cattle breeding programs, and the systematic review found 25 heritability estimates for these traits after data editing. Both traits assess the animal's behaviour when it leaves a scale or chute after handling. The reported estimates of heritability ranged from 0.12 (Hine et al., 2019) to 0.54 (Burrow et al., 1988) for flight speed at weaning and from 0.11 to 0.36 (Hoppe et al., 2010) for flight score at weaning. Therefore, both traits are under genetic control. Although there is a greater number of studies reporting heritability estimates for flight speed at weaning (Burrow et al., 1988; Copley et al., 2022; Corbet et al., 2013; Halloway & Johnston, 2003; Hine et al., 2019; Kadel et al., 2006; Littlejohn et al., 2018; Schmidt et al., 2014) than flight score at weaning (Hoppe et al., 2010; Kadel et al., 2006), a slightly lower $I^{2}$ was observed for flight speed (35%) than flight score (45%) (Figure 3). However, the 95% CI of these $I^{2}$ values overlapped, suggesting a non‐significant difference between them. It must be noted that flight score is a subjective trait, and the scores depend on how the animal leaves the chute or scale (Hoppe et al., 2010). On the other hand, flight speed is an objective trait that requires an electronic device to measure the time taken for an animal to leave the scale or chute and move through a fixed distance (Burrow et al., 1988). Although moderate $I^{2}$ values have been found for these traits in our meta‐analysis, the Q‐test was not significant for both traits, which suggests low heterogeneity across studies.

The Q‐test for BSH was significant (*p* < 0.01) in the joint analysis, which was not observed in single analyses of flight speed and flight score at weaning. This result indicates that the studies are homogeneous within each trait and heterogeneous across traits. This result also suggests that the heritability estimates for flight may depend on the method used to record it. Kadel et al. (2006) used both methods and found a higher heritability estimate for flight speed (0.30) than flight score (0.21). The Q‐test for subgroup differences found no significant differences in the weighted heritability estimates for flight speed and score (*p* = 0.09), which can result from reduced testing power due to the reduced number of estimates in this subgroup meta‐analysis. It should be noted that the prediction intervals for flight speed (0.26–0.34) and flight score (0.05–0.41) at weaning differed. The wide prediction interval of the flight score is a consequence of the high standard error values (between 0.06 and 0.10) of the heritabilities reported by Hoppe et al. (2010), which used between 424 and 706 animals to estimate variance components. Kadel et al. (2006) used 2178 animals and reported a standard error 0.02. Therefore, more flight score at weaning studies with larger sample sizes are needed to estimate a more reliable weighted heritability for this trait using meta‐analysis. The prediction interval for flight speed at weaning suggests that this trait has a moderate heritability. It should be noted that of the nine heritability estimates reported for flight speed at weaning, only Halloway and Johnston (2003) (0.13 ± 0.12) and Hine et al. (2019) (0.12 ± 0.07) reported low estimates, while the other seven estimates ranged from 0.27 ± 0.05 (Schmidt et al., 2014) to 0.54 ± 0.16 (Burrow et al., 1988).

Unlike flight speed at weaning, flight speed at yearling had a significant Q‐test for BSH (*p* = 0.03), suggesting that recording animals at yearling produces greater BSH than at weaning. The estimates of heritability reported for flight speed also allowed us to test the class of measurement age (weaning and yearling) as a fixed effect for this trait, which was not significant (*p*‐value = 0.12). This is another important result found in the present study because it suggests that breeding programs can use an earlier flight speed record (when the calf was being weaned), which usually has more animals for evaluation at weaning than yearling due to sell of animals by the farmers after weaning. Moreover, an earlier record reduces the effect of handling routine on an animal's temperament because an animal's previous experience with handling may affect its reaction to handling in the future (Grandin, 1989). However, the prediction intervals at weaning (0.26–0.34) and yearling (0.15–0.37) differed slightly. Although more studies have been carried out at yearling (Burrow et al., 1988; Kadel et al., 2006; Nkrumah et al., 2007; Prayaga et al., 2009; Sant'Anna et al., 2012, 2015; Valente et al., 2015, 2016, 2017) than at weaning (Burrow et al., 1988; Copley et al., 2022; Corbet et al., 2013; Halloway & Johnston, 2003; Hine et al., 2019; Kadel et al., 2006; Littlejohn et al., 2018; Schmidt et al., 2014), the prediction intervals suggest a more reliable weighted heritability at weaning than at yearling. In other words, the meta‐analysis indicates that the heritability of flight speed at weaning is moderate (95% CI: 0.26–0.34) and may be low or moderate at yearling (95% CI: 0.15–0.37).

### 4.3 Movement score and crush score in beef cattle

For movement score evaluation, the observer must check the quantity and intensity of an animal's movement while handled inside the chute or scale during a time interval. Our systematic review found estimates for movement score at weaning (Benhajali et al., 2010; Hine et al., 2019; Morris et al., 1994), yearling (Fordyce et al., 1982; Morris et al., 1994; Sant'Anna et al., 2015; Valente et al., 2015, 2017), and using several ages (Freitas et al., 2023; Morris et al., 1994; Peixoto et al., 2016). Even with these different classes of age, the $I^{2}=8\%$ and the *p*‐value = 0.37 for the Q‐test indicated a lower BSH for movement score. These results suggest that movement score, a subjective trait, has been evaluated similarly across studies. Although the estimates of heritability for movement score ranged between 0.08 ± 0.03 (Freitas et al., 2023) and 0.29 ± 0.10 (Benhajali et al., 2010), our meta‐analysis suggests a low weighted heritability for this trait because the prediction value ranged from 0.08 to 0.15.

Peixoto et al. (2016) evaluated Guzerat (*Bos taurus indicus*) females (lactating and non‐lactating) of several ages (calves, heifers, and cows), which were raised on an extensive pasture‐based system. Guzerat is a dual‐purpose breed. Although the authors reported their studied population as dairy cattle, young and non‐lactating Guzerat, when raised on extensive systems, have a temperament more similar to beef than dairy cattle due to reduced interactions with humans. Some of our results showed no problem grouping the Guzerat heritability estimate with the other heritability estimates reported for movement score in the beef cattle population. For instance: (1) the estimate reported for Guzerat was not identified as an influent or outlier in the meta‐analysis of estimates for movement score, and (2) movement score was a trait with low and non‐significant BSH (*I* ^*2*^ = 8% and Q‐test with *p*‐value = 0.36). These results also suggest that future meta‐analyses with temperament‐related traits should focus on the trait definition rather than the commercial purpose of the herd (beef or dairy).

A crush score (or chute score) is assigned while the animal is restrained in a chute with headgates but without having motion restricted by squeeze. Our review found many estimates of heritability for crush score at weaning (Beckman et al., 2007; Celestino et al., 2020; Halloway & Johnston, 2003; Hearnshaw & Morris, 1984; Hoppe et al., 2010; Kadel et al., 2006; Torres‐Vázquez & Spangler, 2016; Walkom et al., 2018; Yu et al., 2020). The heritability estimates ranged from 0.03 ± 0.28 (Hearnshaw & Morris, 1984) to 0.46 ± 0.03 (Walkom et al., 2018). Our results showed that the estimates reported for Limousin cattle by Beckman et al. (2007) (0.38 ± 0.03) and Walkom et al. (2018) (0.46 ± 0.03) are outliers. Moreover, the estimate that Walkom et al. (2018) was also identified as an influence record on this meta‐analysis, increasing the BSH. This does not mean that the estimates reported for Limousin are wrong; they are just incompatible with the weighted heritability estimated. Perhaps the Limousin populations used in these studies have greater genetic variability for temperament than those used in this crush score meta‐analysis.

After removing the Limousin estimates, the *I* ^*2*^ was reduced from 85% to 50%. However, different from the movement score, the test‐Q to evaluate the BSH for crush score at weaning was significant (*p* = 0.02). Therefore, even using a single age class, this trait has an expressive heterogeneity across studies. On the other hand, the crush score showed a moderate weighted heritability, and its 95% CI (0.17–0.25) did not overlap with the confidence interval estimated for the movement score (0.08–0.15). Therefore, these results suggest that the crush score has a higher heritability than the movement score and might be a better option than the movement score for breeding purposes.

### 4.4 Pen score in beef cattle

In the present meta‐analysis, the heritability estimates for pen score were reported by studies that evaluated the animal's behaviour when restrained in a small pen, with the observer inside the pen. The systematic review found several heritability estimates for pen score at weaning (Celestino et al., 2020; Fordyce et al., 1996; Gauly et al., 2001; Littlejohn et al., 2018; Morris et al., 1994; Riley et al., 2014; Schmidt et al., 2014; Yu et al., 2020) and at yearling (Barrozo et al., 2012; Fordyce et al., 1982, 1996; Lucena et al., 2015; Morris et al., 1994; Neves et al., 2014; Sant'Anna et al., 2015; Valente et al., 2015, 2017). These studies reported heritability estimates for pen score at weaning ranging from 0.14 ± 0.11 (Fordyce et al., 1996) to 0.61 ± 0.17 (Gauly et al., 2001), while the estimates at yearling ranged from 0.11 ± 0.08 (Neves et al., 2014) to 0.29 ± 0.23 (Morris et al., 1994). The estimates reported by Riley et al. (2014) (0.47 ± 0.07) and Schmidt et al. (2014) (0.49 ± 0.06) were identified as outliers. Moreover, the heritability estimates from these two studies greatly contributed to the overall heterogeneity, and the diagnostic plot indicated both estimates as potential influent estimates. The present meta‐analysis yielded a moderate weighted heritability for pen score at weaning (0.27) and yearling (0.20). The Q‐test for subgroup differences was significant, i.e., the pen score at weaning has a weighted heritability slightly higher than at yearling. It may provide a higher genetic gain than the pen score at yearling. Although the previous studies have used different approaches to evaluate the animal's reaction to human presence inside the pen, the meta‐analysis found moderate and low‐heterogeneity index for pen score at weaning (*I* ^2^ = 42%) and yearling (*I* ^2^ = 0%), respectively. Moreover, the Q‐test was not significant for both meta‐analyses. Therefore, these results suggest that the different approaches to measuring pen scores across studies did not affect the heritability estimate for this trait.

### 4.5 Cow's aggressiveness at calving in beef cattle

Newborn calves are handled to receive their first health treatments, weighting, and identification. For this handling, the newborn calves need to be restrained by humans. Thus, some previous beef cattle studies (Buddenberg et al., 1986; Hoppe et al., 2008; Morris et al., 1994; Vallée et al., 2015) recorded the cow's reactivity while their newborn calves are being handled. Heritability estimates for cow's aggressiveness at calving ranged from 0.06 ± 0.01 (Buddenberg et al., 1986) to 0.42 ± 0.05 (Hoppe et al., 2008). However, the meta‐analysis found an estimate of 0.42 ± 0.05. Moreover, the estimate that Hoppe et al. (2008) reported considerably contributed to overall heterogeneity. After this outlier exclusion, the weighted heritability was 0.10, with a confidence interval from 0.01 to 0.19. Although the weighted heritability estimate is low, it should be noted that the prediction interval has a wide range (−0.13 to 0.33), which indicates that further studies are needed to estimate a more reliable weighted heritability for this trait.

### 4.6 General temperament and milking temperament in dairy cattle

Several studies were identified as outliers and excluded from the milking and general temperaments meta‐analyses. Some studies showed high and unusual heritability for these traits [Cue et al., 1996 (0.33 ± 0.06) and Kramer et al., 2013 (0.38 ± 0.07)], while other studies estimated heritability with a very reduced standard error [Pryce et al., 2000 (0.07 ± 0.001), Wethal et al., 2020 (0.09 ± 0.006); and Szymik et al., 2021 (0.09 ± 0.0012)]. Moreover, the estimates reported by Pryce et al. (2000) and Szymik et al. (2021) significantly contributed to the overall heterogeneity. The $I^{2}$ before excluding these estimates was 89% (general temperament) and 91% (milking temperament), and even with the exclusion of outliers, high $I^{2}$ values were found for both milking temperament (69%) and general temperament (77%), and the Q‐test for BSH was significant (*p*‐value < 0.05) for both traits. Initially, we thought that the high number of breeds (Holstein, Holstein‐Gyr cross, Brown Swiss, Norwegian Red and Jersey) in this subgroup meta‐analysis was the reason for the high BSH. Then, we performed a subgroup meta‐analysis (Figure S33) with the studies that included data only from the Holstein breed (Cue et al., 1996; Erf et al., 1992; Foster et al., 1988; Lawstuen et al., 1988; Pryce et al., 2000; Sewalem et al., 2011; Stephansen et al., 2018; Szymik et al., 2021; Thompson et al., 1981; Visscher & Goddard, 1995), but the BSH remained high for both general (75%) and milking temperament (92%). Another hypothesis for these high BSH values is the non‐standardization of the class of age, and a previous study with Holstein cattle found a significant difference between multiparous and primiparous cows for milking temperament (Szentléleki et al., 2015). However, the meta‐analysis using only data from first‐parity cows (Figure S34) also found a high heterogeneity index for both general (82%) and milking temperament (92%). Therefore, different phenotyping methods may have been used across studies of these traits.

Regardless of the level of BSH for temperament‐related traits in dairy cattle, which was adjusted by fitting the random effect model, it was observed that the weighted heritability was low for both general and milking temperament. Furthermore, the 95% CIs do not indicate moderate heritability for these traits. Therefore, although many studies (Cue et al., 1996; Kramer et al., 2014; Sewalem et al., 2011; Visscher & Goddard, 1995) have reported moderate heritability estimates, with values ranging from 0.20 to 0.38, most studies reported low estimates. This low heritability may be associated with the continuous handling that calves and heifers receive until adulthood. Frequent gentle contact with people can result in greater habituation during handling than animals with previous aversive treatment (Grandin, 1989). Finally, the subgroup meta‐analysis did not find a significant difference (*p*‐value > 0.05) between general and milking temperament approaches. These results suggest that the breeding goal and ease of phenotyping are the main factors in choosing one of these traits, as there is no reason to believe one has a higher heritability. However, future studies should also investigate the genetic correlation between these traits.

### 4.7 Final considerations and next steps

This first meta‐analysis study included various temperament‐related traits from beef and dairy cattle studies. However, many questions remain unanswered. For instance, the breed might be a critical fixed effect in a meta‐regression model to perform meta‐analyses. However, there were not enough heritability estimates per breed to compare two or more breeds for any temperament‐related traits evaluated in this study. The same is true for other factors such as production systems (intensive × extensive). As more heritability estimates become available, including these factors in a meta‐regression model can reduce the heterogeneity and result in more accurate weighted estimates.

Another topic not addressed in the present study is the weighted genetic correlations among temperament traits and between temperament and other relevant traits such as performance, product quality (meat or milk), health, and welfare. For instance, in North American Angus cattle, Alvarenga et al. (2022) reported genetic correlations between temperament and other relevant traits ranging from −0.05 to 0.28, which were non‐significant or favourable, which suggests that genetic selection to improve temperament will not negatively affect beef cattle performance. In dairy cattle, calmer cows have better milk performance (Chang et al., 2020), indicating that selection for better temperament will result in favourable genetic responses on milk production and vice‐versa. Unfortunately, not enough estimates were reported in the literature to obtain accurate weighted genetic correlation estimates. For instance, Gathura et al. (2020) reported a weighted genetic correlation between temperament and other production traits for beef cattle. However, they used few studies and did not report the 95% CI of the weighted estimates. Therefore, more studies are needed to understand better the genetic relationships between temperament and other relevant traits in cattle populations.

The score classes varied for many temperament‐related traits, especially beef cattle traits. For instance, various scales of pen score have been reported such as scores ranging from 1 to 4 (Barrozo et al., 2012), 1 to 5 (Celestino et al., 2020), 1 to 6 (Morris et al., 1994), 1 to 7 (Fordyce et al., 1982), 1 to 9 (Riley et al., 2014) and 1 to 13.5 (Fordyce et al., 1996). However, the Q‐test for BSH was not significant for the pen score. This result suggests that the type of score classes did not influence the weighted heritability estimate for pen score in this study, and the choice of one of these scoring systems could be based on the ease of assigning the scores. On the other hand, the lack of standardization of scoring systems may cause high BSH observed for milking and general temperament. In our study, classes from 1 to 3 (Thompson et al., 1981), 1 to 5 (Visscher & Goddard, 1995), 1 to 9 (Pryce et al., 2000), and 1 to 50 (Foster et al., 1988) were observed for general temperament across studies. For milking temperament, classes such as 1 to 3 (Wethal et al., 2020), 1 to 4 (Kramer et al., 2013), 1 to 5 (Sewalem et al., 2011), 1 to 8 (Taborda et al., 2023), and 1 to 9 (Cue et al., 1996) were observed. It was not possible to group the studies according to the scoring system to perform a subgroup meta‐analysis, as each group had a small number of estimates. Therefore, as more studies are published, the effect of the scoring system on the heritability estimates could be assessed in future meta‐analyses of subgroups.

The estimates' standard error (SE) is crucial in meta‐analyses of heritability because they are used to calculate the weights. Heritability estimates with low SE have higher weights in the meta‐analysis than estimates with higher SE. Unfortunately, 22.6% of the studies did not report SE for temperament‐related traits in cattle. Although many of these studies were published years ago (e.g., Dickson et al., 1970), some recent studies did not report SE (e.g., Antanaitis et al., 2021). It is strongly recommended that SE is reported in future genetic parameter studies, as SE values are essential for inferring the accuracy of the heritability estimates.

Another potential cause of high BSH for milking and general temperament scores may be the method used to estimate heritability and standard errors. REML and Bayesian analyses using Gibbs sampling were more commonly used to estimate heritability for temperament‐related traits in cattle. According to Rameez et al. (2022), the REML method implemented in standard software packages such as WOMBAT, DMU and ASREML, among others, uses the Delta method, which is a standard procedure to obtain the sampling variance of heritability by linearly approximating the function with its first‐order Taylor series expansion. All the studies that reported using iterative algorithms for REML analysis did not specify whether the Taylor expansion was used. Therefore, it was not possible to filter estimates based on this criterion. Therefore, it is recommended that future studies regarding genetic parameters provide more details about the methods and population structure (e.g., number of sires and dams in the pedigree, number of dams per sire, relationship between sires, inbreeding), which can be useful additional information to select better the studies to be included in a meta‐analysis.

The trait definition also has a key role in meta‐analysis studies since a group of estimates is formed based on the definition of the traits. Ten studies were not used in the present study because no (or poorly) trait description was reported in the publication. This is especially important in temperament‐related traits, where different names are often used for the same trait. Therefore, assigning estimates to a specific group for meta‐analysis is only possible with a detailed trait description. When describing the traits, citing the article that first published the method and any variations (modifications) about the proposed initial method is strongly recommended. This information will contribute to future updates of this heritability meta‐analysis and other meta‐analyses in the context of temperament traits in cattle.

Many temperament‐related traits can be used to select cattle for improved temperament. The heritability estimate is a factor of great importance for breeding programs when choosing the indicator traits to include in their selection schemes. Assuming the classical scale of heritability, where estimates less than 0.20 are assumed as low, from 0.2 to 0.4 are moderate and greater than 0.4 are high, previous studies have estimated heritability values ranging from low to high for most temperament‐related traits (Chang et al., 2020; Haskell et al., 2014). However, our meta‐analyses estimated moderate weighted heritability for flight speed, flight score, pen score, and crush score. On the other hand, low weighted heritabilities were estimated for movement score, cow's aggressiveness at calving, milking temperament and general temperament.

## 5 CONCLUSIONS

The systematic review identified many studies reporting heritability estimates for various temperament‐related traits in cattle over the last six decades. These were carried out for different breeds from many countries, but many heritability estimates still need to be calculated for various traits across cattle populations. In the past 20 years, the number of beef cattle studies on this topic has increased expressively, which reveals the sector's concern with cattle temperament. Also, there were more heritability estimates for temperament‐related traits in beef (six traits) than in dairy (two traits) cattle populations. Although most of the older studies were carried out on dairy cattle, new methods of assessing dairy cattle temperament are being developed based on modern sensors and technologies. This meta‐analysis showed higher between‐study heterogeneity in dairy than in beef cattle studies, revealing the need for greater standardization of the dairy cattle studies. The heritability estimates reported in the literature ranged from low to high for all traits. However, the weighted heritabilities estimated by the meta‐analyses varied from low to moderate, depending on the trait evaluated. Flight speed was the only trait with moderate weighted heritability, including moderate values for the 95% CI limits. For the first time, subgroup meta‐analyses were performed for temperament‐related traits in cattle, and a significant effect was found when pen scores at weaning and yearling were analysed. However, the number of heritability estimates is still small to carry out better subgroup meta‐analyses for some traits. Overall, the results reported in this study indicate that cattle temperament is heritable and can be improved through genetic selection.

## FUNDING INFORMATION

No funding was received for this manuscript.

## CONFLICT OF INTEREST STATEMENT

The authors declare no competing interests.

## Supporting information


Figures S1–S34.

## Data Availability

Data sharing is not applicable to this article as no new data were created or analysed in this study.

## References

[jbg12874-bib-0001] Agyemang, K. , Clapp, E. , & Van Vleck, L. D. (1982). Components of variance of Dairymen's workability traits among Holstein cows. Journal of Dairy Science, 65(7), 1334–1338. 10.3168/jds.S0022-0302(82)82350-3

[jbg12874-bib-0002] Aitchison, T. E. , Freeman, A. E. , & Thomson, G. M. (1972). Evaluation of a type appraisal program in Holsteins. Journal of Dairy Science, 55(6), 840–844. 10.3168/jds.S0022-0302(72)85579-6

[jbg12874-bib-0003] Alvarenga, A. B. , Oliveira, H. R. , Miller, S. P. , Silva, F. F. , & Brito, L. F. (2022). Genetic modeling and genomic analyses of yearling temperament in American Angus cattle and its relationship with productive efficiency and resilience traits. Frontiers in Genetics, 13, 794625. 10.3389/fgene.2022.794625 35444687 PMC9014094

[jbg12874-bib-0004] Alvarenga, A. B. , Oliveira, H. R. , Turner, S. P. , Garcia, A. , Retallick, K. J. , Miller, S. P. , & Brito, L. F. (2023). Unraveling the phenotypic and genomic background of behavioral plasticity and temperament in north American Angus cattle. Genetics Selection Evolution, 55, 3. 10.1186/s12711-023-00777-3 PMC9850537 36658485

[jbg12874-bib-0501] Antanaitis, R. , Juozaitienė, V. , Jonike, V. , Čukauskas, V. , Urbšienė, D. , Urbšys, A. , Baumgartner, W. , & Paulauskas, A. (2021). Relationship between temperament and stage of lactation, productivity and milk composition of dairy cows. Animals, 11(7), 1840. 10.3390/ani11071840 34206163 PMC8300410

[jbg12874-bib-0005] Barrozo, D. , Buzanskas, M. E. , Oliveira, J. A. , Munari, D. P. , Neves, H. H. R. , & Queiroz, S. A. (2012). Genetic parameters and environmental effects on temperament score and reproductive traits of Nellore cattle. Animal, 6(1), 36–40. 10.1017/S1751731111001169 22436152

[jbg12874-bib-0006] Baujat, B. , Mahé, C. , Pignon, J. P. , & Hill, C. (2002). A graphical method for exploring heterogeneity in meta‐analyses: Application to a meta‐analysis of 65 trials. Statistics in Medicine, 21(18), 2641–2652. 10.1002/sim.1221 12228882

[jbg12874-bib-0007] Beckman, D. W. , Enns, R. M. , Speidel, S. E. , Brigham, B. W. , & Garrick, D. J. (2007). Maternal effects on docility in Limousin cattle. Journal of Animal Science, 85(3), 650–657. 10.2527/jas.2006-450 17040937

[jbg12874-bib-0008] Benhajali, H. , Boivin, X. , Sapa, J. , Pellegrini, P. , Boulesteix, P. , Lajudie, P. , & Phocas, F. (2010). Assessment of different on‐farm measures of beef cattle temperament for use in genetic evaluation. Journal of Animal Science, 88(11), 3529–3537. 10.2527/jas.2010-3132 20693414

[jbg12874-bib-0502] Boivin, X. , Le Neindre, P. , Chupin, J. M. , Garel, J. P. , & Trillat, G. (1992). Influence of breed and early management on ease of handling and open‐field behavior of cattle. Applied Animal Behaviour Science, 32, 313–323. 10.1016/S0168-1591(05)80024-3

[jbg12874-bib-0009] Borenstein, M. , Hedges, L. V. , Higgins, J. P. T. , & Rothstein, H. R. (2010). A basic introduction to fixed‐effect and random‐effects models for meta‐analysis. Research Synthesis Methods, 1(2), 97–111. 10.1002/jrsm.12 26061376

[jbg12874-bib-0010] Buddenberg, B. J. , Brown, C. J. , Johnson, Z. B. , & Honea, R. S. (1986). Maternal behavior of beef cows at parturition. Journal of Animal Science, 62(1), 42–46. 10.2527/jas1986.62142x 3957810

[jbg12874-bib-0011] Burrow, H. M. (2001). Variances and covariances between productive and adaptive traits and temperament in a composite breed of tropical beef cattle. Livestock Production Science, 70(3), 213–233. 10.1016/S0301-6226(01)00178-6

[jbg12874-bib-0012] Burrow, H. M. , Seifert, G. W. , & Corbet, N. J. (1988). A new technique for measuring temperament in cattle. Proceedings of the Australian Society of Animal Production, 17, 154–157.

[jbg12874-bib-0013] Celestino, E. F. , Hieber, J. K. , Dahlen, C. R. , Riley, D. G. , Wagner, S. A. , & Hulsman Hanna, L. L. (2020). Evaluator effect on the prediction of genetic merit using subjective measures of beef cattle temperament. Journal of Animal Science, 98(Supplement_3), 161–162. 10.1093/jas/skaa054.285

[jbg12874-bib-0014] Chang, Y. , Brito, L. F. , Alvarenga, A. B. , & Wang, Y. (2020). Incorporating temperament traits in dairy cattle breeding programs: Challenges and opportunities in the phenomics era. Animal Frontiers, 10(2), 29–36. 10.1093/af/vfaa006 PMC7111596 32257601

[jbg12874-bib-0015] Cochran, W. G. (1954). The combination of estimates from different experiments. Biometrics, 10, 101–129.

[jbg12874-bib-0016] Copley, J. P. , Corbet, N. J. , Allen, J. M. , Laing, A. R. , Fordyce, G. , Mcgowan, M. R. , Burns, B. M. , Lyons, R. E. , & Hayes, B. J. (2022). Understanding the genetics of fertility and temperament in northern beef cattle using genomic technologies . Proceedings of 12th World Congress on Genetics Applied to Livestock Production (WCGALP), pp. 2684–2687.

[jbg12874-bib-0017] Corbet, N. J. , Burns, B. M. , Johnston, D. J. , Wolcott, M. L. , Corbet, D. H. , Venus, B. K. , Li, Y. , McGowan, M. R. , & Holroyd, R. G. (2013). Male traits and herd reproductive capability in tropical beef cattle. 2. Genetic parameters of bull traits. Animal Production Science, 53(2), 101–113. 10.1071/AN12163

[jbg12874-bib-0018] Cue, R. I. , Harris, B. L. , & Rendel, J. M. (1996). Genetic parameters for traits other than production in purebred and crossbred New Zealand dairy cattle. Livestock Production Science, 45(2–3), 123–135.

[jbg12874-bib-0503] Danchuk, O. V. , Karposvkii, V. I. , Tomchuk, V. A. , Zhurenko, O. V. , Bobryts'ka, O. M. , & Trokoz, V. O. (2020). Temperament in cattle: A method of evaluation and main characteristics. Neurophysiology, 52(1), 73–79. 10.1007/s11062-020-09853-6

[jbg12874-bib-0019] Dawson, D. V. , Pihlstrom, B. L. , & Blanchette, D. R. (2016). Understanding and evaluating meta‐analysis. Journal of the American Dental Association, 147(4), 264–270. 10.1016/j.adaj.2015.10.023 26705602

[jbg12874-bib-0020] Dickson, D. P. , Barr, G. R. , Johnson, L. P. , & Wieckert, D. A. (1970). Social dominance and temperament of Holstein cows. Journal of Dairy Science, 53(7), 904–907. 10.3168/jds.S0022-0302(70)86316-0

[jbg12874-bib-0021] Erf, D. F. , Hansen, L. B. , & Lawstuen, D. A. (1992). Inheritance and relationships of workability traits and yield for Holsteins. Journal of Dairy Science, 75(7), 1999–2007. 10.3168/jds.S0022-0302(92)77959-4

[jbg12874-bib-0022] Fordyce, G. , & Goddard, M. E. (1984). Maternal influence on the temperament of Bos indicus cross cows. Proceedings of the Australian Society of Animal Production, 15, 345–348.

[jbg12874-bib-0023] Fordyce, G. , Goddard, M. E. , & Seifert, G. W. (1982). The measurement of temperament in cattle and the effect of experience and genotype. Proceedings of the Australian Society of Animal Production, 14, 329–332.

[jbg12874-bib-0024] Fordyce, G. , Howitt, C. J. , Holroyd, R. G. , O'Rourke, R. K. , & Entwistle, K. W. (1996). The performance of Brahman‐shorthorn and Sahiwal‐shorthorn beef cattle in the dry tropics of northern Queensland. 5. Scrotal circumference, temperament, ectoparasite resistance, and the genetics of growth and other traits in bulls. Australian Journal of Experimental Agriculture, 36(1), 9–17.

[jbg12874-bib-0025] Foster, W. W. , Freeman, A. E. , Berger, P. J. , & Kuck, A. (1988). Linear type trait analysis with genetic parameter estimation. Journal of Dairy Science, 71(1), 223–231. 10.3168/jds.S0022-0302(88)79545-4

[jbg12874-bib-0026] Freitas, A. P. , Lima, M. L. P. , Simili, F. F. , Schenkel, F. S. , Faro, L. E. , Santana, M. L. , & Paz, C. C. P. (2023). Genetic parameters for behavioral and growth traits of Nellore cattle. Journal of Animal Science, 101, skad280. 10.1093/jas/skad280 37624655 PMC10494874

[jbg12874-bib-0027] Gathura, D. M. , Muasya, T. K. , & Kahi, A. K. (2020). Meta‐analysis of genetic parameters for traits of economic importance for beef cattle in the tropics. Livestock Science, 242, 104306. 10.1016/j.livsci.2020.104306

[jbg12874-bib-0028] Gauly, M. , Mathiak, H. , & Erhardt, G. (2002). Genetic background of behavioural and plasma cortisol response to repeated short‐term separation and tethering of beef calves. Journal of Animal Breeding and Genetics, 119(6), 379–384. 10.1046/j.1439-0388.2002.00360.x

[jbg12874-bib-0029] Gauly, M. , Mathiak, H. , Hoffmann, K. , Kraus, M. , & Erhardt, G. (2001). Estimating genetic variability in temperamental traits in German Angus and Simmental cattle. Applied Animal Behaviour Science, 74(2), 109–119. 10.1016/S0168-1591(01)00151-4

[jbg12874-bib-0504] Gibbons, J. M. , Lawrence, A. B. , & Haskell, M. J. (2011). Consistency of flight speed and response to restraint in a crush in dairy cattle. Applied Animal Behaviour Science, 131(1–2), 15–20. 10.1016/j.applanim.2011.01.009

[jbg12874-bib-0030] Grandin, T. (1989). Behavioral principles of livestock handling. The Professional Animal Scientist, 5(2), 1–11. 10.15232/S1080-7446(15)32304-4

[jbg12874-bib-0031] Halloway, D. R. , & Johnston, D. J. (2003). Evaluation of flight time and crush score as measures of temperament in Angus cattle . 15th conference of the Association for the Advancement of animal breeding and genetics, pp. 261–264. https://hdl.handle.net/1959.11/4382

[jbg12874-bib-0032] Harrer, M. , Cuijpers, P. , Furukawa, T. A. , & Ebert, D. D. (2022). Doing meta‐analysis with R: A hands‐on guide. Chapman and Hall/CRC.

[jbg12874-bib-0505] Harris, R. J. , Bradburn, M. J. , Deeks, J. J. , Harbord, R. M. , Altman, D. G. , & Sterne, J. A. C. (2008). Metan: Fixed‐ and random‐effects meta‐analysis. The Stata Journal, 8(1), 3–28. 10.1177/1536867X0800800102

[jbg12874-bib-0033] Haskell, M. J. , Simm, G. , & Turner, S. P. (2014). Genetic selection for temperament traits in dairy and beef cattle. Frontiers in Genetics, 5, 368. 10.3389/fgene.2014.00368 25374582 PMC4204639

[jbg12874-bib-0034] Hearnshaw, H. , & Morris, C. A. (1984). Genetic and environmental effects on a temperament score in beef cattle. Australian Journal of Agricultural Research, 35(5), 723–733. 10.1071/AR9840723

[jbg12874-bib-0035] Higgins, J. P. T. , & Thompson, S. G. (2002). Quantifying heterogeneity in a meta‐analysis. Statistics in Medicine, 21(11), 1539–1558. 10.1002/sim.1186 12111919

[jbg12874-bib-0036] Hine, B. C. , Bell, A. M. , Niemeyer, D. D. O. , Duff, C. J. , Butcher, N. M. , Dominik, S. , Ingham, A. B. , & Colditz, I. G. (2019). Immune competence traits assessed during the stress of weaning are heritable and favorably genetically correlated with temperament traits in Angus cattle. Journal of Animal Science, 97(10), 4053–4065. 10.1093/jas/skz260 31581299 PMC6776280

[jbg12874-bib-0037] Hoppe, S. , Brandt, H. R. , Erhardt, G. , & Gauly, M. (2008). Maternal protective behaviour of German Angus and Simmental beef cattle after parturition and its relation to production traits. Applied Animal Behaviour Science, 114(3–4), 297–306. 10.1016/j.applanim.2008.04.008

[jbg12874-bib-0038] Hoppe, S. , Brandt, H. R. , König, S. , Erhardt, G. , & Gauly, M. (2010). Temperament traits of beef calves measured under field conditions and their relationships to performance. Journal of Animal Science, 88(6), 1982–1989. 10.2527/jas.2008-1557 20154170

[jbg12874-bib-0039] Kadel, M. J. , Johnston, D. J. , Burrow, H. M. , Graser, H. U. , & Ferguson, D. M. (2006). Genetics of flight time and other measures of temperament and their value as selection criteria for improving meat quality traits in tropically adapted breeds of beef cattle. Australian Journal of Agricultural Research, 57(9), 1029–1035. 10.1071/AR05082

[jbg12874-bib-0040] King, D. A. , Schuehle Pfeiffer, C. E. , Randel, R. D. , Welsh, T. H. , Oliphint, R. A. , Baird, B. E. , Curley, K. O. , Vann, R. C. , Hale, D. S. , & Savell, J. W. (2006). Influence of animal temperament and stress responsiveness on the carcass quality and beef tenderness of feedlot cattle. Meat Science, 74(3), 546–556. 10.1016/j.meatsci.2006.05.004 22063059

[jbg12874-bib-0041] Knapp, G. , & Hartung, J. (2003). Improved tests for a random effects meta‐regression with a single covariate. Statistics in Medicine, 22(17), 2693–2710. 10.1002/sim.1482 12939780

[jbg12874-bib-0042] Kramer, M. , Erbe, M. , Bapst, B. , Bieber, A. , & Simianer, H. (2013). Estimation of genetic parameters for novel functional traits in Brown Swiss cattle. Journal of Dairy Science, 96(9), 5954–5964. 10.3168/jds.2012-6236 23871377

[jbg12874-bib-0043] Kramer, M. , Erbe, M. , Seefried, F. R. , Gredler, B. , Bapst, B. , Bieber, A. , & Simianer, H. (2014). Accuracy of direct genomic values for functional traits in Brown Swiss cattle. Journal of Dairy Science, 97(3), 1774–1781. 10.3168/jds.2013-7054 24440263

[jbg12874-bib-0044] Lawstuen, D. A. , Hansen, L. B. , Steuernagel, G. R. , & Johnson, L. P. (1988). Management traits scored linearly by dairy producers. Journal of Dairy Science, 71(3), 788–799. 10.3168/jds.S0022-0302(88)79619-8

[jbg12874-bib-0045] Le Neindre, P. , Trillat, G. , Sapa, J. , Ménissier, F. , Bonnet, J. N. , & Chupin, J. M. (1995). Individual differences in docility in Limousin cattle. Journal of Animal Science, 73(8), 2249–2253. 10.2527/1995.7382249x 8567460

[jbg12874-bib-0046] Littlejohn, B. P. , Riley, D. G. , Welsh, T. H. , Randel, R. D. , Willard, S. T. , & Vann, R. C. (2018). Use of random regression to estimate genetic parameters of temperament across an age continuum in a crossbred cattle population. Journal of Animal Science, 96(7), 2607–2621. 10.1093/jas/sky180 29762711 PMC6095449

[jbg12874-bib-0047] Lopez‐Carbonell, D. , Altarriba, J. , Ramírez, M. A. , Srihi, H. , & Varona, L. (2023). Correlaciones genéticas entre caracteres morfológicos y caracteres reproductivos, de crecimiento y calidad de la canal en la raza bovina Pirenaica. Informacion Tecnica Economica Agraria, 113, 225–243. 10.12706/itea.2023.002

[jbg12874-bib-0048] Lucena, C. R. S. , Neves, H. H. R. , Carvalheiro, R. , Oliveira, J. A. , & Queiroz, S. A. (2015). Genetic analysis of the temperament of Nellore cattle using linear and threshold models. Animal, 9(3), 388–394. 10.1017/S1751731114002572 25359241

[jbg12874-bib-0049] Maskal, J. M. , Pedrosa, V. B. , Rojas de Oliveira, H. , & Brito, L. F. (2024). A comprehensive meta‐analysis of genetic parameters for resilience and productivity indicator traits in Holstein cattle. Journal of Dairy Science, 107(5), 3062–3079. 10.3168/jds.2023-23668 38056564

[jbg12874-bib-0050] Morris, C. A. , Cullen, N. G. , Kilgour, R. , & Bremner, K. J. (1994). Some genetic factors affecting temperament in Bos taurus cattle. New Zealand Journal of Agricultural Research, 37(2), 167–175. 10.1080/00288233.1994.9513054

[jbg12874-bib-0051] Mourão, G. B. , Garcia Bergmann, J. A. , & Dias Ferreira, M. B. (1998). Diferenças genéticas e estimação de coeficientes de herdabilidade para temperamento em fêmeas Zebus e F 1 Holandês x Zebu. Revista Brasileira de Zootecnia, 27(4), 722–729.

[jbg12874-bib-0506] Neave, H. W. , Zobel, G. , Thoday, H. , Saunders, K. , Edwards, J. P. , & Webster, J. (2022). Toward on‐farm measurement of personality traits and their relationships to behavior and productivity of grazing dairy cattle. Journal of Dairy Science, 105(7), 6055–6069. 10.3168/jds.2021-21249 35637000

[jbg12874-bib-0052] Neves, H. H. R. , Polin dos Reis, F. , Motta Paterno, F. , Rocha Guarini, A. , Carvalheiro, R. , da Silva, L. R. , de Oliveira, J. A. , & Aidar de Queiroz, S. (2014). Herd‐of‐origin effect on the post‐weaning performance of centrally tested Nellore beef cattle. Tropical Animal Health and Production, 46(7), 1235–1241. 10.1007/s11250-014-0633-2 25015182

[jbg12874-bib-0053] Nkrumah, J. D. , Crews, D. H. , Basarab, J. A. , Price, M. A. , Okine, E. K. , Wang, Z. , Li, C. , & Moore, S. S. (2007). Genetic and phenotypic relationships of feeding behavior and temperament with performance, feed efficiency, ultrasound, and carcass merit of beef cattle. Journal of Animal Science, 85(10), 2382–2390. 10.2527/jas.2006-657 17591713

[jbg12874-bib-0054] Norman, H. D. , & Van‐Vleck, L. D. (1971). Type appraisal: II. Variation in type traits due to sires, herds, and years. Journal of Dairy Science, 55(12), 1717–1725.

[jbg12874-bib-0055] O'Bleness, G. V. , van Vleck, L. D. , & Henderson, C. R. (1960). Heritabilities of some type appraisal traits and their genetic and phenotypic correlations with production. Journal of Dairy Science, 43(10), 1490–1498. 10.3168/jds.S0022-0302(60)90354-4

[jbg12874-bib-0056] Oikawa, T. , Fudo, T. , & Kaneji, K. (1988). Estimate of genetic parameters for temperament and body measurements of beef cattle. Japanese Journal of Zootechnical Science, 60(9), 894–896.

[jbg12874-bib-0057] Oliveira, H. , Ventura, H. T. , Costa, E. V. , Pereira, M. A. , Veroneze, R. , Duarte, M. D. S. , Dias De Siqueira, O. H. G. B. , Fonseca, E. , & Silva, F. (2018). Meta‐analysis of genetic‐parameter estimates for reproduction, growth and carcass traits in Nellore cattle by using a random‐effects model. Animal Production Science, 58(9), 1575–1583. 10.1071/AN16712

[jbg12874-bib-0058] Oliveira‐Junior, G. A. , Schenkel, F. S. , Alcantara, L. , Houlahan, K. , Lynch, C. , & Baes, C. F. (2021). Estimated genetic parameters for all genetically evaluated traits in Canadian Holsteins. Journal of Dairy Science, 104(8), 9002–9015. 10.3168/jds.2021-20227 33934872

[jbg12874-bib-0059] Ouzzani, M. , Hammady, H. , Fedorowicz, Z. , & Elmagarmid, A. (2016). Rayyan‐a web and mobile app for systematic reviews. Systematic Reviews, 5(1), 210. 10.1186/s13643-016-0384-4 27919275 PMC5139140

[jbg12874-bib-0060] Page, M. J. , McKenzie, J. E. , Bossuyt, P. M. , Boutron, I. , Hoffmann, T. C. , Mulrow, C. D. , Shamseer, L. , Tetzlaff, J. M. , Akl, E. A. , Brennan, S. E. , Chou, R. , Glanville, J. , Grimshaw, J. M. , Hróbjartsson, A. , Lalu, M. M. , Li, T. , Loder, E. W. , Mayo‐Wilson, E. , McDonald, S. , … Moher, D. (2021). The PRISMA 2020 statement: An updated guideline for reporting systematic reviews. The BMJ, 372, n71. 33782057 10.1136/bmj.n71 PMC8005924

[jbg12874-bib-0061] Pedrosa, V. B. , Boerman, J. P. , Gloria, L. S. , Chen, S. Y. , Montes, M. E. , Doucette, J. S. , & Brito, L. F. (2023). Genomic‐based genetic parameters for milkability traits derived from automatic milking systems in north American Holstein cattle. Journal of Dairy Science, 106(4), 2613–2629. 10.3168/jds.2022-22515 36797177

[jbg12874-bib-0062] Pedrosa, V. B. , Chen, S. Y. , Gloria, L. S. , Doucette, J. S. , Boerman, J. P. , Rosa, G. J. M. , & Brito, L. F. (2024). Machine learning methods for genomic prediction of cow behavioral traits measured by automatic milking systems in north American Holstein cattle. Journal of Dairy Science. 10.3168/jds.2023-24082 38395400

[jbg12874-bib-0063] Peixoto, M. G. C. D. , Bruneli, F. Â. T. , Bergmann, J. A. G. , Santos, G. G. , Carvalho, M. R. S. , Brito, L. F. , Pereira, M. C. , & Pires, M. F. Á. (2016). Environmental and genetic effects on the temperament variability of Guzerá (Bos indicus) females. Livestock Research for Rural Development, 28(9), 159.

[jbg12874-bib-0507] Pott, A. F. (1918). A study of cattle "temperament" and its measurement. The Ohio Journal of Science, 18(4), 129–144.

[jbg12874-bib-0064] Prayaga, K. C. , Corbet, N. J. , Johnston, D. J. , Wolcott, M. L. , Fordyce, G. , & Burrow, H. M. (2009). Genetics of adaptive traits in heifers and their relationship to growth, pubertal and carcass traits in two tropical beef cattle genotypes. Animal Production Science, 49(6), 413–425. 10.1071/EA08247

[jbg12874-bib-0065] Pryce, J. E. , Coffey, M. P. , & Brotherstone, S. (2000). The genetic relationship between calving interval, body condition score and linear type and management traits in registered Holsteins. Journal of Dairy Science, 83(11), 2664–2671. 10.3168/jds.S0022-0302(00)75160-5 11104287

[jbg12874-bib-0508] Rameez, R. , Jahageerdar, S. , Jayaraman, J. , Chanu, T. I. , Bangera, R. , & Gilmour, A. (2022). Evaluation of alternative methods for estimating the precision of REML‐based estimates of variance components and heritability. Heredity, 128(4), 197–208. 10.1038/s41437-022-00509-1 35197554 PMC8986777

[jbg12874-bib-0066] Riley, D. G. , Gill, C. A. , Herring, A. D. , Riggs, P. K. , Sawyer, J. E. , Lunt, D. K. , & Sanders, J. O. (2014). Genetic evaluation of aspects of temperament in Nellore‐Angus calves. Journal of Animal Science, 92(8), 3223–3230. 10.2527/jas.2014-7797 24879766

[jbg12874-bib-0067] Sant'Anna, A. C. , Baldi, F. , Valente, T. S. , Albuquerque, L. G. , Menezes, L. M. , Boligon, A. A. , & Paranhos da Costa, M. J. R. (2015). Genetic associations between temperament and performance traits in Nellore beef cattle. Journal of Animal Breeding and Genetics, 132(1), 42–50. 10.1111/jbg.12117 25174988

[jbg12874-bib-0068] Sant'Anna, A. C. , Paranhos da Costa, M. J. R. , Baldi, F. , Rueda, P. M. , & Albuquerque, L. G. (2012). Genetic associations between flight speed and growth traits in Nellore cattle. Journal of Animal Science, 90(10), 3427–3432. 10.2527/jas.2011-5044 22585807

[jbg12874-bib-0069] Sato, S. (1981). Factors associated with temperament of beef cattle. Japanese Journal of Zootechnical Science, 52(8), 595–605. 10.2508/chikusan.52.595

[jbg12874-bib-0070] Schmidt, S. E. , Neuendorff, D. A. , Riley, D. G. , Vann, R. C. , Willard, S. T. , Welsh, T. H. , & Randel, R. D. (2014). Genetic parameters of three methods of temperament evaluation of Brahman calves. Journal of Animal Science, 92(7), 3082–3087. 10.2527/jas.2013-7494 24821821

[jbg12874-bib-0071] Sewalem, A. , Miglior, F. , & Kistemaker, G. J. (2011). Genetic parameters of milking temperament and milking speed in Canadian Holsteins. Journal of Dairy Science, 94(1), 512–516. 10.3168/jds.2010-3479 21183064

[jbg12874-bib-0072] Smith, S. P. , Allaire, F. R. , Taylor, W. R. , Kaeser, H. E. , & Conley, J. (1985). Genetic parameters and environmental factors associated with type traits scored on an ordered scale during first lactation. Journal of Dairy Science, 68(8), 2058–2071. 4044969 10.3168/jds.S0022-0302(85)81068-7

[jbg12874-bib-0073] Stephansen, R. S. , Fogh, A. , & Norberg, E. (2018). Genetic parameters for handling and milking temperament in Danish first‐parity Holstein cows. Journal of Dairy Science, 101(12), 11033–11039. 10.3168/jds.2018-14804 30243640

[jbg12874-bib-0074] Szentléleki, A. , Nagy, K. , Széplaki, K. , Kékesi, K. , & Tozsér, J. (2015). Behavioural responses of primiparous and multiparous dairy cows to the milking process over an entire lactation. Annals of Animal Science, 15(1), 185–195. 10.2478/aoas-2014-0064

[jbg12874-bib-0075] Szymik, B. , Topolski, P. , & Jagusiak, W. (2021). Genetic parameters of workability traits in the population of polish holstein‐friesian cows based on conventional and genomic data. Animals, 11(8), 2443. 10.3390/ani11082443 34438899 PMC8388624

[jbg12874-bib-0076] Taborda, P. A. B. , Valente, T. S. , de Lima Carvalhal, M. V. , da Silva, M. V. G. B. , & Paranhos da Costa, M. J. R. (2023). Estimation of genetic parameters for milking temperament in Holstein‐Gyr cows. Frontiers in Animal Science, 4, 1187273. 10.3389/fanim.2023.1187273

[jbg12874-bib-0077] Thompson, J. R. , Freeman, A. E. , Wilson, D. J. , Chapin, C. A. , Berger, P. J. , & Kuck, A. (1981). Evaluation of a linear type program in Holsteins. Journal of Dairy Science, 64(7), 1610–1617. 10.3168/jds.S0022-0302(81)82733-6

[jbg12874-bib-0509] Torres‐Vázquez, J. A. , & Spangler, M. L. (2016). Genetic parameters for docility, weaning weight, yearling weight, and intramuscular fat percentage in Hereford cattle. Journal of Animal Science, 94(1), 21–27. 10.2527/jas.2015-9566 26812308

[jbg12874-bib-0510] Tulloh, N. M. (1961). Behaviour of cattle in yards. II. A study of temperament. Animal Behaviour, 9, 25–30. 10.1016/0003-3472(61)90046-X

[jbg12874-bib-0078] Valente, T. S. , Albito, O. D. , Sant'Anna, A. C. , Carvalheiro, R. , Baldi, F. , Albuquerque, L. G. , & da Costa, M. J. R. P. (2017). Genetic parameter estimates for temperament, heifer rebreeding, and stayability in Nellore cattle. Livestock Science, 206, 45–50. 10.1016/j.livsci.2017.10.010

[jbg12874-bib-0079] Valente, T. S. , Baldi, F. , Sant'Anna, A. C. , Albuquerque, L. G. , & da Costa, M. J. R. P. (2016). Genome‐wide association study between single nucleotide polymorphisms and flight speed in Nellore cattle. PLoS One, 11(6), e0156956. 10.1371/journal.pone.0156956 27300296 PMC4907449

[jbg12874-bib-0080] Valente, T. S. , Sant'Anna, A. C. , Baldi, F. , Albuquerque, L. G. , & da Costa, M. J. R. P. (2015). Genetic association between temperament and sexual precocity indicator traits in Nellore cattle. Journal of Applied Genetics, 56(3), 349–354. 10.1007/s13353-014-0259-0 25472773

[jbg12874-bib-0081] Vallée, A. , Breider, I. , van Arendonk, J. A. M. , & Bovenhuis, H. (2015). Genetic parameters for large‐scale behavior traits and type traits in Charolais beef cows. Journal of Animal Science, 93(9), 4277–4284. 10.2527/jas.2015-9292 26440327

[jbg12874-bib-0082] Van‐Vleck, L. D. (1964). Variation in type appraisal scores due to sire and herd effects. Journal of Dairy Science, 47(11), 1249–1256. 10.3168/jds.S0022-0302(64)88890-1

[jbg12874-bib-0083] Visscher, P. M. , & Goddard, M. E. (1995). Genetic parameters for Milk yield, survival, workability, and type traits for Australian dairy cattle. Journal of Dairy Science, 78(1), 205–220. 10.3168/jds.S0022-0302(95)76630-9 7738256

[jbg12874-bib-0084] Walkom, S. F. , Jeyaruban, M. G. , Tier, B. , & Johnston, D. J. (2018). Genetic analysis of docility score of Australian Angus and Limousin cattle. Animal Production Science, 58(2), 213–223. 10.1071/AN16240

[jbg12874-bib-0085] Wethal, K. B. , Svendsen, M. , & Heringstad, B. (2020). Are farmer assessed temperament, milking speed, and leakage genetically the same traits in automatic milking systems and traditional milking systems? Journal of Dairy Science, 103(4), 3325–3333. 10.3168/jds.2019-17503 32089305

[jbg12874-bib-0086] Wickham, B. W. (1979). Genetic parameters and economic values of traits other than production for dairy cattle. Proceedings of the New Zealand Society of Animal Production, 39, 180–193.

[jbg12874-bib-0087] Yu, H. , Morota, G. , Celestino, E. F. , Dahlen, C. R. , Wagner, S. A. , Riley, D. G. , & Hulsman Hanna, L. L. (2020). Deciphering cattle temperament measures derived from a four‐platform standing scale using genetic factor analytic modeling. Frontiers in Genetics, 11, 599. 10.3389/fgene.2020.00599 32595702 PMC7304504

