# Supplementary material for: A systematic review with meta‐analysis of heritability estimates for temperament‐related traits in beef and dairy cattle populations
Source: J Anim Breed Genet. 2024 May 29;142(1):1–23. doi: 10.1111/jbg.12874 (PMC11629075; doi:10.1111/jbg.12874)
Supplement: Supplementary file 1 — Figures S1–S34. [file JBG-142-1-s001.docx]

**
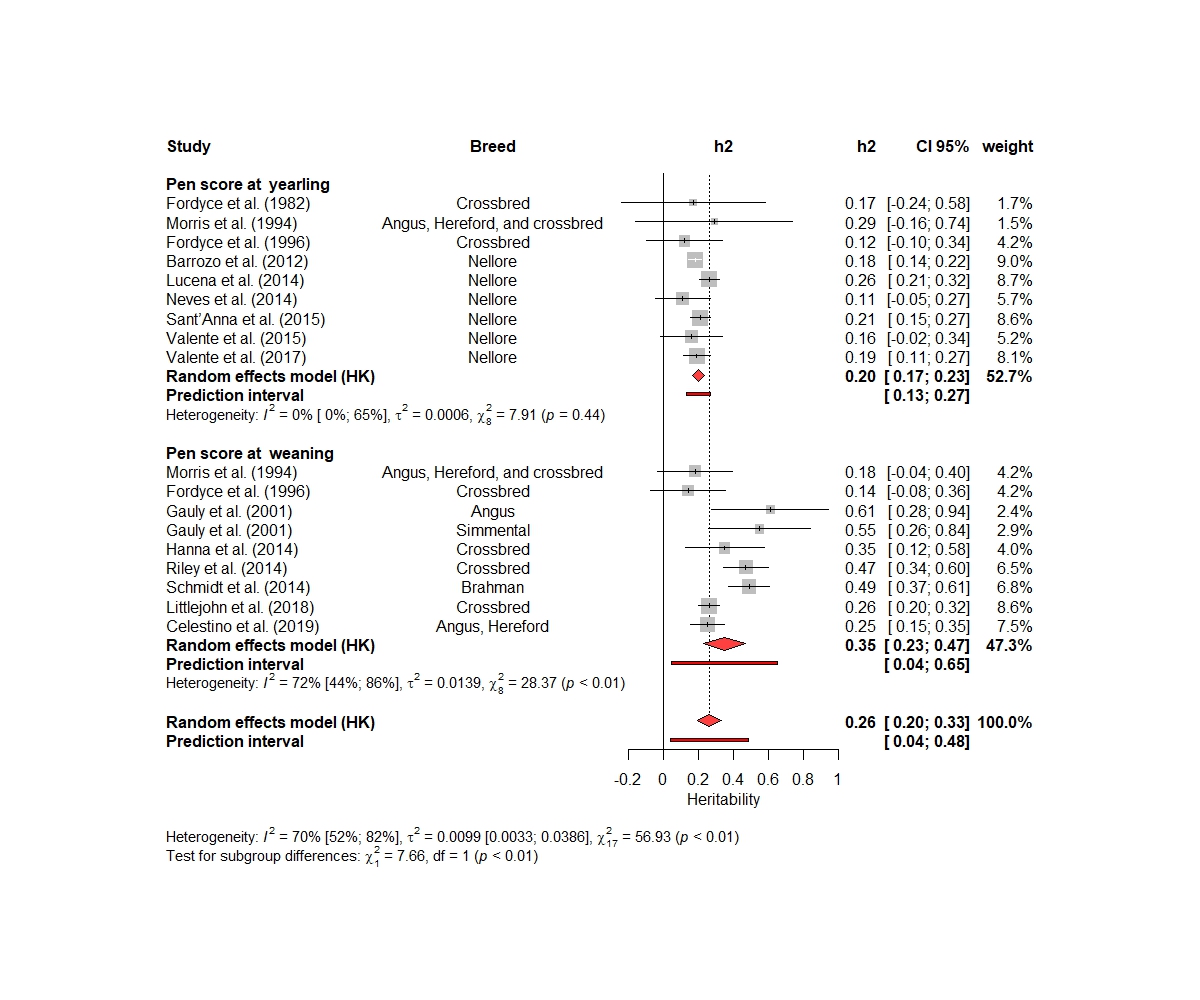
**

**Supplementary Figure S1.** An initial meta-analysis of the pen score, with all studies included.


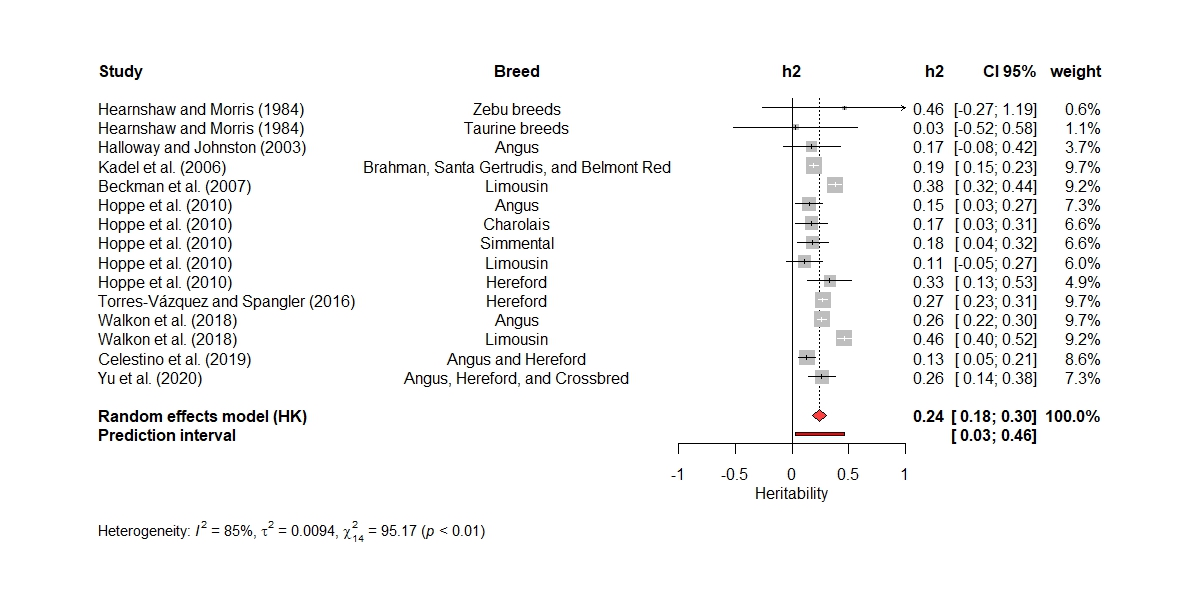


**Supplementary Figure S2.** An initial meta-analysis of the crush score, with all studies included.

**
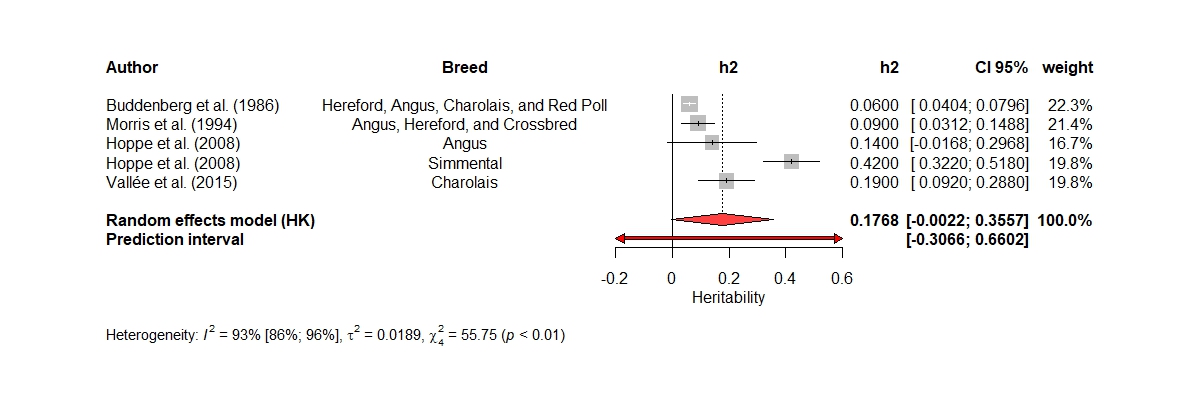
**

**Supplementary Figure S3.** An initial meta-analysis of the cow’s aggressiveness at calving, with all studies included.

**
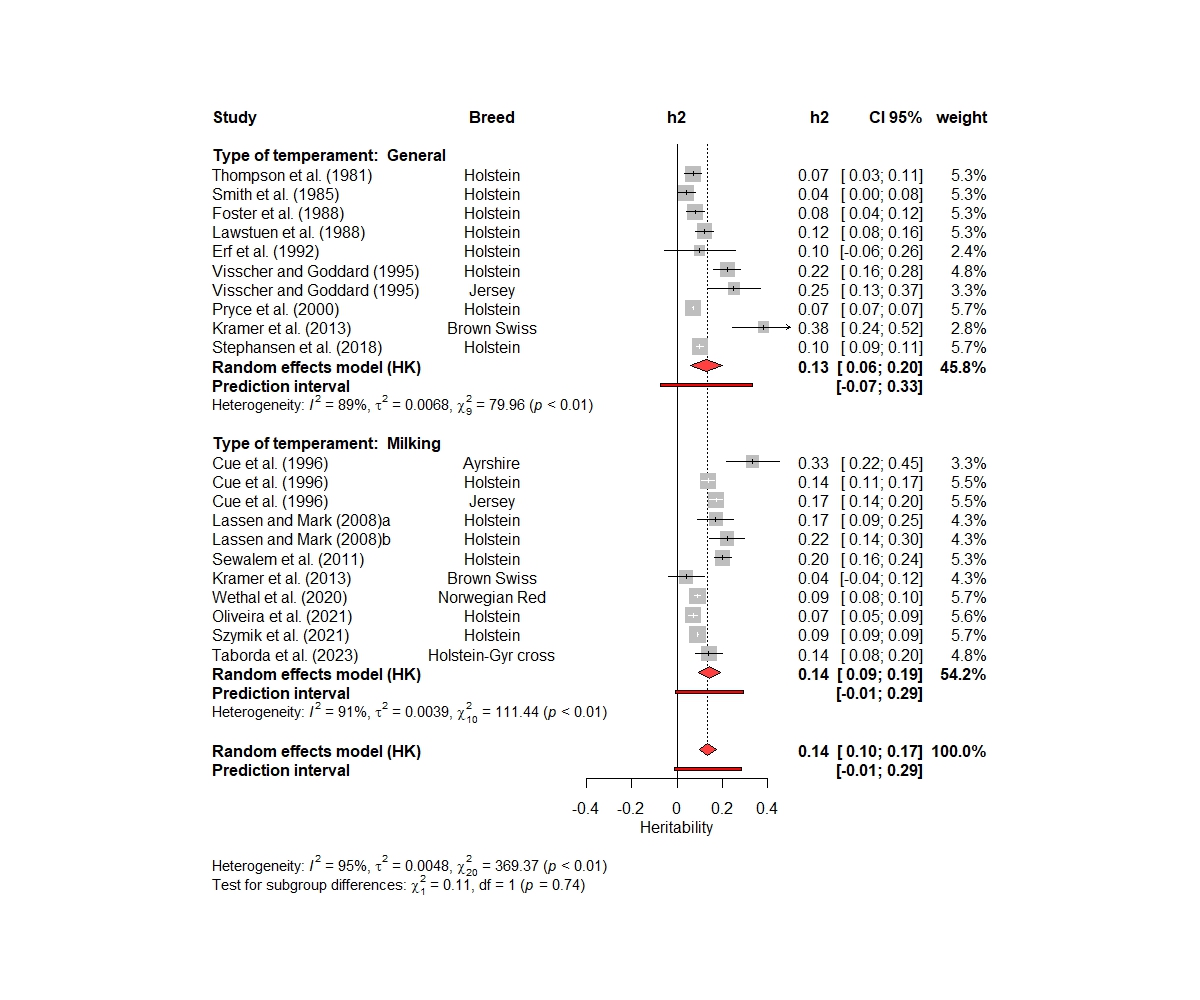
**

**Supplementary Figure S4.** An initial meta-analysis of the general and milking temperament, with all studies included.


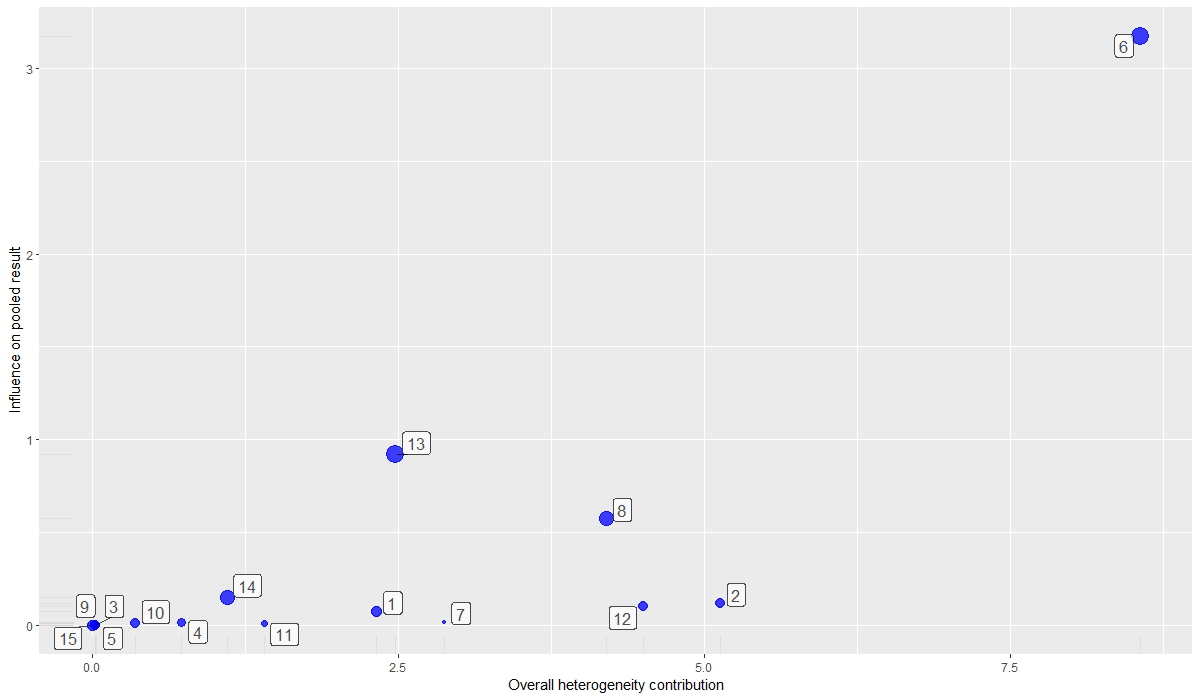


**Supplementary Figure S5.** Baujat plot from the meta-analysis of flight speed x flight score. The code of the estimates were as follows: 1) 0.36±0.06 (Hoppe et al., 2010), 2) 0.11±0.07 (Hoppe et al., 2010), 3) 0.28±0.07 (Hoppe et al., 2010), 4) 0.20±0.08 (Hoppe et al., 2010), 5) 0.25±0.10 (Hoppe et al., 2010), 6) 0.21±0.02 (Kadel et al., 2006), 7) 0.54±0.16 (Burrow et al., 1988), 8) 0.33±0.03 (Copley et al., 2022), 9) 0.28±0.07 (Corbet et al., 2013), 10) 0.31±0.07 (Corbet et al., 2013), 11) 0.13±0.12 (Halloway and Johnston, 2003), 12) 0.12±0.07 (Hine et al., 2019), 13) 0.30±0.02 (Kadel et al., 2006), 14) 0.30±0.03 (Littlejohn et al., 2018), 15) 0.27±0.05 (Schmidt et al., 2014)


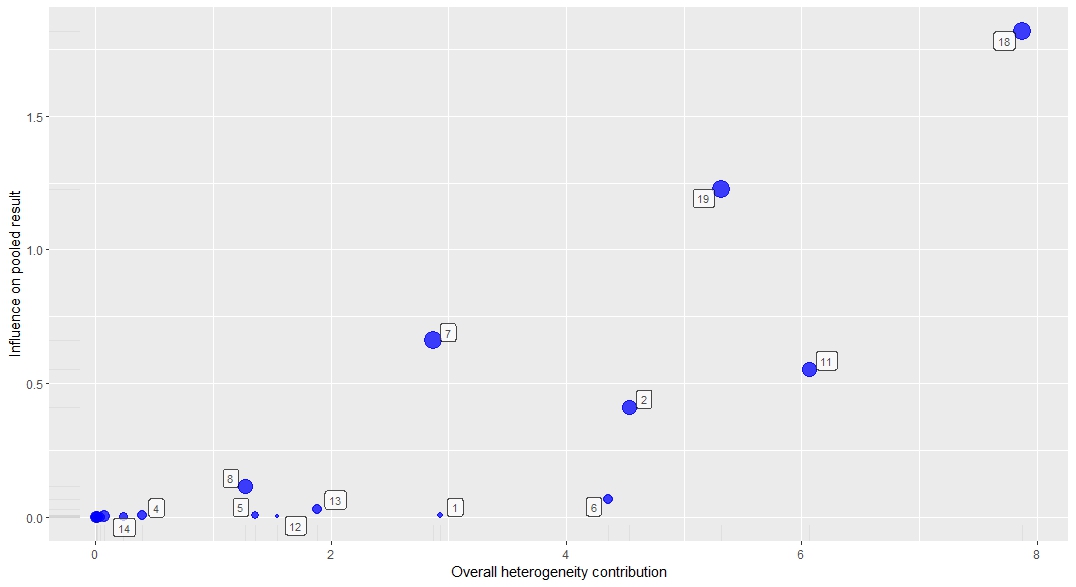


**Supplementary Figure S6.** Baujat plot from the meta-analysis of flight speed at weaning x yearling. The code of the estimates were as follows: 1) 0.54±0.16 (Burrow et al., 1988), 2) 0.33±0.03 (Copley et al., 2022), 3) 0.28±0.07 (Corbet et al., 2013), 4) 0.31±0.07 (Corbet et al., 2013), 5) 0.13±0.12 (Halloway and Johnston, 2003), 6) 0.12±0.07 (Hine et al., 2019), 7) 0.30±0.02 (Kadel et al., 2006), 8) 0.30±0.03 (Littlejohn et al., 2018), 9) 0.27±0.05 (Schmidt et al., 2014), 10) 0.26±0.13 (Burrow et al., 1988), 11) 0.34±0.03 (Kadel et al., 2006), 12) 0.49±0.18 (Nkrumah et al., 2007), 13) 0.17±0.07 (Prayaga et al., 2009), 14) 0.31±0.09 (Prayaga et al., 2009), 15) 0.26±0.05 (Sant’Anna et al., 2012), 16) 0.28±0.05 (Sant’Anna et al., 2015), 17) 0.27±0.07 (Valente et al., 2015), 18) 0.21±0.02 (Valente et al., 2016), 19) 0.22±0.02 (Valente et al., 2017).

**
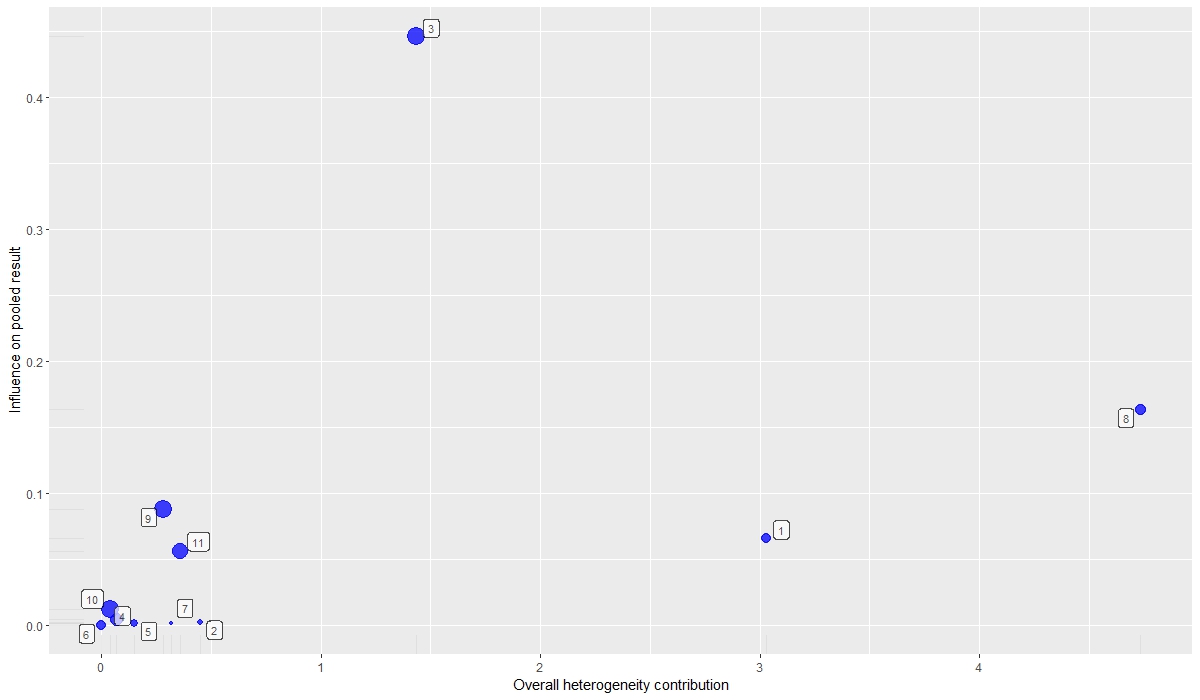
**

**Supplementary Figure S7.** Baujat plot from the meta-analysis of movement score. The code of the estimates were as follows: 1) 0.29±0.10 (Benhajali et al., 2010), 2) 0.25±0.20 (Fordyce et al., 1982), 3) 0.08±0.03 (Freitas et al., 2023), 4) 0.10±0.06 (Hine et al., 2019), 5) 0.17±0.14 (Morris et al., 1994), 6) 0.12±0.10 (Morris et al., 1994), 7) 0.24±0.22 (Morris et al., 1994), 8) 0.29±0.08 (Peixoto et al., 2016), 9) 0.10±0.03 (Sant’Anna et al., 2015), 10) 0.11±0.03 (Valente et al., 2015), and 11) 0.14±0.04 (Valente et al., 2017).


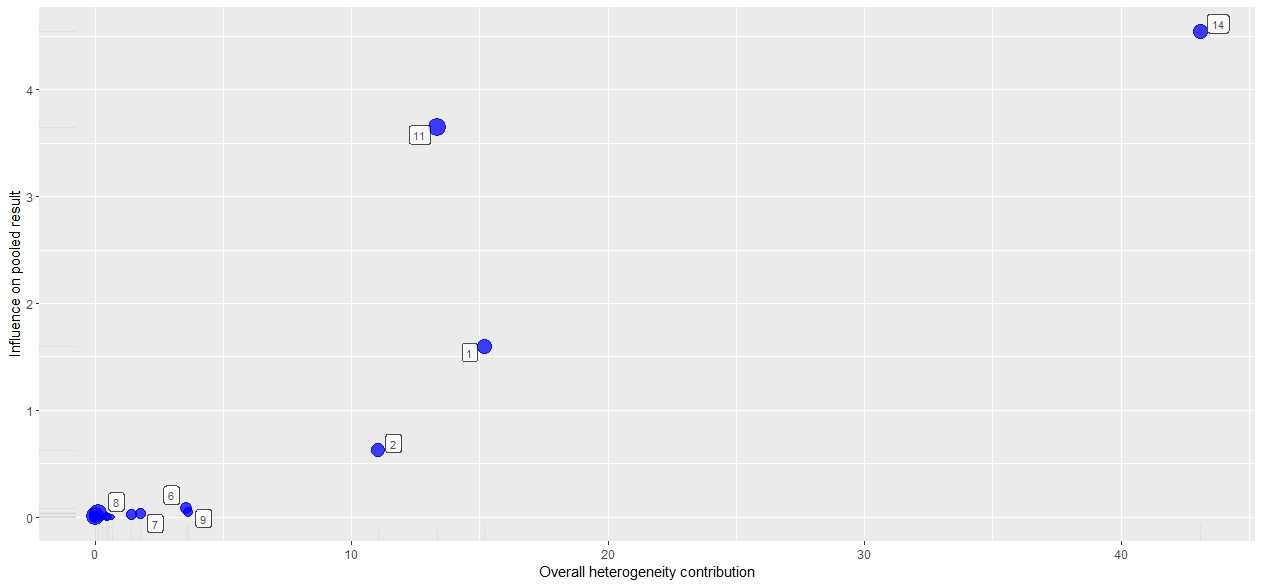


**Supplementary Figure S8.** Baujat plot from the meta-analysis of crush score at weaning. The code of the estimates were as follows: 1) 0.38±0.03 (Beckman et al., 2007), 2) 0.13±0.04 **(**Celestino et al., 2019), 3) 0.17±0.13 (Halloway and Johnston, 2003), 4) 0.46±0.37 (Hearnshaw and Morris, 1984), 5) 0.03±0.28 (Hearnshaw and Morris, 1984), 6) 0.15±0.06 (Hoppe et al., 2010), 7) 0.17±0.07 (Hoppe et al., 2010), 8) 0.18±0.07 (Hoppe et al., 2010), 9) 0.11±0.08 (Hoppe et al., 2010), 10) 0.33±0.10 (Hoppe et al., 2010), 11) 0.19±0.02 (Kadel et al., 2006), 12) 0.27±0.02 (Torres-Vázquez and Spangler, 2016), 13) 0.26±0.02 (Walkon et al., 2018), 14) 0.46±0.03 (Walkon et al., 2018), and 15) 0.26±0.06 (Yu et al., 2020).

**
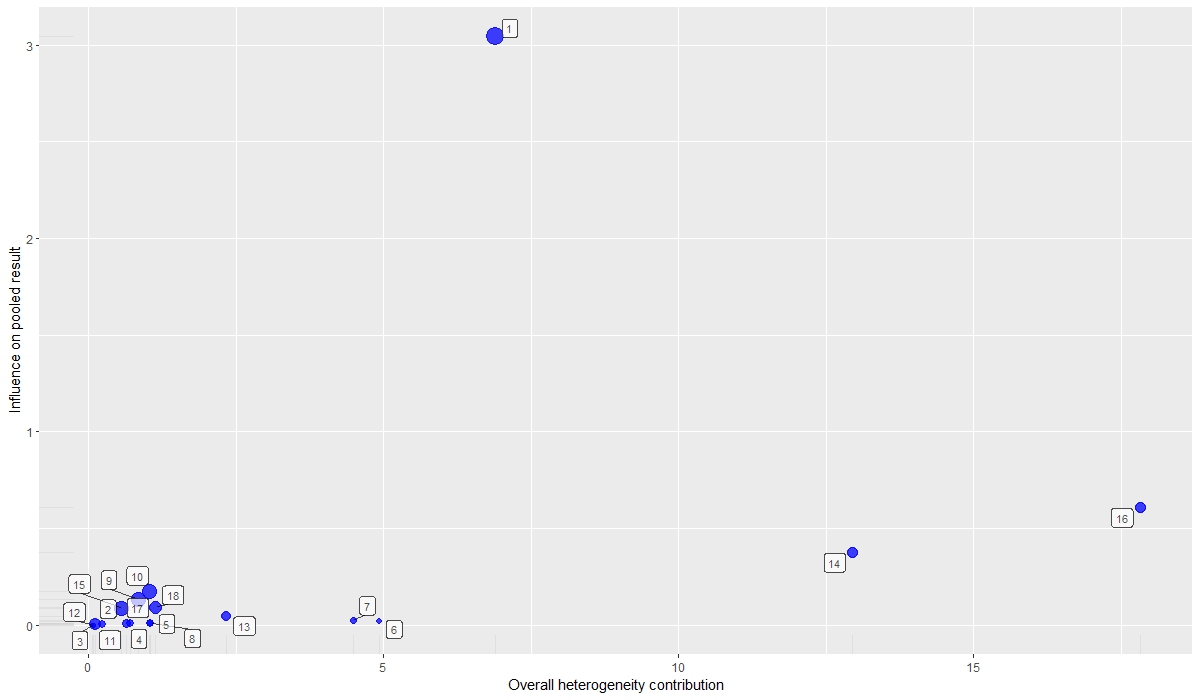
**

**Supplementary Figure S9.** Baujat plot from the meta-analysis of pen score at weaning x yearling. The code of the estimates were as follows: 1) 0.18±0.02 (Barrozo et al., 2012), 2) 0.25±0.05 (Celestino et al., 2019), 3) 0.17±0.21 (Fordyce et al., 1982), 4) 0.14±0.11 (Fordyce et al., 1996), 5) 0.12±0.11 (Fordyce et al., 1996), 6) 0.61±0.17 (Gauly et al., 2001), 7) 0.55±0.15 (Gauly et al., 2001), 8) 0.35±0.12 (Hanna et al., 2014), 9) 0.26±0.03 (Littlejohn et al., 2018), 10) 0.26±0.03 (Lucena et al., 2014), 11) 0.18±0.11 (Morris et al., 1994), 12) 0.29±0.23 (Morris et al., 1994), 13) 0.11±0.08 (Neves et al., 2014), 14) 0.47±0.07 (Riley et al., 2014), 15) 0.21±0.03 (Sant’Anna et al., 2015), 16) 0.49±0.06 (Schmidt et al., 2014), 17) 0.16±0.09 (Valente et al., 2015), 18) 0.19±0.04 (Valente et al., 2017).


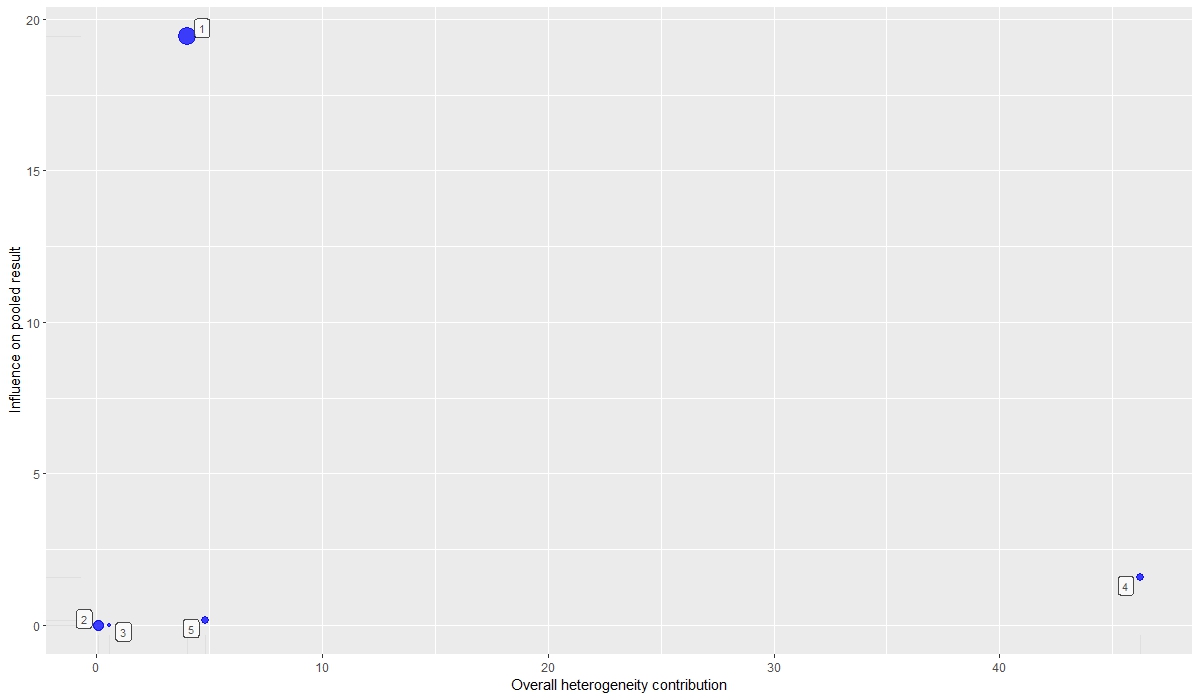


**Supplementary Figure S10.** Baujat plot from the meta-analysis of cow’s aggressiveness at calving. The code of the estimates were as follows: 1) 0.06±0.01 (Buddenberg et al., 1986), 2) 0.09±0.03 (Morris et al., 1994), 3) 0.14±0.08 (Hoppe et al., 2008), 4) 0.42±0.05 (Hoppe et al., 2008), and 5) 0.19±0.05 (Vallée et al., 2015).

**
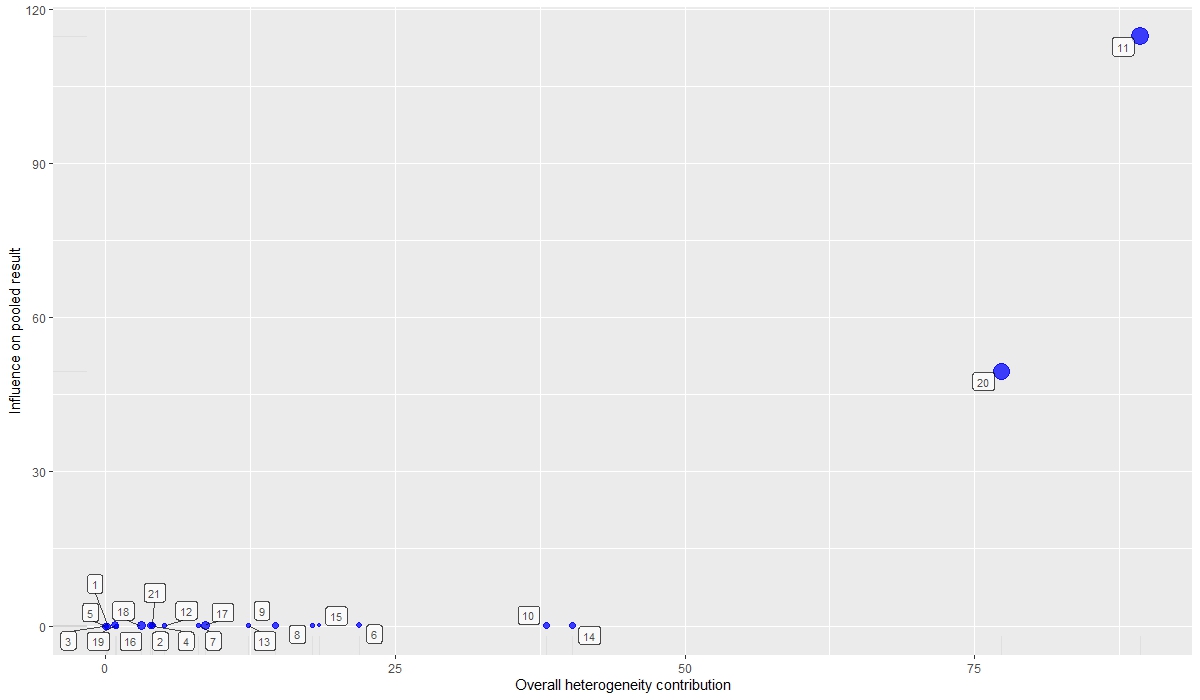
**

**Supplementary Figure S11.** Baujat plot from the meta-analysis of milking and general temperament scores. The code of the estimates were as follows: 1) 0.07±0.02 (Thompson et al., 1981), 2) 0.04±0.02 (Smith et al., 1985), 3) 0.08±0.02 (Foster et al., 1988), 4) 0.12±0.02 (Lawstuen et al., 1988), 5) 0.10±0.08 (Erf et al., 1992), 6) 0.22±0.03 (Visscher and Goddard, 1995), 7) 0.25±0.06 (Visscher and Goddard, 1995), 8) 0.33±0.06 (Cue et al., 1996), 9) 0.14±0.02 (Cue et al., 1996), 10) 0.17±0.02 (Cue et al., 1996), 11) 0.07±0.01 (Pryce et al., 2000), 12) 0.17±0.04 (Lassen and Mark, 2008), 13) 0.22±0.04 (Lassen and Mark, 2008), 14) 0.20±0.02 (Sewalem et al., 2011), 15) 0.38±0.07 (Kramer et al., 2013), 16) 0.04±0.04 (Kramer et al., 2013), 17) 0.10±0.01 (Stephansen et al., 2018), 18) 0.09±0.01 (Wethal et al., 2020), 19) 0.07±0.01 (Oliveira et al., 2021), 20) 0.09±0.01 (Szymik et al., 2021), 21) 0.14±0.03 (Taborda et al., 2023).

**
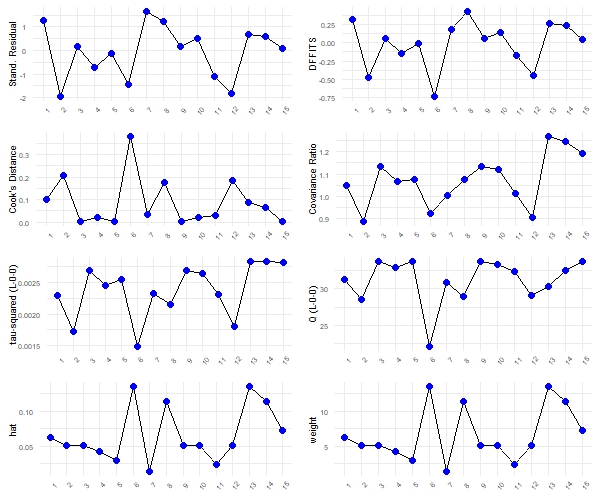
**

**Supplementary Figure S12.** The diagnostic plot in the meta-analysis of flight speed ax flight score. The code of the estimates (studies in x-axis) were as follows: 1) 0.36±0.06 (Hoppe et al., 2010), 2) 0.11±0.07 (Hoppe et al., 2010), 3) 0.28±0.07 (Hoppe et al., 2010), 4) 0.20±0.08 (Hoppe et al., 2010), 5) 0.25±0.10 (Hoppe et al., 2010), 6) 0.21±0.02 (Kadel et al., 2006), 7) 0.54±0.16 (Burrow et al., 1988), 8) 0.33±0.03 (Copley et al., 2022), 9) 0.28±0.07 (Corbet et al., 2013), 10) 0.31±0.07 (Corbet et al., 2013), 11) 0.13±0.12 (Halloway and Johnston, 2003), 12) 0.12±0.07 (Hine et al., 2019), 13) 0.30±0.02 (Kadel et al., 2006), 14) 0.30±0.03 (Littlejohn et al., 2018), 15) 0.27±0.05 (Schmidt et al., 2014)


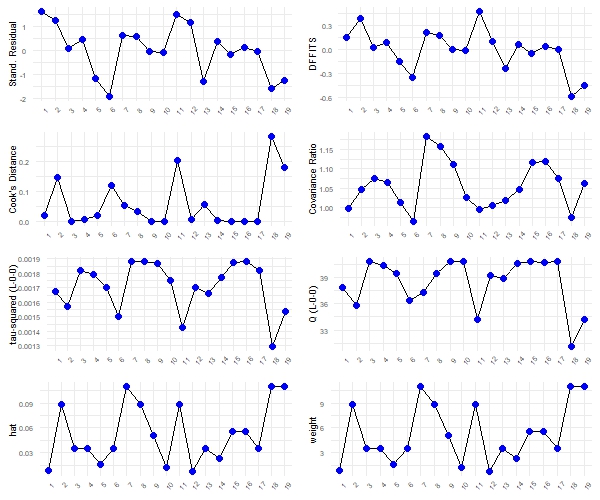


**Supplementary Figure S13.** The diagnostic plot in the meta-analysis of flight speed at weaning x yearling. The code of the estimates (studies in x-axis) were as follows: 1) 0.54±0.16 (Burrow et al., 1988), 2) 0.33±0.03 (Copley et al., 2022), 3) 0.28±0.07 (Corbet et al., 2013), 4) 0.31±0.07 (Corbet et al., 2013), 5) 0.13±0.12 (Halloway and Johnston, 2003), 6) 0.12±0.07 (Hine et al., 2019), 7) 0.30±0.02 (Kadel et al., 2006), 8) 0.30±0.03 (Littlejohn et al., 2018), 9) 0.27±0.05 (Schmidt et al., 2014), 10) 0.26±0.13 (Burrow et al., 1988), 11) 0.34±0.03 (Kadel et al., 2006), 12) 0.49±0.18 (Nkrumah et al., 2007), 13) 0.17±0.07 (Prayaga et al., 2009), 14) 0.31±0.09 (Prayaga et al., 2009), 15) 0.26±0.05 (Sant’Anna et al., 2012), 16) 0.28±0.05 (Sant’Anna et al., 2015), 17) 0.27±0.07 (Valente et al., 2015), 18) 0.21±0.02 (Valente et al., 2016), 19) 0.22±0.02 (Valente et al., 2017).


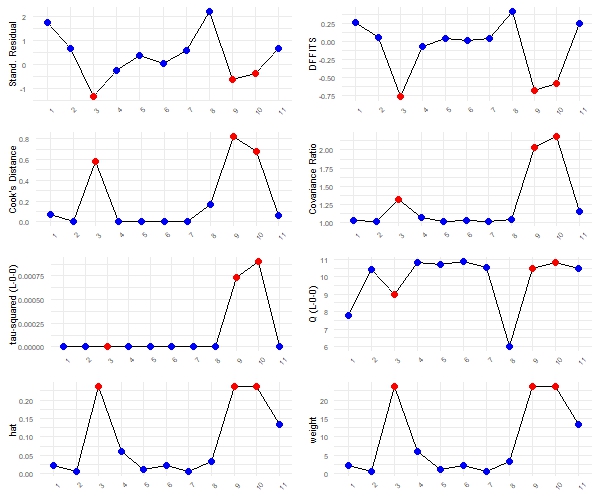


**Supplementary Figure S14.** The diagnostic plot in the meta-analysis of movement score. The code of the estimates (studies in x-axis) were as follows: 1) 0.29±0.10 (Benhajali et al., 2010), 2) 0.25±0.20 (Fordyce et al., 1982), 3) 0.08±0.03 (Freitas et al., 2023), 4) 0.10±0.06 (Hine et al., 2019), 5) 0.17±0.14 (Morris et al., 1994), 6) 0.12±0.10 (Morris et al., 1994), 7) 0.24±0.22 (Morris et al., 1994), 8) 0.29±0.08 (Peixoto et al., 2016), 9) 0.10±0.03 (Sant’Anna et al., 2015), 10) 0.11±0.03 (Valente et al., 2015), and 11) 0.14±0.04 (Valente et al., 2017).

**
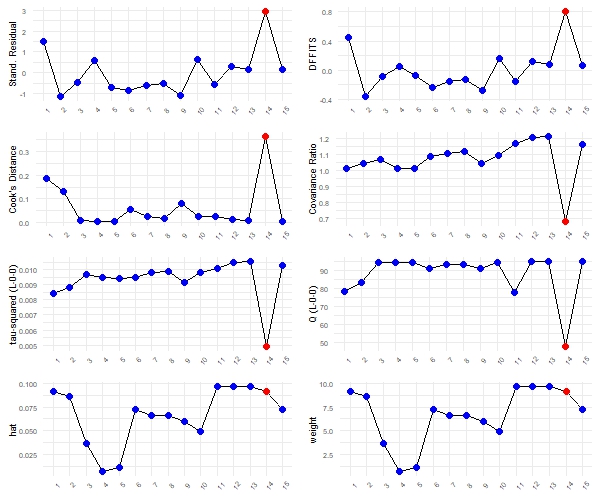
**

**Supplementary Figure S15.** The diagnostic plot in the meta-analysis of crush score at weaning. The code of the estimates (studies in x-axis) were as follows: 1) 0.38±0.03 (Beckman et al., 2007), 2) 0.13±0.04 **(**Celestino et al., 2019), 3) 0.17±0.13 (Halloway and Johnston, 2003), 4) 0.46±0.37 (Hearnshaw and Morris, 1984), 5) 0.03±0.28 (Hearnshaw and Morris, 1984), 6) 0.15±0.06 (Hoppe et al., 2010), 7) 0.17±0.07 (Hoppe et al., 2010), 8) 0.18±0.07 (Hoppe et al., 2010), 9) 0.11±0.08 (Hoppe et al., 2010), 10) 0.33±0.10 (Hoppe et al., 2010), 11) 0.19±0.02 (Kadel et al., 2006), 12) 0.27±0.02 (Torres-Vázquez and Spangler, 2016), 13) 0.26±0.02 (Walkon et al., 2018), 14) 0.46±0.03 (Walkon et al., 2018), and 15) 0.26±0.06 (Yu et al., 2020).

**
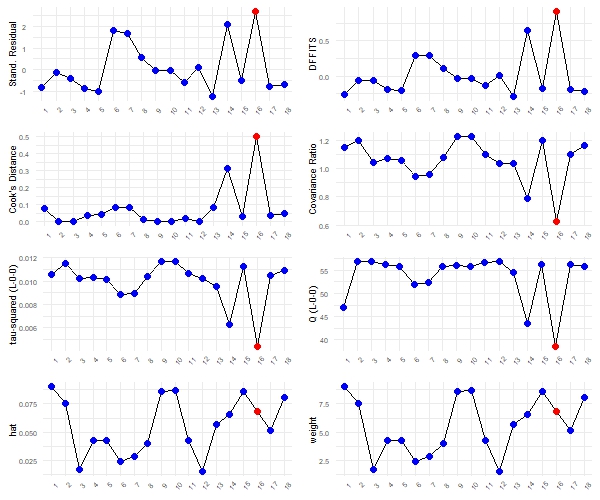
**

**Supplementary Figure S16.** The diagnostic plot in the meta-analysis of pen score at weaning x yearling. The code of the estimates (studies in x-axis) were as follows: 1) 0.18±0.02 (Barrozo et al., 2012), 2) 0.25±0.05 (Celestino et al., 2019), 3) 0.17±0.21 (Fordyce et al., 1982), 4) 0.14±0.11 (Fordyce et al., 1996), 5) 0.12±0.11 (Fordyce et al., 1996), 6) 0.61±0.17 (Gauly et al., 2001), 7) 0.55±0.15 (Gauly et al., 2001), 8) 0.35±0.12 (Hanna et al., 2014), 9) 0.26±0.03 (Littlejohn et al., 2018), 10) 0.26±0.03 (Lucena et al., 2014), 11) 0.18±0.11 (Morris et al., 1994), 12) 0.29±0.23 (Morris et al., 1994), 13) 0.11±0.08 (Neves et al., 2014), 14) 0.47±0.07 (Riley et al., 2014), 15) 0.21±0.03 (Sant’Anna et al., 2015), 16) 0.49±0.06 (Schmidt et al., 2014), 17) 0.16±0.09 (Valente et al., 2015), 18) 0.19±0.04 (Valente et al., 2017)


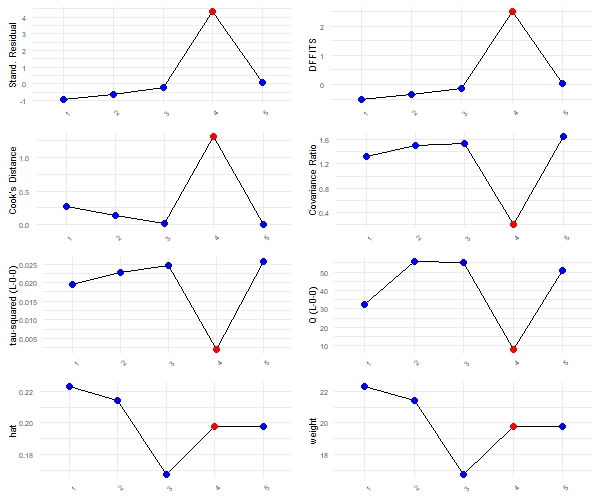


**Supplementary Figure S17.** The diagnostic plot in the meta-analysis of cow’s aggressiveness at calving. The code of the estimates (studies in x-axis) were as follows: 1) 0.06±0.01 (Buddenberg et al., 1986), 2) 0.09±0.03 (Morris et al., 1994), 3) 0.14±0.08 (Hoppe et al., 2008), 4) 0.42±0.05 (Hoppe et al., 2008), and 5) 0.19±0.05 (Vallée et al., 2015)


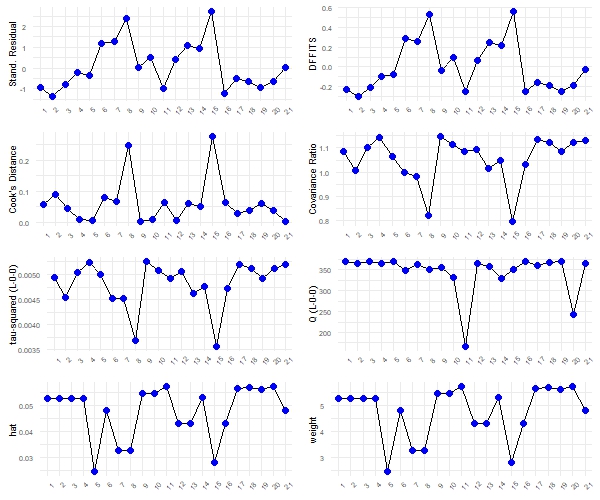


**Supplementary Figure S18.** The diagnostic plot in the meta-analysis of milking and general temperament scores. The code of the estimates (studies in x-axis) were as follows: 1) 0.07±0.02 (Thompson et al., 1981), 2) 0.04±0.02 (Smith et al., 1985), 3) 0.08±0.02 (Foster et al., 1988), 4) 0.12±0.02 (Lawstuen et al., 1988), 5) 0.10±0.08 (Erf et al., 1992), 6) 0.22±0.03 (Visscher and Goddard, 1995), 7) 0.25±0.06 (Visscher and Goddard, 1995), 8) 0.33±0.06 (Cue et al., 1996), 9) 0.14±0.02 (Cue et al., 1996), 10) 0.17±0.02 (Cue et al., 1996), 11) 0.07±0.01 (Pryce et al., 2000), 12) 0.17±0.04 (Lassen and Mark, 2008), 13) 0.22±0.04 (Lassen and Mark, 2008), 14) 0.20±0.02 (Sewalem et al., 2011), 15) 0.38±0.07 (Kramer et al., 2013), 16) 0.04±0.04 (Kramer et al., 2013), 17) 0.10±0.01 (Stephansen et al., 2018), 18) 0.09±0.01 (Wethal et al., 2020), 19) 0.07±0.01 (Oliveira et al., 2021), 20) 0.09±0.01 (Szymik et al., 2021), 21) 0.14±0.03 (Taborda et al., 2023).


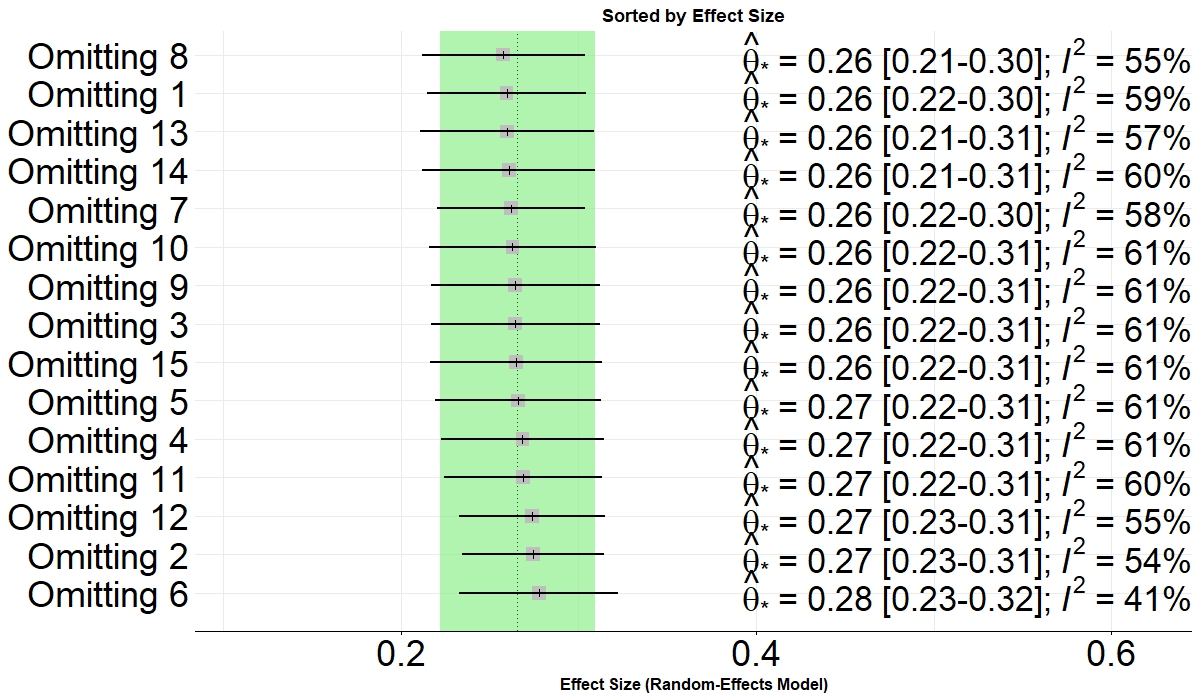

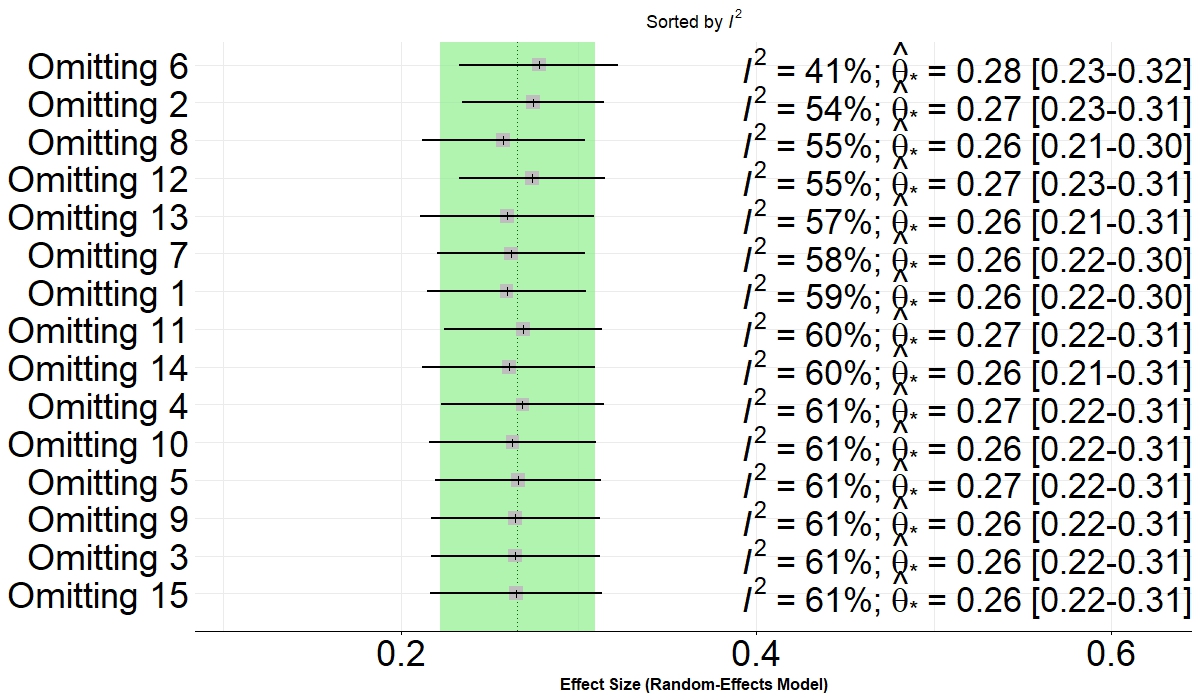


**Supplementary Figure S19.** Effect Size and Heterogeneity index (I^2^) modification when a study is leaving out in meta-analysis of flight speed ax flight score. The code of the estimates omitted were as follows: 1) 0.36±0.06 (Hoppe et al., 2010), 2) 0.11±0.07 (Hoppe et al., 2010), 3) 0.28±0.07 (Hoppe et al., 2010), 4) 0.20±0.08 (Hoppe et al., 2010), 5) 0.25±0.10 (Hoppe et al., 2010), 6) 0.21±0.02 (Kadel et al., 2006), 7) 0.54±0.16 (Burrow et al., 1988), 8) 0.33±0.03 (Copley et al., 2022), 9) 0.28±0.07 (Corbet et al., 2013), 10) 0.31±0.07 (Corbet et al., 2013), 11) 0.13±0.12 (Halloway and Johnston, 2003), 12) 0.12±0.07 (Hine et al., 2019), 13) 0.30±0.02 (Kadel et al., 2006), 14) 0.30±0.03 (Littlejohn et al., 2018), 15) 0.27±0.05 (Schmidt et al., 2014)


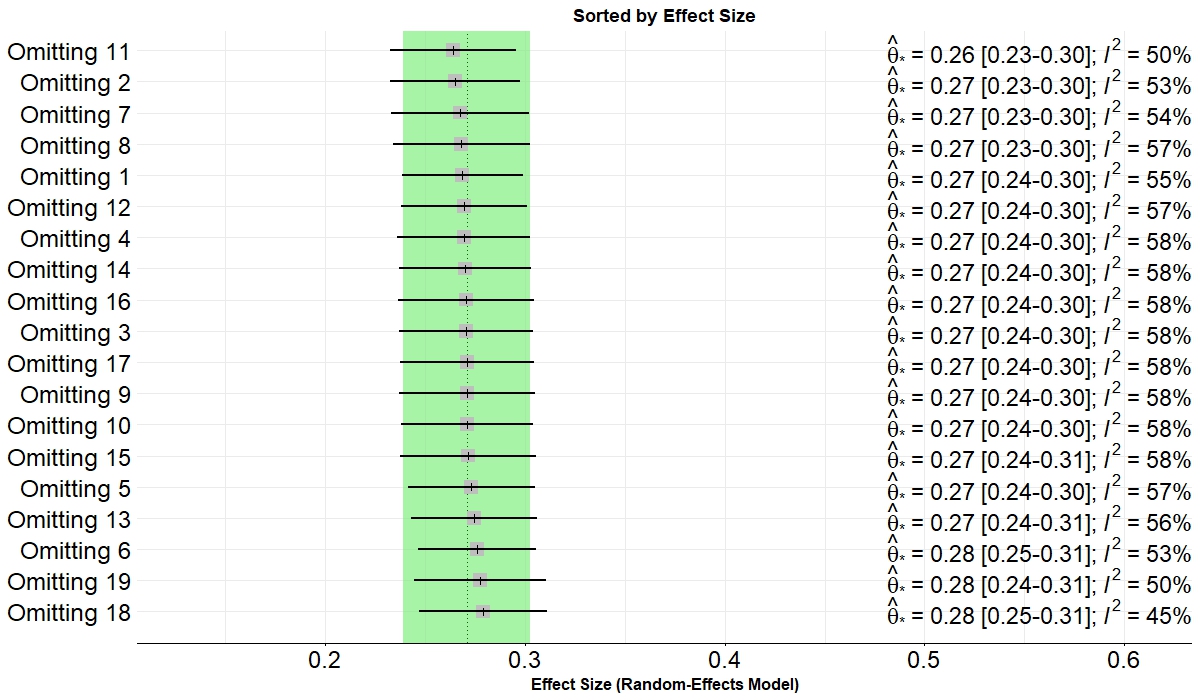

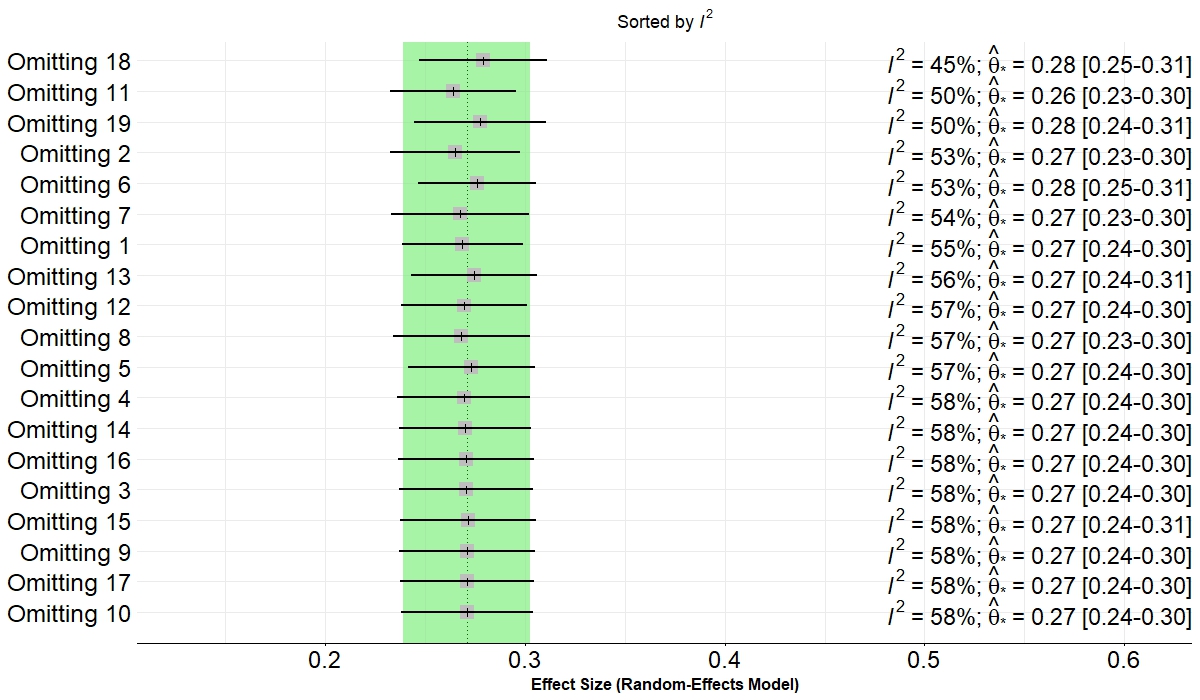


**Supplementary Figure S20.** Effect Size and Heterogeneity index (I^2^) modification when a study is leaving out in meta-analysis of flight speed at weaning x yearling. The code of the estimates omitted were as follows: 1) 0.54±0.16 (Burrow et al., 1988), 2) 0.33±0.03 (Copley et al., 2022), 3) 0.28±0.07 (Corbet et al., 2013), 4) 0.31±0.07 (Corbet et al., 2013), 5) 0.13±0.12 (Halloway and Johnston, 2003), 6) 0.12±0.07 (Hine et al., 2019), 7) 0.30±0.02 (Kadel et al., 2006), 8) 0.30±0.03 (Littlejohn et al., 2018), 9) 0.27±0.05 (Schmidt et al., 2014), 10) 0.26±0.13 (Burrow et al., 1988), 11) 0.34±0.03 (Kadel et al., 2006), 12) 0.49±0.18 (Nkrumah et al., 2007), 13) 0.17±0.07 (Prayaga et al., 2009), 14) 0.31±0.09 (Prayaga et al., 2009), 15) 0.26±0.05 (Sant’Anna et al., 2012), 16) 0.28±0.05 (Sant’Anna et al., 2015), 17) 0.27±0.07 (Valente et al., 2015), 18) 0.21±0.02 (Valente et al., 2016), 19) 0.22±0.02 (Valente et al., 2017).


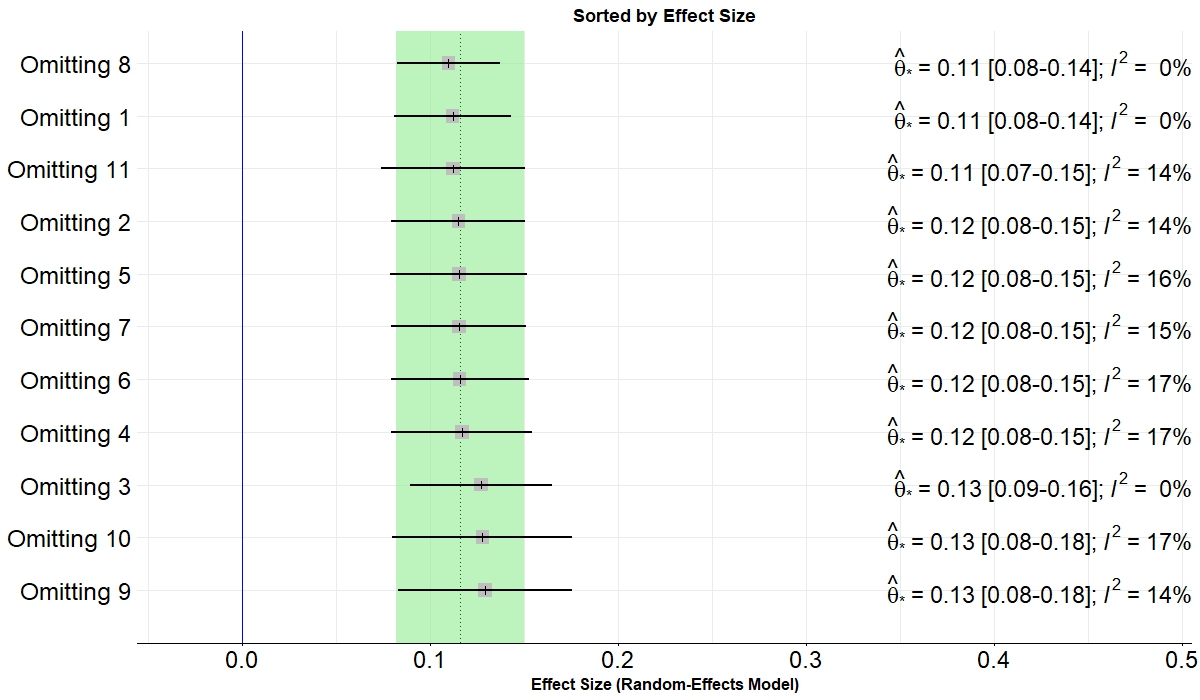

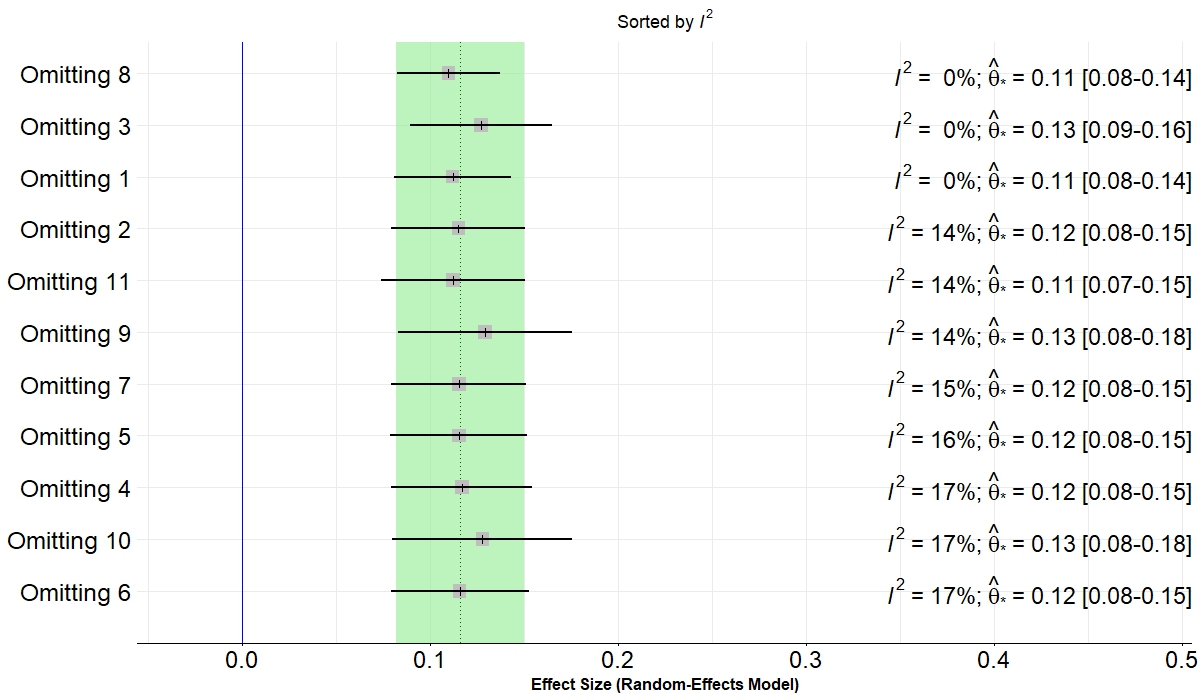


**Supplementary Figure S21.** Effect Size and Heterogeneity index (I^2^) modification when a study is leaving out in meta-analysis of movement score. The code of the estimates omitted were as follows: 1) 0.29±0.10 (Benhajali et al., 2010), 2) 0.25±0.20 (Fordyce et al., 1982), 3) 0.08±0.03 (Freitas et al., 2023), 4) 0.10±0.06 (Hine et al., 2019), 5) 0.17±0.14 (Morris et al., 1994), 6) 0.12±0.10 (Morris et al., 1994), 7) 0.24±0.22 (Morris et al., 1994), 8) 0.29±0.08 (Peixoto et al., 2016), 9) 0.10±0.03 (Sant’Anna et al., 2015), 10) 0.11±0.03 (Valente et al., 2015), and 11) 0.14±0.04 (Valente et al., 2017).


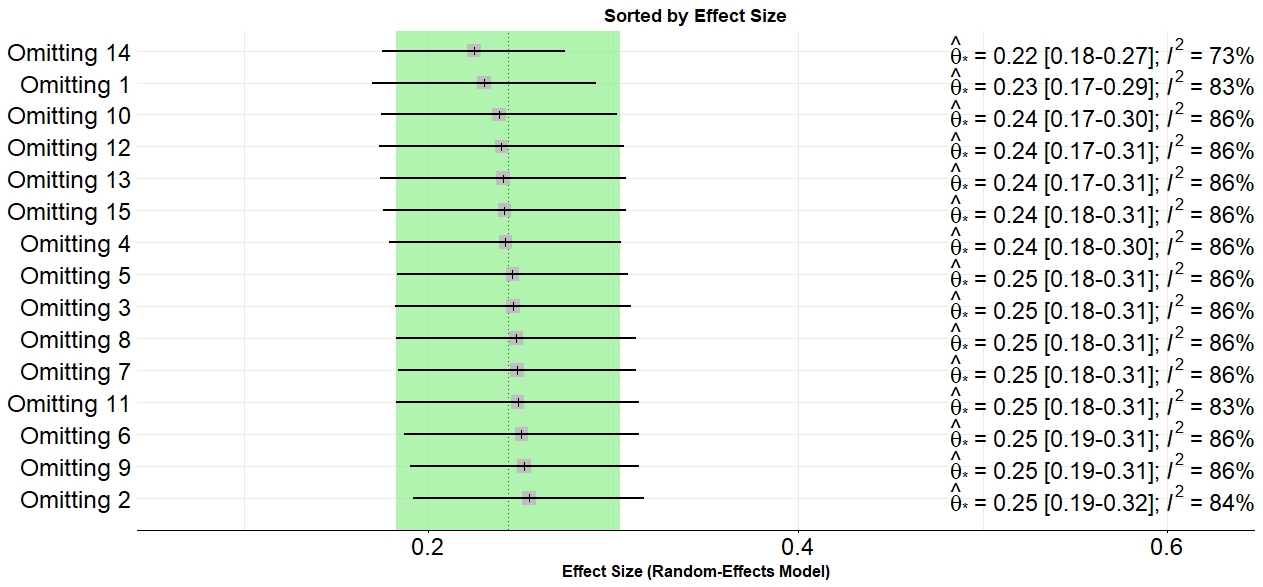


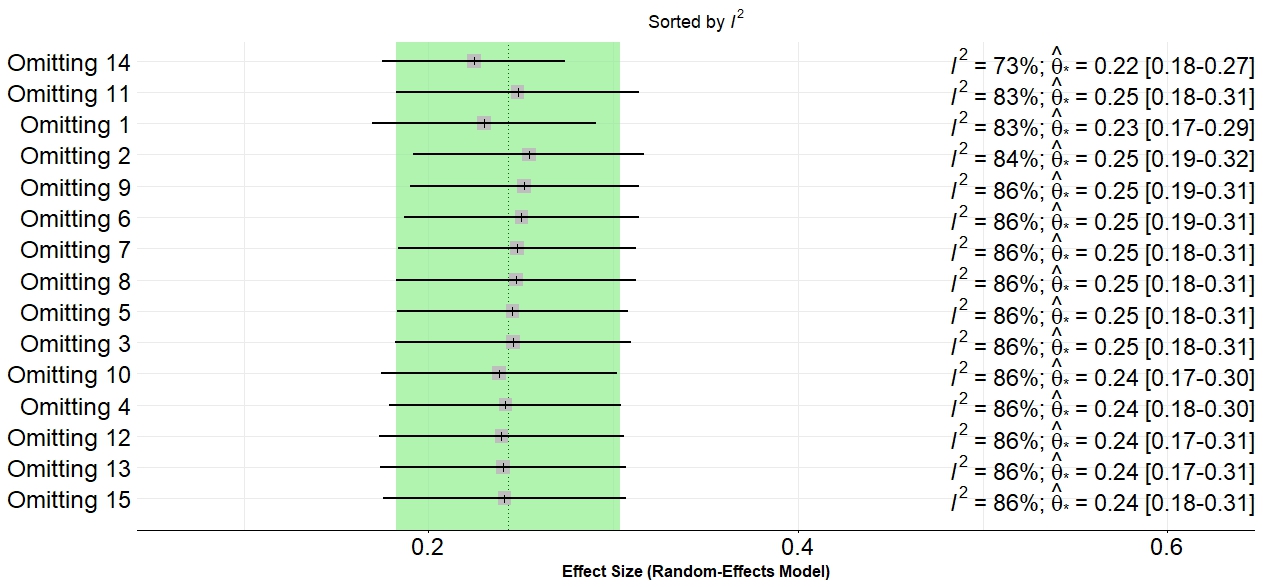


**Supplementary Figure S22.** Effect Size and Heterogeneity index (I^2^) modification when a study is leaving out in meta-analysis of crush score at weaning. The code of the estimates omitted were as follows: 1) 0.38±0.03 (Beckman et al., 2007), 2) 0.13±0.04 **(**Celestino et al., 2019), 3) 0.17±0.13 (Halloway and Johnston, 2003), 4) 0.46±0.37 (Hearnshaw and Morris, 1984), 5) 0.03±0.28 (Hearnshaw and Morris, 1984), 6) 0.15±0.06 (Hoppe et al., 2010), 7) 0.17±0.07 (Hoppe et al., 2010), 8) 0.18±0.07 (Hoppe et al., 2010), 9) 0.11±0.08 (Hoppe et al., 2010), 10) 0.33±0.10 (Hoppe et al., 2010), 11) 0.19±0.02 (Kadel et al., 2006), 12) 0.27±0.02 (Torres-Vázquez and Spangler, 2016), 13) 0.26±0.02 (Walkon et al., 2018), 14) 0.46±0.03 (Walkon et al., 2018), and 15) 0.26±0.06 (Yu et al., 2020).


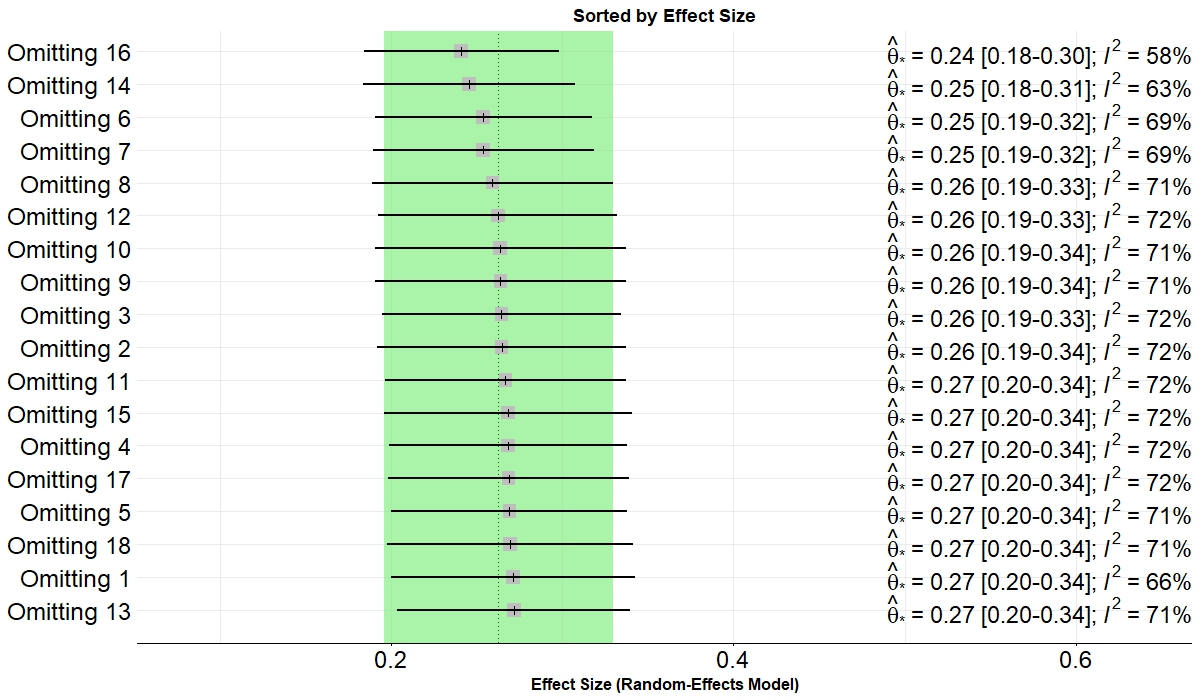

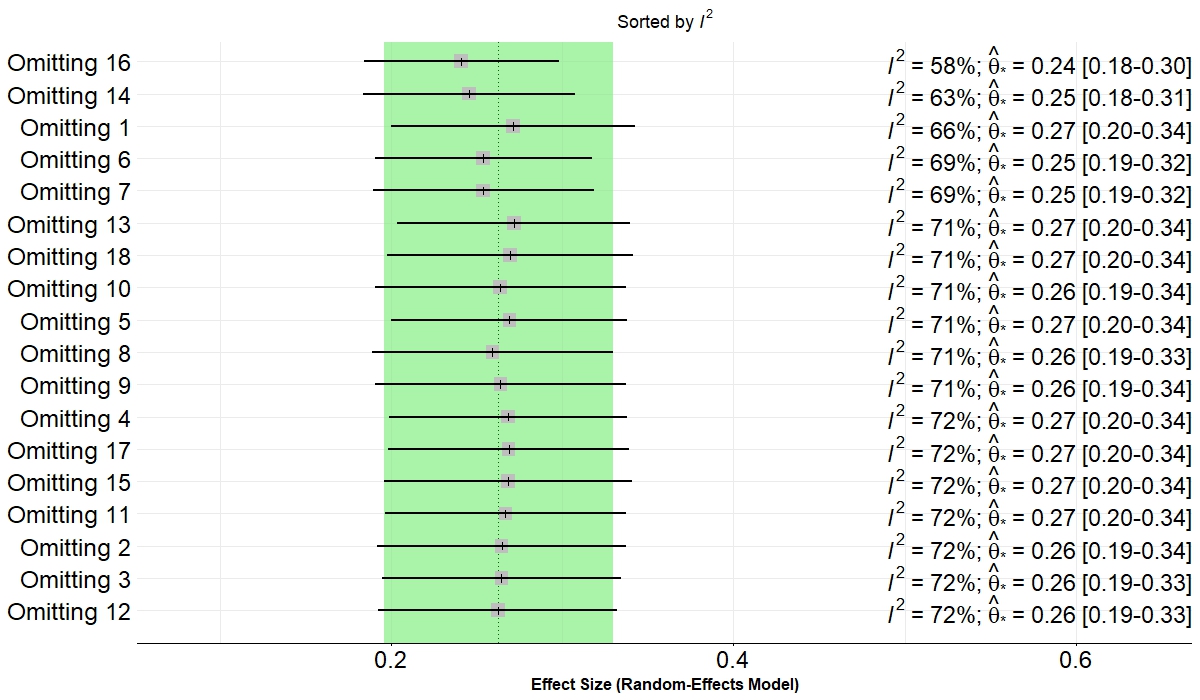


**Supplementary Figure S23.** Effect Size and Heterogeneity index (I^2^) modification when a study is leaving out in meta-analysis of pen score at weaning x yearling. The code of the estimates omitted were as follows: 1) 0.18±0.02 (Barrozo et al., 2012), 2) 0.25±0.05 (Celestino et al., 2019), 3) 0.17±0.21 (Fordyce et al., 1982), 4) 0.14±0.11 (Fordyce et al., 1996), 5) 0.12±0.11 (Fordyce et al., 1996), 6) 0.61±0.17 (Gauly et al., 2001), 7) 0.55±0.15 (Gauly et al., 2001), 8) 0.35±0.12 (Hanna et al., 2014), 9) 0.26±0.03 (Littlejohn et al., 2018), 10) 0.26±0.03 (Lucena et al., 2014), 11) 0.18±0.11 (Morris et al., 1994), 12) 0.29±0.23 (Morris et al., 1994), 13) 0.11±0.08 (Neves et al., 2014), 14) 0.47±0.07 (Riley et al., 2014), 15) 0.21±0.03 (Sant’Anna et al., 2015), 16) 0.49±0.06 (Schmidt et al., 2014), 17) 0.16±0.09 (Valente et al., 2015), 18) 0.19±0.04 (Valente et al., 2017).


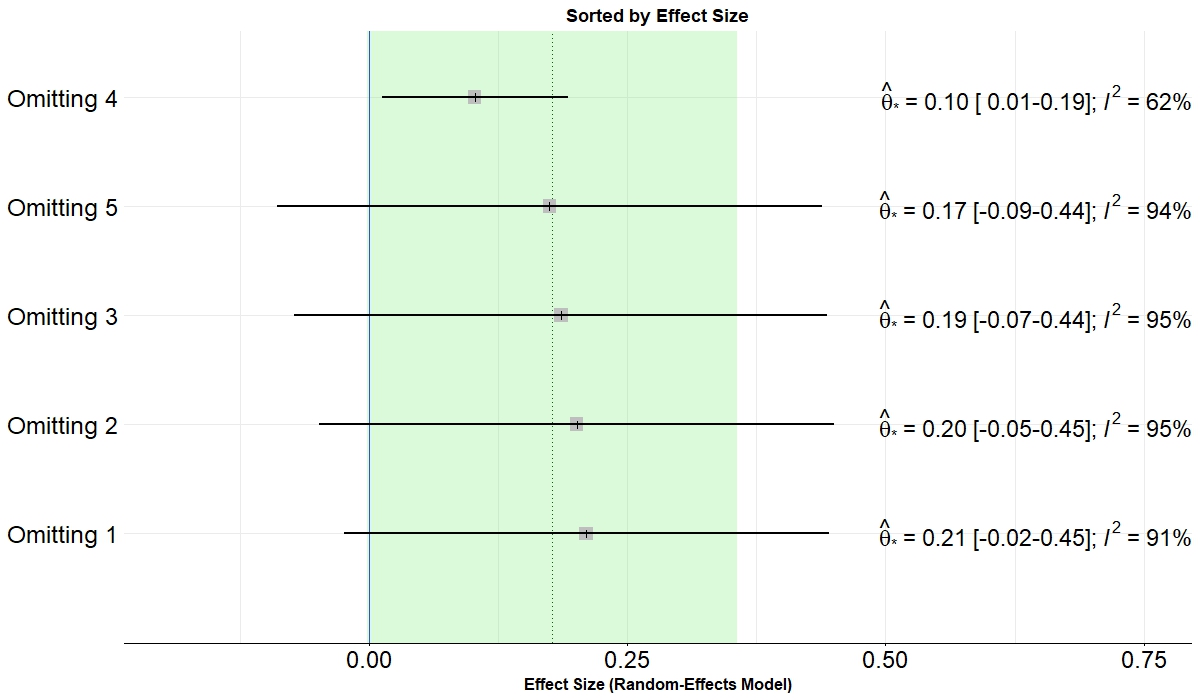

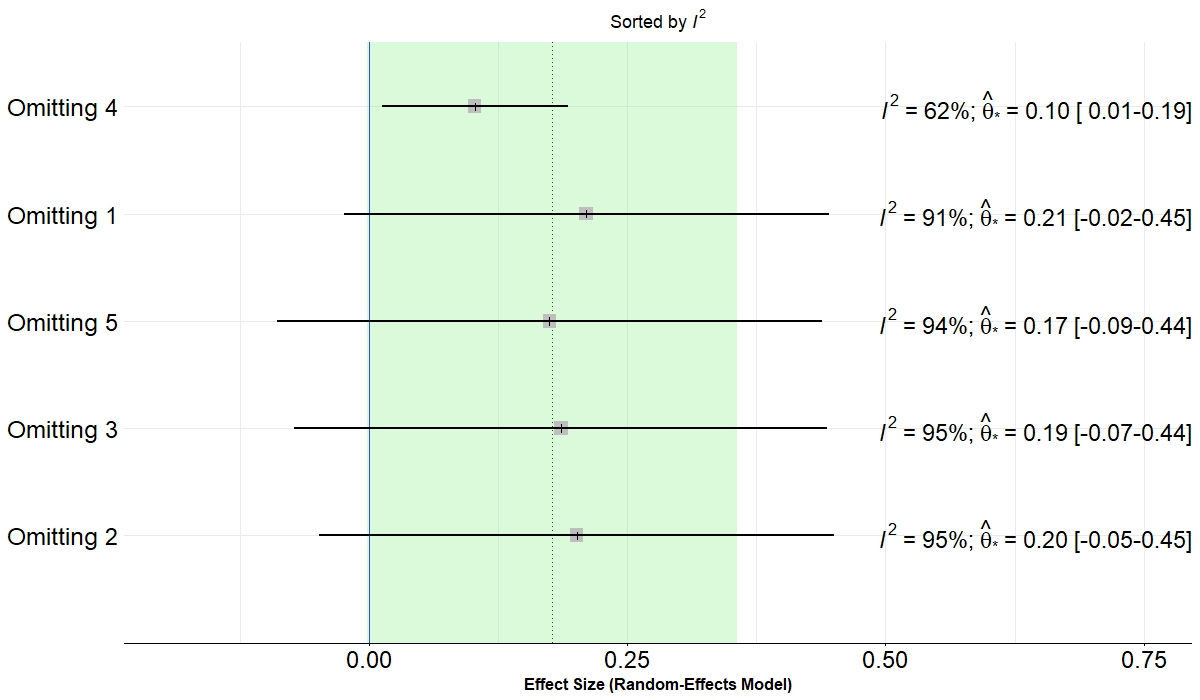


**Supplementary Figure S24.** Effect Size and Heterogeneity index (I^2^) modification when a study is leaving out in meta-analysis of cow’s aggressiveness at calving. The code of the estimates omitted were as follows: 1) 0.06±0.01 (Buddenberg et al., 1986), 2) 0.09±0.03 (Morris et al., 1994), 3) 0.14±0.08 (Hoppe et al., 2008), 4) 0.42±0.05 (Hoppe et al., 2008), and 5) 0.19±0.05 (Vallée et al., 2015)

**
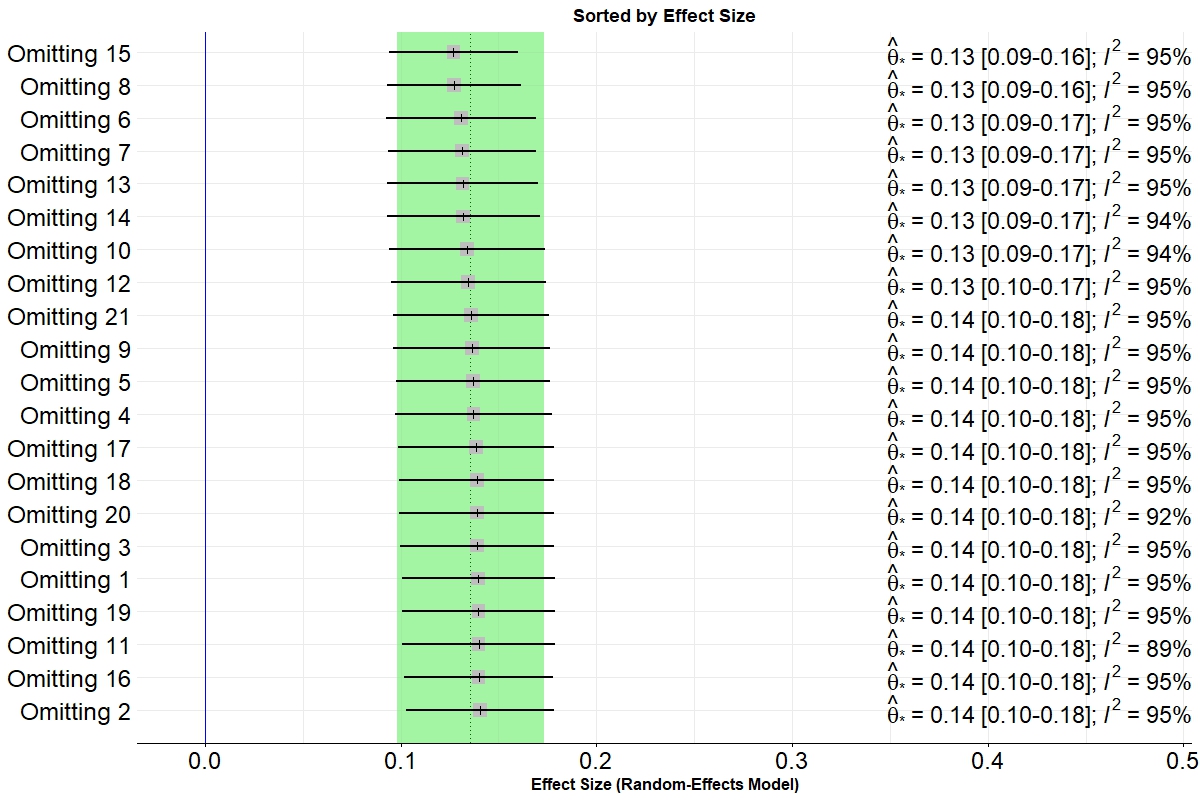

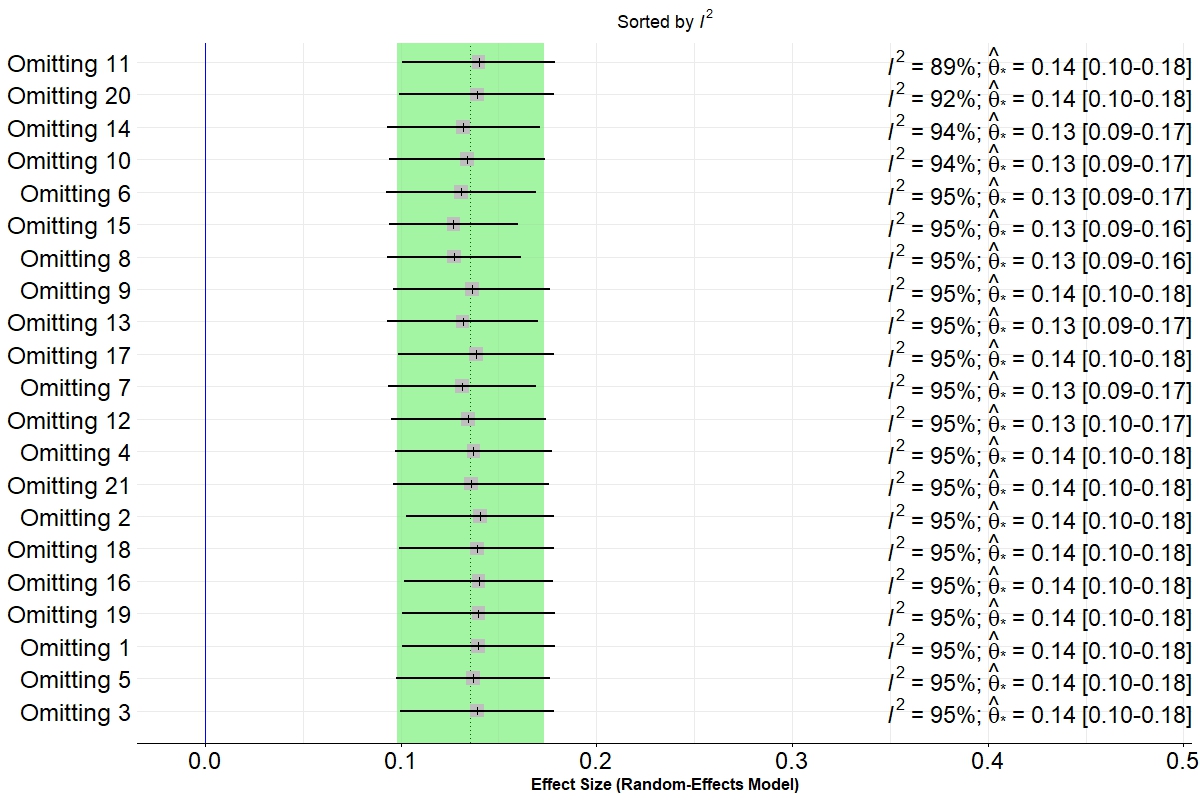
**

**Supplementary Figure S25.** Effect Size and Heterogeneity index (I^2^) modification when a study is leaving out in meta-analysis of milking and general temperament scores. The code of the estimates omitted were as follows: 1) 0.07±0.02 (Thompson et al., 1981), 2) 0.04±0.02 (Smith et al., 1985), 3) 0.08±0.02 (Foster et al., 1988), 4) 0.12±0.02 (Lawstuen et al., 1988), 5) 0.10±0.08 (Erf et al., 1992), 6) 0.22±0.03 (Visscher and Goddard, 1995), 7) 0.25±0.06 (Visscher and Goddard, 1995), 8) 0.33±0.06 (Cue et al., 1996), 9) 0.14±0.02 (Cue et al., 1996), 10) 0.17±0.02 (Cue et al., 1996), 11) 0.07±0.01 (Pryce et al., 2000), 12) 0.17±0.04 (Lassen and Mark, 2008), 13) 0.22±0.04 (Lassen and Mark, 2008), 14) 0.20±0.02 (Sewalem et al., 2011), 15) 0.38±0.07 (Kramer et al., 2013), 16) 0.04±0.04 (Kramer et al., 2013), 17) 0.10±0.01 (Stephansen et al., 2018), 18) 0.09±0.01 (Wethal et al., 2020), 19) 0.07±0.01 (Oliveira et al., 2021), 20) 0.09±0.01 (Szymik et al., 2021), 21) 0.14±0.03 (Taborda et al., 2023).

**
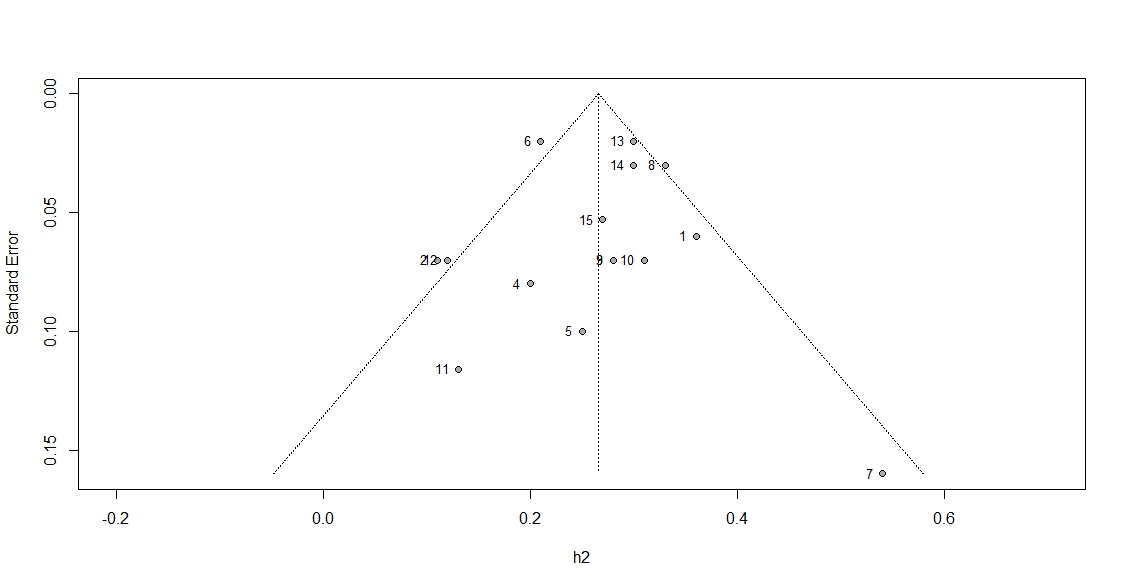
**

**Supplementary Figure S26.** Funnel plot in the meta-analysis of flight speed ax flight score. The code of the estimates were as follows: 1) 0.36±0.06 (Hoppe et al., 2010), 2) 0.11±0.07 (Hoppe et al., 2010), 3) 0.28±0.07 (Hoppe et al., 2010), 4) 0.20±0.08 (Hoppe et al., 2010), 5) 0.25±0.10 (Hoppe et al., 2010), 6) 0.21±0.02 (Kadel et al., 2006), 7) 0.54±0.16 (Burrow et al., 1988), 8) 0.33±0.03 (Copley et al., 2022), 9) 0.28±0.07 (Corbet et al., 2013), 10) 0.31±0.07 (Corbet et al., 2013), 11) 0.13±0.12 (Halloway and Johnston, 2003), 12) 0.12±0.07 (Hine et al., 2019), 13) 0.30±0.02 (Kadel et al., 2006), 14) 0.30±0.03 (Littlejohn et al., 2018), and 15) 0.27±0.05 (Schmidt et al., 2014).


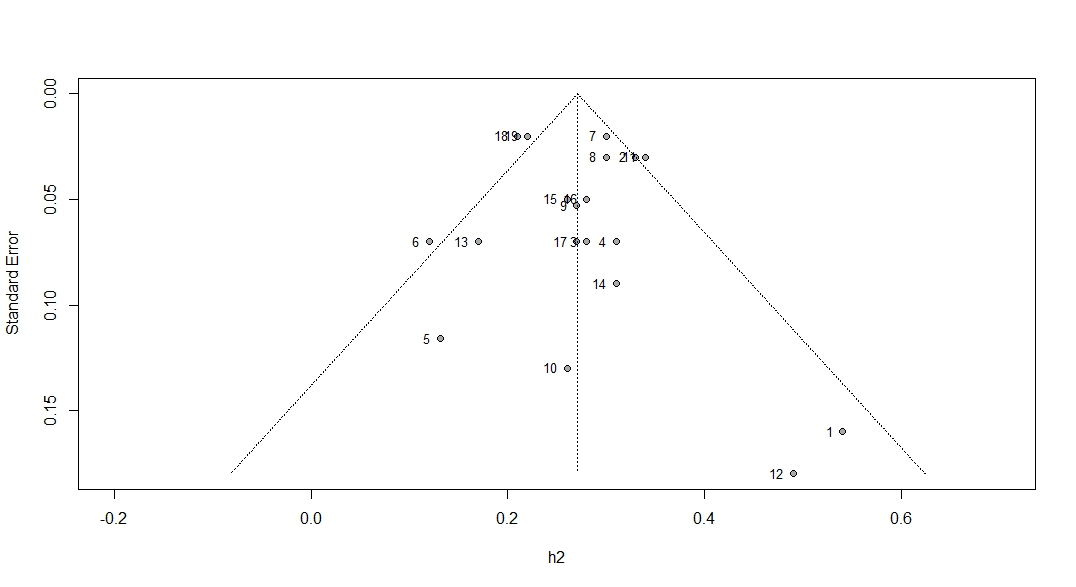


**Supplementary Figure S27.** The funnel plot in the meta-analysis of flight speed at weaning x yearling. The code of the estimates were as follows: 1) 0.54±0.16 (Burrow et al., 1988), 2) 0.33±0.03 (Copley et al., 2022), 3) 0.28±0.07 (Corbet et al., 2013), 4) 0.31±0.07 (Corbet et al., 2013), 5) 0.13±0.12 (Halloway and Johnston, 2003), 6) 0.12±0.07 (Hine et al., 2019), 7) 0.30±0.02 (Kadel et al., 2006), 8) 0.30±0.03 (Littlejohn et al., 2018), 9) 0.27±0.05 (Schmidt et al., 2014), 10) 0.26±0.13 (Burrow et al., 1988), 11) 0.34±0.03 (Kadel et al., 2006), 12) 0.49±0.18 (Nkrumah et al., 2007), 13) 0.17±0.07 (Prayaga et al., 2009), 14) 0.31±0.09 (Prayaga et al., 2009), 15) 0.26±0.05 (Sant’Anna et al., 2012), 16) 0.28±0.05 (Sant’Anna et al., 2015), 17) 0.27±0.07 (Valente et al., 2015), 18) 0.21±0.02 (Valente et al., 2016), 19) 0.22±0.02 (Valente et al., 2017).


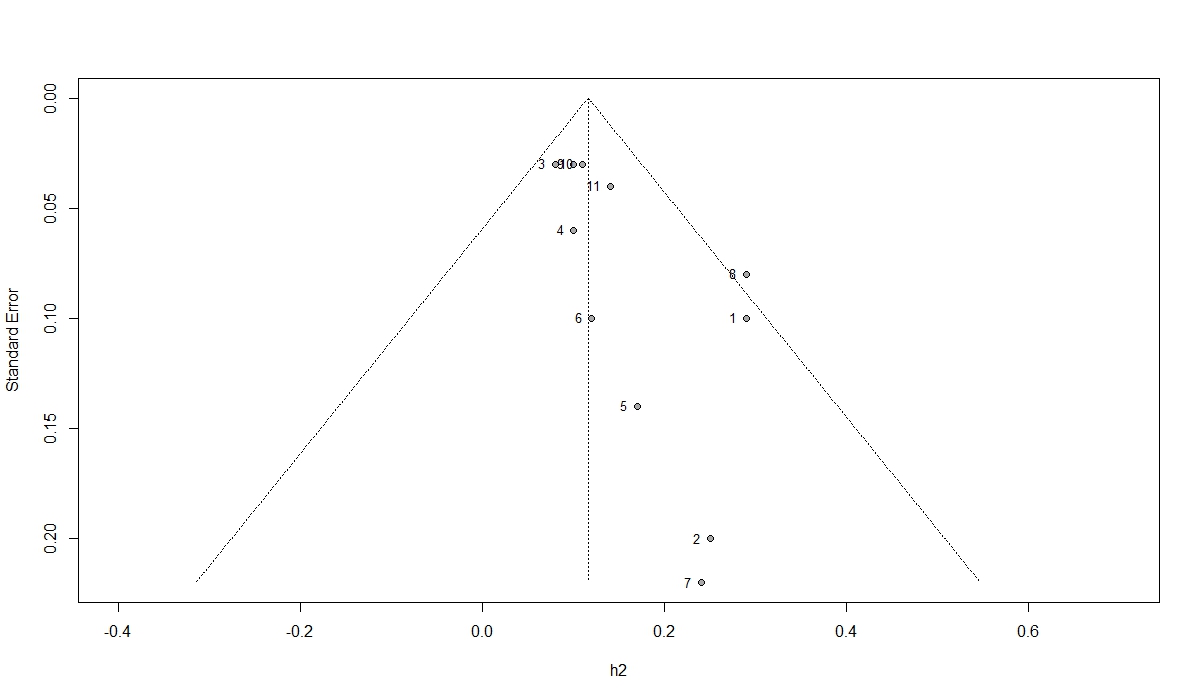


**Supplementary Figure S28.** The diagnostic plot in the meta-analysis of movement score. The code of the estimates were as follows: 1) 0.29±0.10 (Benhajali et al., 2010), 2) 0.25±0.20 (Fordyce et al., 1982), 3) 0.08±0.03 (Freitas et al., 2023), 4) 0.10±0.06 (Hine et al., 2019), 5) 0.17±0.14 (Morris et al., 1994), 6) 0.12±0.10 (Morris et al., 1994), 7) 0.24±0.22 (Morris et al., 1994), 8) 0.29±0.08 (Peixoto et al., 2016), 9) 0.10±0.03 (Sant’Anna et al., 2015), 10) 0.11±0.03 (Valente et al., 2015), and 11) 0.14±0.04 (Valente et al., 2017).

**
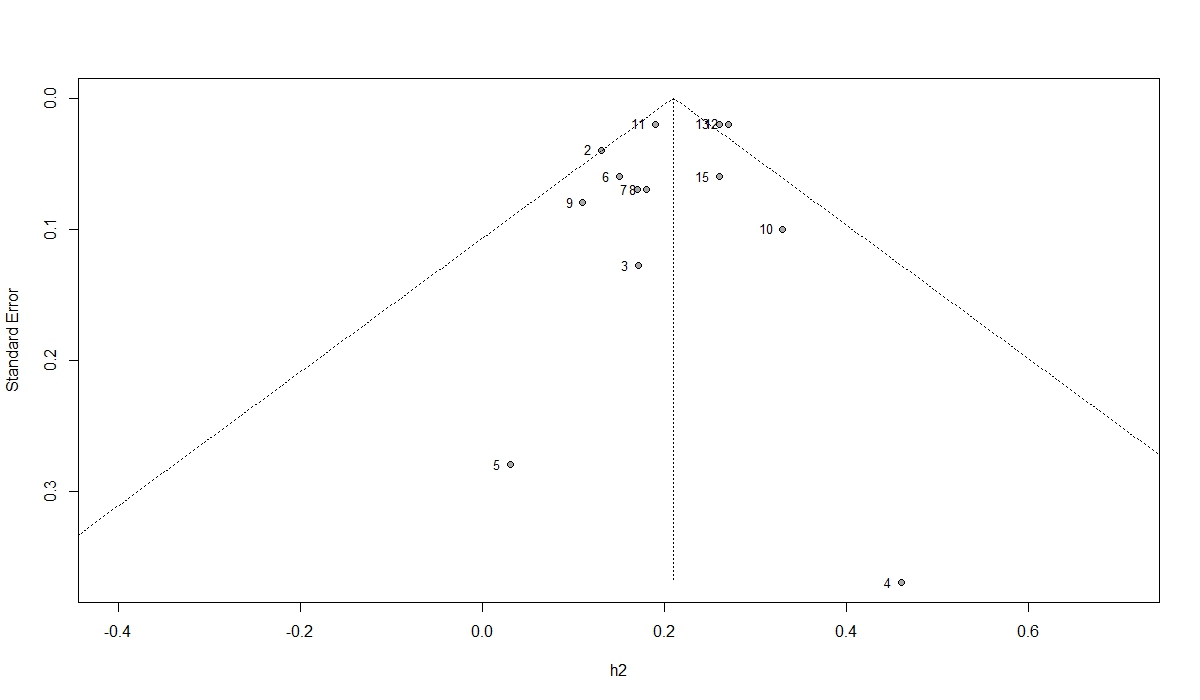
**

**Supplementary Figure S29.** The funnel plot in the meta-analysis of crush score at weaning. The code of the estimates were as follows: 1) 0.38±0.03 (Beckman et al., 2007), 2) 0.13±0.04 **(**Celestino et al., 2019), 3) 0.17±0.13 (Halloway and Johnston, 2003), 4) 0.46±0.37 (Hearnshaw and Morris, 1984), 5) 0.03±0.28 (Hearnshaw and Morris, 1984), 6) 0.15±0.06 (Hoppe et al., 2010), 7) 0.17±0.07 (Hoppe et al., 2010), 8) 0.18±0.07 (Hoppe et al., 2010), 9) 0.11±0.08 (Hoppe et al., 2010), 10) 0.33±0.10 (Hoppe et al., 2010), 11) 0.19±0.02 (Kadel et al., 2006), 12) 0.27±0.02 (Torres-Vázquez and Spangler, 2016), 13) 0.26±0.02 (Walkon et al., 2018), 14) 0.46±0.03 (Walkon et al., 2018), and 15) 0.26±0.06 (Yu et al., 2020).

**
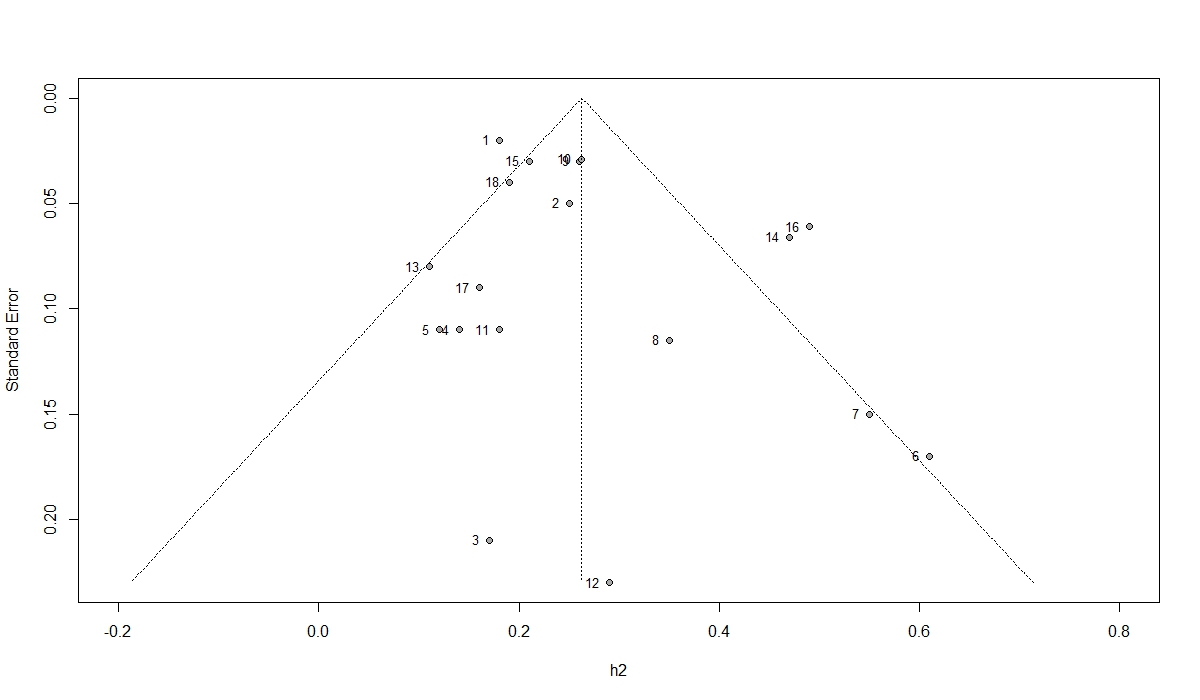
**

**Supplementary Figure S30.** The funnel plot in the meta-analysis of pen score at weaning x yearling. The code of the estimates were as follows: 1) 0.18±0.02 (Barrozo et al., 2012), 2) 0.25±0.05 (Celestino et al., 2019), 3) 0.17±0.21 (Fordyce et al., 1982), 4) 0.14±0.11 (Fordyce et al., 1996), 5) 0.12±0.11 (Fordyce et al., 1996), 6) 0.61±0.17 (Gauly et al., 2001), 7) 0.55±0.15 (Gauly et al., 2001), 8) 0.35±0.12 (Hanna et al., 2014), 9) 0.26±0.03 (Littlejohn et al., 2018), 10) 0.26±0.03 (Lucena et al., 2014), 11) 0.18±0.11 (Morris et al., 1994), 12) 0.29±0.23 (Morris et al., 1994), 13) 0.11±0.08 (Neves et al., 2014), 14) 0.47±0.07 (Riley et al., 2014), 15) 0.21±0.03 (Sant’Anna et al., 2015), 16) 0.49±0.06 (Schmidt et al., 2014), 17) 0.16±0.09 (Valente et al., 2015), 18) 0.19±0.04 (Valente et al., 2017).


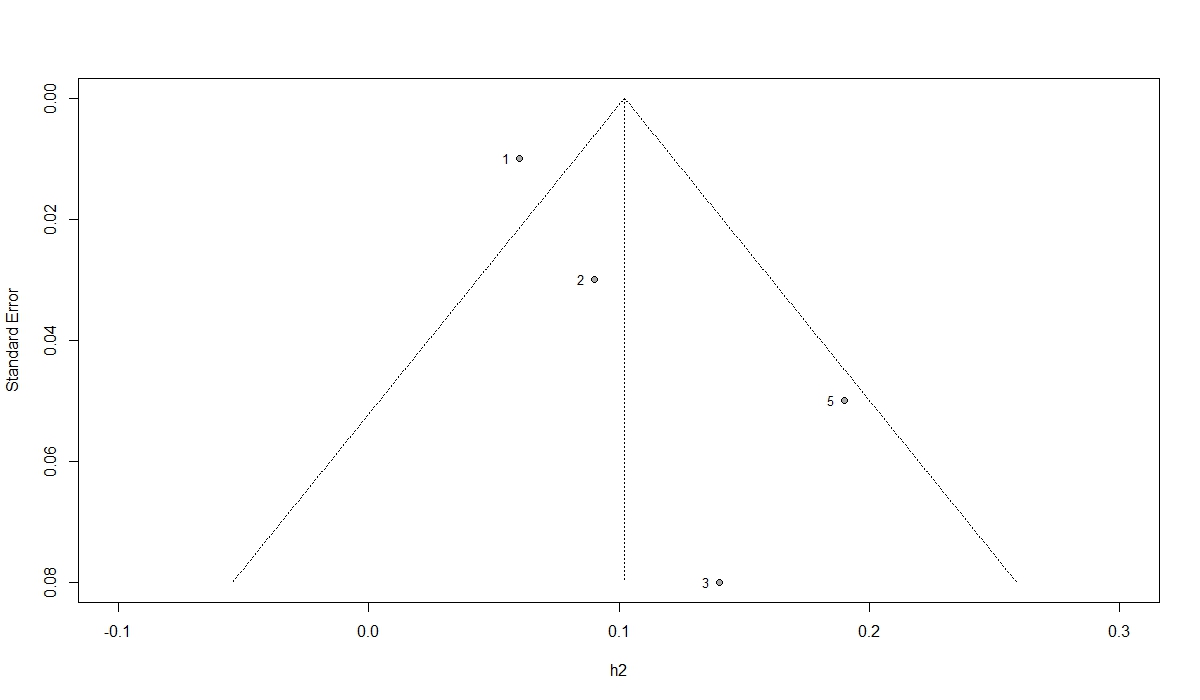


**Supplementary Figure S31.** The funnel plot in the meta-analysis of cow’s aggressiveness at calving. The code of the estimates were as follows: 1) 0.06±0.01 (Buddenberg et al., 1986), 2) 0.09±0.03 (Morris et al., 1994), 3) 0.14±0.08 (Hoppe et al., 2008), 4) 0.42±0.05 (Hoppe et al., 2008), and 5) 0.19±0.05 (Vallée et al., 2015).


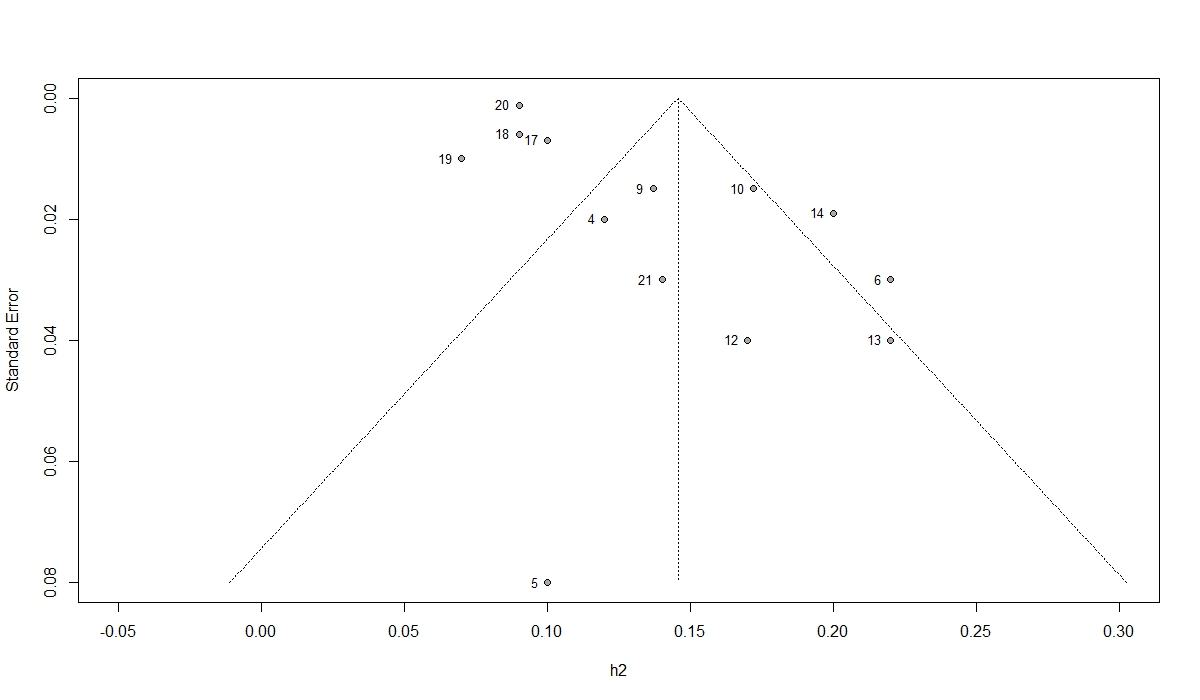


**Supplementary Figure S32.** The funnel plot in the meta-analysis of milking and general temperament scores. The code of the estimates were as follows: 1) 0.07±0.02 (Thompson et al., 1981), 2) 0.04±0.02 (Smith et al., 1985), 3) 0.08±0.02 (Foster et al., 1988), 4) 0.12±0.02 (Lawstuen et al., 1988), 5) 0.10±0.08 (Erf et al., 1992), 6) 0.22±0.03 (Visscher and Goddard, 1995), 7) 0.25±0.06 (Visscher and Goddard, 1995), 8) 0.33±0.06 (Cue et al., 1996), 9) 0.14±0.02 (Cue et al., 1996), 10) 0.17±0.02 (Cue et al., 1996), 11) 0.07±0.01 (Pryce et al., 2000), 12) 0.17±0.04 (Lassen and Mark, 2008), 13) 0.22±0.04 (Lassen and Mark, 2008), 14) 0.20±0.02 (Sewalem et al., 2011), 15) 0.38±0.07 (Kramer et al., 2013), 16) 0.04±0.04 (Kramer et al., 2013), 17) 0.10±0.01 (Stephansen et al., 2018), 18) 0.09±0.01 (Wethal et al., 2020), 19) 0.07±0.01 (Oliveira et al., 2021), 20) 0.09±0.01 (Szymik et al., 2021), 21) 0.14±0.03 (Taborda et al., 2023).


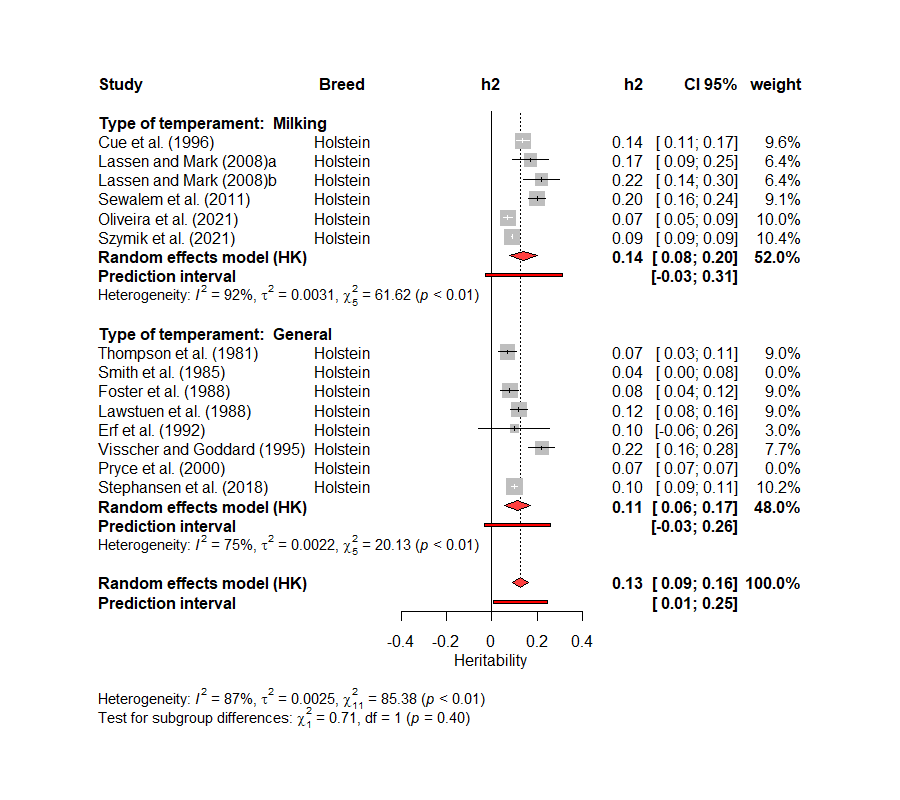


**Supplementary Figure S33**. Subgroup meta-analysis for dairy and milking temperament using only Holstein breed.

**
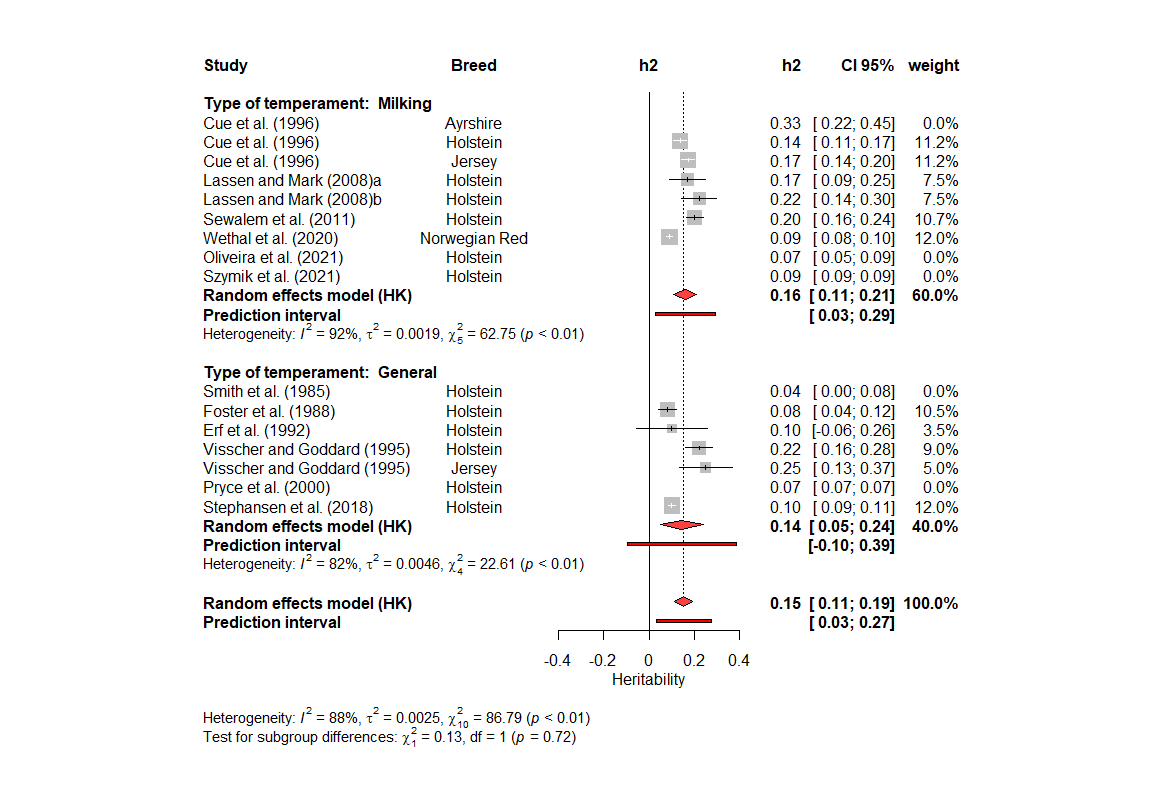
**

**Supplementary Figure S34**. Subgroup meta-analysis for dairy and milking temperament using only first lactation cows.
